# New Trends in Preparation and Use of Hydrogels for Water Treatment

**DOI:** 10.3390/gels11040238

**Published:** 2025-03-24

**Authors:** Teodor Sandu, Anita-Laura Chiriac, Anamaria Zaharia, Tanta-Verona Iordache, Andrei Sarbu

**Affiliations:** Advanced Polymer Materials and Polymer Recycling Group, National Institute for Research & Development in Chemistry and Petrochemistry ICECHIM, Spl. Independentei 202, 6th District, 060021 Bucharest, Romania; teodor.sandu@icechim.ro (T.S.); anita-laura.radu@icechim.ro (A.-L.C.); anamaria.zaharia@icechim.ro (A.Z.)

**Keywords:** hydrogel, water treatment, wastewater, pollutants, inorganic and organic dyes, heavy metals

## Abstract

Hydrogel-based wastewater treatment technologies show certain outstanding features, which include exceptional efficiency, sustainability, reusability, and the precise targeting of specific contaminants. Moreover, it becomes possible to minimize the environmental impact when using these materials. Their flexibility, low energy consumption, and adaptability to meet specific requirements for different purposes offer significant advantages over traditional methods like activated carbon filtration, membrane filtration, and chemical treatments. Recent advancements in hydrogel technology, including new production methods and hybrid materials, enhance their ability to efficiently adsorb contaminants without altering their biocompatibility and biodegradability. Therefore, innovative materials that are ideal for sustainable water purification were developed. However, these materials also suffer from several limitations, mostly regarding the scalability, long-term stability in real-world systems, and the need for precise functionalization. Therefore, overcoming these issues remains a challenge. Additionally, improving the efficiency and cost-effectiveness of regeneration methods is essential for their practical use. Finally, assessing the environmental impact of hydrogel production, use, and disposal is crucial to ensure these technologies are beneficial in the long run. This review summarizes recent advancements in developing polymer-based hydrogels for wastewater treatment by adsorption processes to help us understand the progress made during recent years. In particular, the studies presented within this work are compared from the point of view of the synthesis method, raw materials used such as synthetic/natural or hybrid networks, and the targeted class of pollutants—dyes or heavy metal ions. In several sections of this paper, discussions regarding the most important properties of the newly emerged adsorbents, e.g., kinetics, the adsorption capacity, and reusability, are also discussed.

## 1. Introduction

### 1.1. Water Pollution

Water is vital as it is a critical resource for humanity. However, we face a significant global strain, with only 40% of surface water bodies in the EU meeting a good ecological status [1]. The planet’s freshwater resources are steadily diminishing, which may be attributed mainly to population growth and the implicit rise in the water demand. The expanding industrial and agricultural sectors also contribute significantly to this matter as they consume vast amounts of water. Additionally, individual welfare improvements often lead to higher water consumption [2].

Water pollution is defined as the contamination of water by harmful substances that have a threatening effect on plants, animals, or humans. According to the Safe Drinking Water Act, a “contaminant” refers to any physical, chemical, biological, or radiological substance or matter in water. It is common for water bodies to contain small amounts of contaminants, many of which are harmless. However, some contaminants can be harmful when they exceed certain levels in the water. Contaminants’ presence does not necessarily involve a health risk [3]. For a contaminant to be classified as a pollutant, it must pose a significant threat to humans and the environment.

Water pollution, an immensely destructive problem the world must fight, may be attributed to the release of water pollutants, like, for instance, untreated waste, into the water bodies, thereby negatively impacting the water characteristics and quality. Polluted water can harm or kill plants and animals that depend on it, posing risks to humans and other organisms that consume it. Thus, the water pollutants are hazardous and must be managed appropriately [4]. Unsafe water and inadequate sanitation contribute to the prevalence of several diseases among the population [5,6,7]. Therefore, the sixth global goal of the United Nations, foreseen as part of its sustainable development agenda towards 2030, aims to ensure the availability and sustainable management of water resources [8].

Water pollution is classified into two main types: point source and nonpoint source pollution. Point source pollution occurs when dealing with a single, identifiable source, such as an individual factory. In contrast, nonpoint source pollution arises from multiple sources, such as several factories’ emissions [9].

### 1.2. Water Pollutants

Human activities and natural processes contribute to the discharge of pollutants into water, including organic compounds and nutrients (e.g., nitrogen and phosphorus), which can cause water eutrophication. Heavy metals such as Pb, Ni, Hg, and Cd pose environmental risks and threats to human health [1]. Water pollutants can be categorized into four main types: physical pollutants, chemical pollutants, biological pollutants, and radiological pollutants. Physical pollutants affect the physical appearance and properties of water and include suspended sediment or organic material, typically originating from soil erosion. Chemical pollutants are natural or man-made, that can contaminate water. Examples include fertilizers containing nitrogen or phosphorus, pesticides, salts, bleach, metals, bacterial toxins, and drugs from humans or animals. These pollutants are classified into organic and inorganic [4]. Organic compounds include (i) oxygen-demanding wastes, such as domestic and municipal sewage, wastewater from food processing, breweries, distilleries, canneries, slaughterhouses, tanneries, and paper mills, and (ii) synthetic organic compounds like synthetic detergents, pesticides, food additives, paints, synthetic fibers, pharmaceuticals, volatile organic compounds (VOCs), solvents [3], dyes, oils, and plastics [10]. Inorganic compounds consist of (i) fertilizers, (ii) heavy metals [10], and (iii) salts. Biological pollutants, or microbiological pollutants, are naturally occurring organisms in water, such as viruses, bacteria, protozoa, and parasites, that threaten humans and other life forms. The infectious diseases they cause, such as typhoid and cholera, are among the most widespread public health risks linked to drinking water. *Radiological pollutants* are chemical elements that release ionizing radiation, such as cesium, plutonium, and uranium, present on surfaces or within liquids, solids, or gases (including the human body), where their presence is unwanted or harmful.

### 1.3. Water Treatments

**Figure 1:**
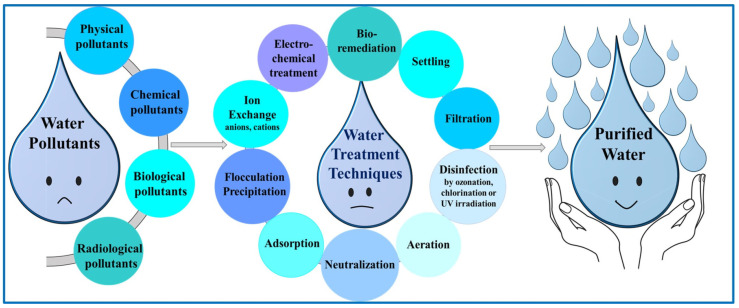
Major categories of water pollutants and the corresponding water treatment technologies used to obtain purified water.

Given water’s essential role in life, it is crucial to conserve water resources and treat wastewater for various uses. Sewage pollutants include organic contaminants, heavy metals, radioactive isotopes, and pathogens. Wastewater treatment methods are designed to remove these pollutants from the water (see Figure 1) [2].

Water treatment methods are selected according to the use of the treated water. They are divided into two main categories: water treatments for preparing drinking water and wastewater treatment to comply with the requirements of environmental regulations. Typically, specialized treatment methods are tailored for specific applications, such as agriculture or industry sectors.

Water treatment for drinking water typically involves several key processes to make water safe for consumption. Depending on the field of purified water use, the treatment stages are different. Thus, if we deal with water withdrawn from natural sources, it will follow these three main processes [11] to become suitable for drinking: (i). Settling, (ii). Filtration, and (iii). Disinfection by ozonation. During the settling step, the water undergoes initial filtration through two screens—a coarse sieve to capture large particles and a fine-mesh sieve to trap smaller ones. Next, a coagulant is added to the water to encourage suspended particles to clump together and form flocculates, which sink to the settling tank’s bottom. This process removes around 90% of suspended solids. Within the second step, filtration, the water flows through a series of filters, such as a fine sand bed, membranes, and/or activated carbon. Sand filtration eliminates visible particles, membrane filtration retains smaller particles, and activated carbon eliminates soluble pollutants like pesticides and detergents. Finally, a disinfection step is considered, which can be achieved in three ways: ozonation, chlorination, and UV irradiation. Ozone, known for its bactericidal and antiviral properties, is used to kill viruses, bacteria, and other microorganisms. It also acts on organic matter, improving the color and taste of the water. Chlorination prevents pathogen growth and maintains the water’s quality. In this case, chlorine is added to the water before leaving the production plant. UV light is an additional disinfection process to inactivate waterborne microorganisms without chemicals.

In some countries, drinking water is sourced from the sea or ocean through a process known as desalination. This technology removes salts and minerals from seawater to produce potable water. Methods such as reverse osmosis, vacuum distillation, multistage flash distillation, freeze–thaw, and electrodialysis are commonly used in saltwater desalination [12].

When dealing with wastewater treatment and the preparation of water for industry or agriculture, they include similar processes. These processes [13] may be divided into physical, chemical, and biological, each comprising several subclasses. Physical processes in turn can be divided into several categories: sedimentation, filtration, degasification, and aeration. Sedimentation involves separating an insoluble material from the water after it settles down at the bottom of the water layer [10,14]. Filtration is based on removing insoluble pollutants which are removed from the water, which occurs depending on their size [10,14]. Degasification involves the removal of dissolved gases that are removed from the solution, following Henry’s law [10]. Aeration involves treating the wastewater by air circulation through it, and it can occur by surface aeration, spray, and diffusion. This process is a physico-chemical process, because some pollutants are eliminated from the water, being taken off by the air flowing, and other pollutants are eliminated by their oxidation with oxygen [13]. Chemical processes can be grouped into six categories: neutralization, adsorption, precipitation, disinfection, ion exchange, and electrochemical treatments. Neutralization involves using an acid or a base to control and keep the pH level of the wastewater around 7 [13]. Adsorption involves the removal of soluble molecules from wastewater using different adsorbents and can be applied to various organic materials, such as toxic compounds, heavy metal actions, and detergents [10]. Precipitation is a method performed by injecting chemicals. It is sometimes used in conjunction with the temperature modification, the precipitate being removed by floatation, or sedimentation. It is also related to the flocculation [10,13]. Disinfection occurs following wastewater treatment using selected disinfection chemicals to inactivate the pathogens (viruses, microbes, and protozoa) [13]. An ion exchange removes anions, cations, or both are eliminated from the wastewaters through the use of ion-exchanging resins [10]. Electrochemical treatments are some of the essential processes for removing metals from wastewater [14]. Biological processes (bioremediation) can be carried out in three ways: aerobic treatment, anaerobic treatment, and phytoremediation; aerobic treatment occurs through a wastewater interaction with sunlight along with algae and bacteria. First, bacteria oxidize the organic pollutants, yielding carbon dioxide, water, and ammonia. Then, the algae grow by the utilization of inorganic waste producing a sludge which is separated by sedimentation [10,14]. Anaerobic treatment occurs by introducing sludge comprised of anaerobic bacteria in a bioreactor together with wastewater. These anaerobes digest the pollutants and produce contaminants with a low demand for oxygen, biogas, and sludge [15]. Phytoremediation involves the use of plants such as trees, bushes, and grasses, and smaller creatures remediate the wastewater through the accumulation and/or degradation of pollutants [14].

Recently, several advanced wastewater treatment processes were developed, including advanced oxidation, membrane filtration, and ultraviolet technology. Advanced oxidation is the elimination of pollutants using powerful oxidants, such as hydrogen peroxide or ozone [13]. Membrane filtration uses low- or high-pressure membranes to retain insoluble pollutants or high molecular substances through processes like microfiltration, nanofiltration, and ultrafiltration, while reverse osmosis membranes remove soluble low molecular pollutants (such as toxins and salts) [13,14]. Ultraviolet technology removes pollutants from water, air, and surfaces by applying high-energy electromagnetic ultraviolet radiation [13].

### 1.4. Hydrogels in Water Treatment

Hydrogels are soft materials characterized by three-dimensional (3D) polymer networks obtained by hydrophilic polymer physical or chemical crosslinking. These materials can expand in aqueous solutions, absorbing much water until the expansion force is entirely matched by the retraction force imposed by crosslinking [16].

According to the raw materials, hydrogels can be divided into synthetic and natural, with the latter agreeing better with the principle of sustainable development. Synthetic polymers used in hydrogels are typically non-biodegradable. They may exhibit certain health risks due to unreacted monomers, crosslinkers, and by-products produced during the polymerization [17,18]. The most widely used natural raw materials for hydrogels include cellulose, chitosan, sodium alginate, starch, proteins, and their derivatives. Additionally, hybrid hydrogels can be created by combining synthetic polymers with natural materials [19,20,21]. Composite hydrogels, on the other hand, are created by incorporating inorganic fillers such as silica, zeolite, or calcium carbonate into organic hydrogels [22,23,24].

Producing hydrogels from precursor polymers involves a crosslinking process that can be achieved through physical or chemical methods [25,26,27]. Physical crosslinking relies on hydrogen bonding, heating and cooling cycles, ionic interactions, freezing, or complex coacervation. Chemical crosslinking, on the other hand, involves approaches like reactions between complementary groups (e.g., using dialdehydes), grafting polymerization (particularly radical polymerization), irradiation, or enzyme-based processes. These methods enable the formation of the three-dimensional polymer networks that characterize hydrogels.

The main properties of hydrogels include the equilibrium swelling degree, mechanical properties, and chemical resistance. The equilibrium swelling of hydrogels refers to their capacity to absorb water. It is determined using Equation (1), where m_hydrogel_ is the weight of the equilibrium swollen hydrogels and m_xerogel_ is the weight of the dried hydrogels (xerogels) [28]. The mechanical properties are typically measured using esthesiometry and compression tests [29]. A dynamic mechanical analysis (DMA) is often used for this purpose [30].

(1) $$SD\left[\%\right]=\frac{\left(m_{hydrogel}-m_{xerogel}\right)}{m_{xerogel}}\times 100$$

An ideal hydrogel material should possess several functional features to meet the specific application requirements [31]. It should exhibit the highest equilibrium swelling, indicating a maximum adsorption capacity and an appropriate adsorption rate, which depends on factors such as the particle size and porosity. Additionally, the hydrogel should have the highest absorbency under load (AUL), be cost-effective, and contain minimal residual monomers. Furthermore, it should show high stability both during use and while being stored, along with excellent biodegradability to ensure that no toxic by-products are produced during degradation. The material should be colorless, odorless, and non-toxic, with a neutral pH after swelling. Other essential attributes include photostability and the ability to either release or retain the absorbed solution, depending on the specific application.

A hydrogel cannot simultaneously fulfil all the above-mentioned. Therefore, the reaction parameters must be carefully optimized for optimal properties. On the other hand, hydrogels used for drug or fertilizer delivery must be porous and capable of releasing the bioactive solution in a controlled manner [31].

Hydrogels find applications in various application fields, from industrial to biological [27]. In drug delivery, their porous architecture allows for high permeability to drug solutions, enabling controlled loading and gradually releasing them to the targeted organs. As scaffolds for tissue engineering, hydrogels are ideal materials due to their similarity to the extracellular matrix of many tissues; they can be delivered non-invasively and processed under mild conditions. Hydrogels meet the requirements for contact lenses to function effectively in different physiological environments. In sensors and biosensors, hydrogels serve as supports for immobilizing enzymes and other biostructures, offering an optimal environment to preserve the activity of bioelements used for biosensing. Furthermore, hydrogels show great potential in the producing of supercapacitors due to their excellent electrical properties, solid–liquid interface, and mechanical flexibility. In water treatment, hydrogels are excellent adsorbents for dyes, and hydrogel polyelectrolytes effectively remove heavy metal cations.

Nowadays, a wide range of adsorbent materials, such as activated carbons [32], biochar [33], zeolites [34], and metal–organic frameworks (MOFs) [35], are successfully used in wastewater treatment. However, in the case of emerging contaminants, which are more specific and complex, these adsorbent materials have certain limitations, such as a relatively low affinity and progressive reduction of the capacity for reuse or disposal. To address these challenges, new classes of adsorbents have been developed, and adsorbent hydrogels represent a promising solution.

Hydrogels have gained attention as adsorbent materials due to their distinct and unique properties, such as hydrophilicity, viscoelasticity, superabsorbency, and reusability, making the overall process more economical and sustainable [36,37]. The adsorption capacity of the hydrogel depends on its structural properties, such as the specific surface area, size, porosity, and the density of active sites, as well as on the properties of pollutants, their concentration, and the interactions between the contaminants. Certain essential structural features of an efficient hydrogel adsorbent, such as internal pores (also called voids), the pore size and distribution, and radial crosslinking density distribution, lead to highly effective adsorption mechanisms and improved mechanical stability. A balance between the pore size and distribution and an optimized crosslinking density distribution can contribute to the final hydrogel performance. Research on the internal voids, the space available between adjacent crosslinks in the polymer network, has played a crucial role in developing advanced porous hydrogels, significantly changing the final physical properties [38].

**Figure 2:**
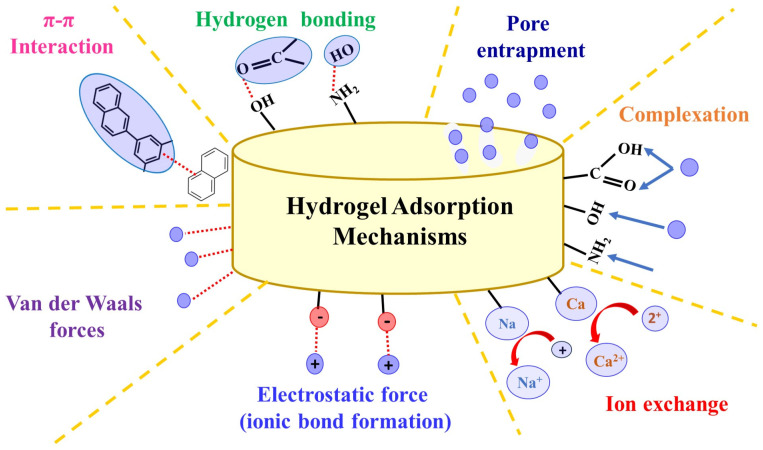
Adsorption mechanisms of the hydrogels for the removal of contaminants.

Different mechanisms can be involved in adsorbing contaminants in wastewater. The hydrogel–pollutant interaction can be determined by combining several adsorption mechanisms (Figure 2). Electrostatic interactions, an ion exchange, and complexation are the key mechanisms for the adsorption of heavy metals [39]. The adsorption mechanisms of organic dyes, agrochemicals, and other organic molecules occur through π–π interactions, H-bonding, and van der Waals forces. Another possible adsorption mechanism that can happen in hydrogels is based on pore filling. Pollutant molecules can be physically trapped in the pores of the hydrogel. Therefore, the types of adsorption mechanisms depend primarily on the porosity of the hydrogel and the targeted pollutant molecules.

The adsorption process using hydrogels offers several advantages, including cost-effectiveness, a variety of readily available adsorbents, regenerable adsorbents, a large capacity, scaling up high speed, and simple operation compared to other techniques, such as chemical precipitation, ion exchange, coagulation–flocculation, flotation, and membrane filtration. However, the process has limitations: the generation of secondary residues that require treatment before disposal, sometimes a high production cost of some absorbent materials, and possible chemical-intensive and energy-intensive regeneration [40].

The improper disposal of a pollutant-laden hydrogel can lead to numerous problems, especially secondary pollution, which can have serious environmental and human health consequences. In addition, non-recycled or inadequately destroyed hydrogels can contribute to the formation of persistent waste, especially synthetic hydrogels, by forming microplastics obtained during the degradation process that can have long-term adverse effects.

To effectively use hydrogels in industrial and large-scale applications as adsorbents for removing contaminants from wastewater, they must be able to desorb, regenerate, and be reusable. Primarily, the desorption process does not have to cause any damage to the hydrogel, and the regeneration process for the adsorbent should be cost-efficient. Secondarily, after the adsorption and desorption processes are performed, the degradation of the hydrogels cannot be observed. These properties are essential for the efficiency and sustainability of the dyes and heavy-metal-removal process [41].

Furthermore, the regeneration of hydrogels remains a challenge, often leading to a decrease in the adsorption efficiency over multiple cycles. The post-treatment process is essential to consider in the hydrogel’s regeneration used in wastewater treatment. The hydrogel should successfully release the contaminants by a desorption or regeneration step without compromising the chemical and physical stability of the polymer matrix [42]. The efficiency of regeneration depends on the hydrogel’s chemical composition, the nature of the retained contaminates, and the adsorption mechanisms responsible for the hydrogel−contaminants interactions. As we have observed in most of the research, the post-treatment process involves chemical techniques, such as immersing the saturated hydrogels in an eluent to recover the contaminants in the liquid phase [43]. The most common and used desorption solutions in the literature are acidic (HCl, H_2_SO_4_, and HNO_3_ 0.1–1 M) or alkaline (NaOH, KOH, NaHCO_3_, or NH_4_OH 0.1–1 M) eluents for the regeneration of hydrogel-based adsorbents. The desorption agents break the hydrogel−contaminates interaction (such as electrostatic, complexation, or Van der Waals forces), while the hydrogel structure remains intact for successive adsorption cycles [44,45,46,47,48,49]. It is also important to mention that the regeneration process of hydrogels has specific advantages, such as recovery and reuse, but also drawbacks, such as the relatively high consumption of water and energy. All these aspects must be understood and considered to select the most appropriate design and application methods in real life.

**Figure 3:**
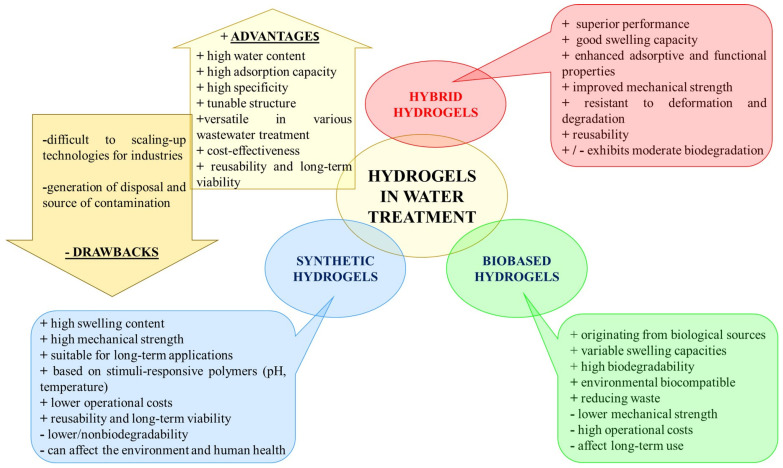
Characteristics of synthetic, biobased, and hybrid hydrogels for water treatment.

Therefore, it is essential to develop efficient methods for regenerating and reusing hydrogels, thus reducing the need for their disposal. In addition, using biodegradable polymer-based hydrogels or those capable of regeneration through controlled chemical and physical processes can contribute to a more sustainable and ecological approach to wastewater treatment. Biodegradable polymer-based hydrogels are ideal for short-term wastewater treatment, as they decompose after the first use, thus reducing the environmental impact. On the other hand, non-biodegradable synthetic polymer-based hydrogels are suitable for long-term applications, supporting sustainability, provided they are recyclable. The design and development of hydrogels with tunable degradation rates must maintain their functionality over the intended lifetime, but also ensure their degradation when no longer functional [50]. In this context, hybrid hydrogel systems present a promising solution. Also, their durability can be improved without compromising the environmental safety by reinforcing biodegradable hydrogels through the physical crosslinking process or natural inorganic nanoparticle encapsulation. Future research on developing and designing innovative biohybrid hydrogels based on natural polymers with dynamic crosslinking networks will lead to promising new methods to achieve both durability and biodegradability, ensuring performance and environmental safety [51]. Figure 3 summarizes some characteristics of synthetic, biobased, and hybrid hydrogels that can be considered when deciding to use them as adsorbents for wastewater treatment, allowing a comparative assessment on their potential for industrial applications and on the commercialization challenges.

Hydrogels are key materials for treating wastewater due to their versatile properties and treatment efficiency. This review focuses on significant breakthroughs in water pollution amendment, emphasizing the most performant hydrogels used for water/wastewater treatment. It also provides an overview of synthetic hydrogels, with a growing focus on natural and semi-synthetic alternatives, which support sustainable wastewater purification technologies. These materials proved viable in mitigating major water pollutants, including heavy metals and dyes. Although several reviews address the topic of hydrogels in water treatment, this work aims to bridge a critical gap by thoroughly examining the origins, preparation methods, unique properties, and innovative designs of next-generation hydrogel adsorbents for sustainable water and wastewater treatment. Furthermore, this review will focus on the performance of these hydrogel adsorbents and their methods of obtaining, especially their specific applications in water treatment. Moreover, insight is provided into selecting raw materials, considering the targeted water treatment approach, and customizing hydrogel properties to meet specific requirements.

## 2. Synthetic Polymer-Based Hydrogels for Water Treatments

Hydrogels for water treatment can be divided into three main categories depending on their origin: synthetic polymer-based hydrogels, bio-based hydrogels, and hybrid hydrogels. Synthetic polymers are manufactured from petroleum-based monomers and are chemically and mechanically more potent than natural polymers/polymers/naturally occurring bio-polymers. The use of synthetic polymers in hydrogel production has increased significantly since the groundbreaking work of Wichterle and Lim [52]. Nowadays, synthetic polymers play a significant role in our daily lives, with global production reaching nearly 330 million tons per year. Over the past 10 years, the production and use of synthetic polymers have increased exponentially. This growth is attributed to the social benefits of these materials, particularly those related to sustainability [53]. Depending on the composition of monomers, the synthetic polymers may cover a wide range of properties (i.e., flexibility, strength, stiffness, density, heat resistance, and electrical conductivity) and applications (i.e., drug carriers, coating, absorbents, packaging, textiles, laboratory equipment, automotive parts, industrial fibers, food containers, and so on) [54].

**Figure 4:**
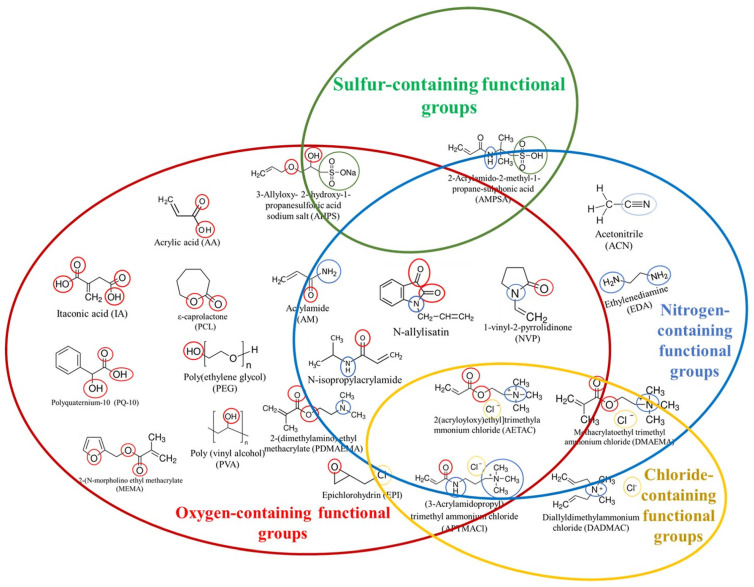
Synthetic monomer/polymer units based on heteroatoms functional groups.

Synthetic polymer-based hydrogels have attracted attention in water treatment owing to their attractive properties, such as their high adsorption capacity and rate, high stability and durability during swelling and storage, longer shelf life, less external reaction, greater adhesion, good mechanical strength, cell-specific bioactivities, regeneration–reuse capability, abundance, and cost-effectiveness [55]. Synthetic hydrogels are synthesized from a wide range of monomeric and/or polymeric units such as acrylic acid (AA) [56], methacrylic acid (MAA) [57], N-isopropyl acrylamide (NIPAM) [58], acrylamide (AM) [59], polyethylene glycol diacrylate (PEGDA) [60,61], polyvinyl alcohol (PVA) [62], 2-acrylamide-2-methylpropane sulfonic acid (AMPSA) [63], and itaconic acid [64]. This synthetic monomer/polymer basic units can be crosslinked by different physical (hydrogen bonding, a hydrophobic interaction, ionic interaction, π–π interaction, metal–ligand complexation, aggregation of polymer chains, and crystallization) or chemical (chain-growth polymerization, addition polymerization, condensation, click reaction, Schiff base-promoted, enzyme-catalyzed) mechanisms to create a three-dimensional polymeric network. The most common crosslinkers used in the synthesis of synthetic polymer hydrogels are N,N-methylenebisacrylamide (MBA), ethylene glycol dimethacrylate, 1,1,1-trimethylolpropanetrimethacrylate, tetramethylenediamine, and glyoxal [65]. They carry specific heteroatom-bearing functional groups (e.g., -OH, -COOH, -NH_2_, -CONH_2,_ -SO_3_H, and -CH_2_-Cl) that enable the adsorptive removal and recovery of pollutant molecules such as heavy metals, organic materials, and emerging contaminants through physicochemical interactions, making them valuable for water purification and wastewater treatment. Figure 4 depicts the synthetic monomer/polymer units highlighting the heteroatoms containing functional groups that have been used in water/wastewater treatments over the past decade.

Synthetic polymers are not biocompatible or biodegradable and persist in the environment for a long time, leading to controversial debates over potential environmental and public health risks from scientific, socio-economic, and political perspectives [66]. However, synthetic polymer-based hydrogels typically have well-defined structures and can be modified to provide degradability. Recently, considerable research has been focused on developing sustainable synthetic polymer-based hydrogels with superabsorbent features, high removal efficiency, and fast adsorption rates for water purification and wastewater treatment [67,68].

Understanding the unique properties of synthetic polymers plays a vital role in designing and developing novel hydrogel adsorbents for sustainable water treatment. The careful selection and use of suitable polymers are crucial for preparing novel hydrogel absorbents with excellent mechanical properties, a high adsorption performance, environmentally friendly, biodegradable, reusability, and cost-effectiveness. Furthermore, the preparation methods also influence the morphology of the final hydrogel (in terms of the sizes and shapes of the pores and adsorption capacity) [69]. Thereby, this chapter thoroughly overviews the most recent advancements in using synthetic polymer-based hydrogels for water treatment. We cover preparation methods, unique characteristics, and innovations in developing next-generation hydrogel absorbents for sustainable water/wastewater treatment. The latest studies in their utilization for removing and recovering heavy metals and toxic dyes are mainly discussed.

### 2.1. Retention of Organic Dyes Using Synthetic Polymer-Based Hydrogels

Organic dyes are a major source of water pollution, with a global production volume estimated at 1 million tons annually and significant quantities discharged into the environment, urging effective and timely remediation to minimize the environmental impacts [70,71]. They are known for their intense colors and are extensively used in various fields, including textiles, printing, plastics, paper, rubber, leather tanning, food, pharmaceuticals, and cosmetics. Based on their ionic nature, organic dyes are classified into cationic, anionic, and non-ionic based on their ionic nature. Most of them are toxic, non-biodegradable, carcinogenic, mutagenic, and bactericidal, damaging the aquatic environments and threatening the living organisms’ health [72]. Therefore, the targeted removal of organic dyes has been receiving increasing scientific attention. Many adsorbent materials, such as activated carbon [73], biochar [74], graphene oxide [75], zeolite [76], metal–organic frameworks [77], covalent organic frameworks [78], and hydrogels, have been utilized to remove organic dyes from wastewaters.

Hydrogels’ adsorbent materials have gained significant attention for the dye’s retention from wastewater. Hydrogels are preferred for their high removal efficiency, which ranges from 80% to 99% [79,80]. Furthermore, adsorbents based on synthetic polymeric hydrogels can be modified and functionalized with different attached moieties to provide the required physical and chemical properties and enable selective dye retention [81]. In addition, synthetic polymer-based hydrogels can be regenerated and reused for a few cycles, making the overall dye-removal process economically feasible [44].

#### 2.1.1. Polyacrylate Hydrogels and Their Polymer Blends for Efficient Organic Dye Retention

In this context, Table 1 summarizes a brief overview of recently developed synthetic polymer and hybrid synthetic polymer-based hydrogels for organic dye retention, pointing out their preparation methods, performance, and reusability. Among the various synthetic polymers used for dye retention, polyacrylates were paid peculiar attention due to their high absorbency and ability to modify interactions with different dyes, which renders them highly effective for dye control and removal. Adsorption and reuse studies have shown that polyacrylates-based hydrogels can act efficiently in water and wastewater treatment. These materials contribute to environmental remediation, but also facilitate the recovery of dyes, bringing about their industrial reuse [82,83,84,85]. Some examples of dye removal applications using polyacrylate-based hydrogels and blends with other polymers developed over the last decade are listed below.

The Dalalibera group [88] synthesized a polyacrylic acid-based (PAA) hydrogel through free-radical polymerization, aiming for the adsorption, removal, and simultaneous selective separation of synthetic dyes, including methylene blue (MB) and methyl orange (MO) dyes from an aqueous medium. They selected N, N’-methylenebisacrylamide (MBA) as the crosslinking agent and ammonium persulfate (APS) as the initiator for synthesizing the PAA hydrogel. At pH 8 and a temperature of 39.85 °C, using a dried hydrogel mass of 100 mg, a solution volume of 50.0 mL, and a contact time of 3500 min, the adsorption capacity of the PAA hydrogel for MB was 454.45 mg/g. The PAA hydrogel also showed good recyclability within five successive adsorption–desorption cycles. However, the concentration of the anionic dye, methyl orange (MO), did not decrease under these conditions. The desorption efficiency for acid MB dyes was 87.76% after five cycles.

Mohamed A. Mekewi et al. [86] fabricated a hydrogel based on acrylic acid (AA) and acrylamide (AM) through free-radical polymerization, designed to serve as an adsorbent for the removal of the cationic dye Methylene Blue (MB) from water. The resulting hydrogel, enriched with carboxylic groups, exhibited negatively charged, smooth surfaces that featured distinctive ulcer-like pores, which played a crucial role in dye removal from wastewater and showed excellent reproducibility. The amide groups within the hydrogels facilitated electrostatic and chemical interactions with the dye molecules. The hydrogel adsorbed MB with a capacity that reached 1315 mg/g under the following conditions: a reaction temperature of 25 °C, initial dye concentration of 50 mg/L, adsorbent dosage of 40 mg, and a pH of 8, with a contact time of 12 h. The kinetic analysis followed the pseudo-second-order model (R^2^ = 0.99). Furthermore, the Dubinin–Radushkevich isotherm model best described the adsorption data. The kinetics of adsorption were primarily influenced by the internal diffusion rate of MB molecules into the hydrogel’s ulcer-like holes, which effectively trapped the dye molecules from the solution, resulting in rapid initial adsorption rates.

Sakthivel et al. [87] developed novel pH-sensitive polymeric hydrogels based on itaconic acid (IA) and acrylic acid (AA) through a cost-effective methodology as promising green materials for removing cationic dyes. The researchers used methylene blue (MB), a cationic dye, as a model in their study. The process started with a pre-polyester by reacting IA and ethylene glycol (EG), followed by a condensation reaction with AA through free-radical polymerization, using potassium persulfate (KPS) as an initiator. IA, known as an unsaturated dicarboxylic acid, quickly copolymerizes and provides polymer chains with carboxylic acid groups, which are highly hydrophilic bonds capable of forming new hydrogen bonds, which significantly enhances the hydrogels’ dye-removal ability. The IEA pH-sensitive polymer hydrogels achieved a dye removal efficiency of up to 83% and an adsorption capacity of 1270 mg/g at a pH of 10. The authors recommend IEA-based hydrogels as environmentally friendly materials for cationic dye removal, wastewater treatment, and water purification applications.

Thakur et al. [88] investigated the effective removal of Rhodamine B (RhB), a cationic dye, using an itaconic acid-grafted poly (acrylic acid-co-aniline) (IA-g-poly (AA-co-ANi)) hydrogel as an adsorbent. Under optimum conditions (with an adsorbent dose of 75 mg, 30 mL of 50 mg/L RhB solution, a pH of 7, a temperature of 25 °C, and a contact time of 60 min), the removal efficiency for RhB was 87.9%. Additionally, the hydrogel adsorbed RhB with a capacity of 925.92 mg/g. The synthesized IA-g-poly (AA-co-ANi) hydrogel was reused in four successive adsorption–desorption cycles without a noteworthy decrease in its performance with a removal efficiency of 85.2%. Kinetic and adsorption isotherm models for the RhB dye followed the pseudo-first-order model and Freundlich isotherm model, respectively.

**Figure 5:**
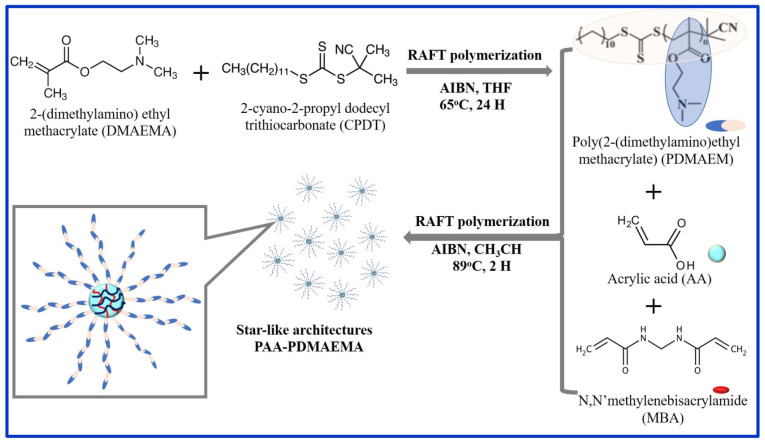
The synthesis steps of star-like architectures of polyampholyte-based hydrogels based on PAA and PDMAEMA (Adapted from Ref. [89]).

Recently, Mohammadzadeh and co-workers [89] developed polyampholyte-based hydrogels using poly (acrylic acid) (PAA) and poly(2-(dimethylamino) ethyl methacrylate) P (DMAEMA) with star-like architectures through two-step reversible addition-fragmentation chain transfer (RAFT) polymerization (see Figure 5). The star-like structures were confirmed by proton nuclear magnetic resonance, gel permeation chromatography, and differential scanning calorimetry. The synthesized polyampholyte-based hydrogels were tested for their ability to remove methylene blue (MB) and methylene orange (MO) dyes from wastewater. The hydrogels adsorbed MB with a maximum capacity of 38.6 mg/g and an efficiency of 48.3%, at pH 10, MO with a capacity of 61.2 mg/g, and an efficiency of 76.6% at pH 2. Furthermore, the kinetic analysis followed the pseudo-second-order model, and the adsorption data were best described by the Freundlich isotherm model, suggesting that the hydrogels facilitate the non-uniform chemical adsorption of dyes onto their heterogeneous sites.

#### 2.1.2. Sulfonic Acid Hydrogels Based on Synthetic Polymers for Efficient Organic Dye Retention

Sulfonate acid (-SO_3_H) groups are essential for adsorbing dye molecules in polluted water. The negatively charged sulfonate groups in sulfonic acid monomers are often incorporated into hydrogel networks to enhance their ion exchange capacity, hydrophilicity, and strong acidic nature, making them particularly effective for adsorbing cationic dyes [90,91,100]. In this respect, Shoueira et al. [92] developed macro- and nanogels with semi-interpenetrating polymer networks (semi-IPNs) based on poly (vinyl alcohol) and a copolymer of 2-acrylamido-2-methyl-1-propane-sulfonic acid and acrylic acid [PVA@P(AMPS-co-AAc)], through free-radical crosslinking copolymerization, for the removal of methylene blue (MB) cationic dye from wastewater. The macro- and nanogel materials adsorbed MB with maximum capacities of 416.16 mg/g and 181.8 mg/g, respectively, at pH 6 and 25 °C. Furthermore, the hydrogels could be reused in up to six cycles without significantly decreasing their removal efficiency.

#### 2.1.3. Quaternary Ammonium Hydrogels for Efficient Organic Dye Retention

Some hydrogels have been developed as adsorbents based on quaternary ammonium compounds (R_4_N^+^)^−^. Quaternary ammonium-based hydrogels have demonstrated remarkable capabilities in adsorbing anionic dyes from aqueous solutions [93,94,95,96]. The cationic nature of the R_4_N^+^ groups on the hydrogel allows them to attract and bind strongly to negatively charged dye molecules through electrostatic interactions. They exhibit high selectivity, a large adsorption capacity, and a rapid adsorption rate. Moreover, the bond formed with dye molecules is strong and resistant, making the adsorption process relatively stable but challenging to desorb, limiting the reusability of the hydrogel without specific chemical treatment to break the bonds.

In a recent study [97], a novel intelligent ampholyte hydrogel (IAH) was prepared by the free-radical redox polymerization of acrylamide (AM) with acrylic acid (AA) and 2-(acryloyloxy)ethyl trimethylammonium chloride (AETAC) as the monomers, ethylene glycol dimethacrylate as a crosslinker, and ammonium persulfate (APS) and N,N,N,N-tetramethyl ethylenediamine (TEMED) as the initiation system. This intelligent ampholyte hydrogel was designed to be an efficient adsorbent for removing dyes from water. The IAH adsorbed anionic carminic acid (CA), crystal violet (CV), and methyl violet (MV) with a maximum capacity of 2.445 mg/g, 0.1564 mg/g, and 0.1151 mg/g, respectively. The authors observed that the adsorption of dyes onto IAN occurred by electrostatic interactions and the H-bonding.

In the continuous use of quaternary ammonium-based hydrogels for the anionic dye removal process, Li et al. [98] prepared quaternary ammonium hydrogels based on a cationic absorbent poly (epichlorohydrin)−ethylenediamine hydrogel (PEE-Gel) through a one-step copolymerization process, designed to serve as an efficient adsorbent to remove dyes from water. The removal efficiency for anionic dyes as Direct Red 23 (DR23) exceeded 99%, and the maximum adsorption capacity was 1540.19 mg/g under optimal conditions, which included an adsorbent dose of 200 mg/L, a temperature of 40 °C, a processing time of 4 h, and DR23 concentrations ranging from 100–440 mg/L. The adsorption capacity was higher than that of most other available adsorbents. Yet, the adsorption of dyes onto PEE-Gel was relatively stable, making regeneration challenging.

**Figure 6:**
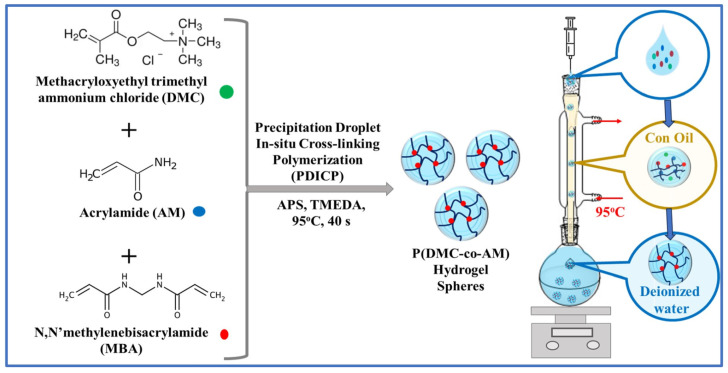
The polymerization process and schematic diagram of the device (Adapted from Ref. [99]).

In an interesting study, Liu’s group [99] developed poly (methacrylatoethyl trimethyl ammonium chloride-co-acrylamide)-P (DMC-co-AM) hydrogel particles, demonstrating the exceptional and selective removal of anionic dyes. This study eliminated the need for hazardous solvents and the generation of toxic by-products. They used a fast and environmentally friendly approach via precipitation droplet in situ crosslinking polymerization, utilizing corn oil and deionized water. The synthesis process and schematic diagram of the P (DMC-co-AM) device are shown in Figure 6. The hydrogel particles (PCM5-1 HPs), with the mass ratio of DMC to AM of 5:1, showed the best comprehensive performance over the mechanical strength and adsorption properties. Thus, the adsorption capacities of hydrogels surpassed those of most previously reported hydrogel adsorbents in the literature, with specific values of 992.63 mg/g for Orange G (OG), 1388.55 mg/g for Alizarin Red (AR), and 744.17 mg/g for Methyl Orange (MO), and the removal ratios of anionic dyes reached more than 94%. The kinetic and adsorption isotherm models were pseudo-second-order and Langmuir, respectively. Furthermore, the hydrogel particles maintained a high adsorption capacity across a broad pH range of 3 to 9, indicating good applicability in acidic and alkaline environments. They also demonstrated practical reusability for up to five desorption–resorption cycles, with a desorption ratio higher than 80%, indicating an excellent regeneration capacity.

#### 2.1.4. Nanocomposite Hydrogels Based on Synthetic Polymers for Efficient Organic Dye Retention

**Figure 7:**
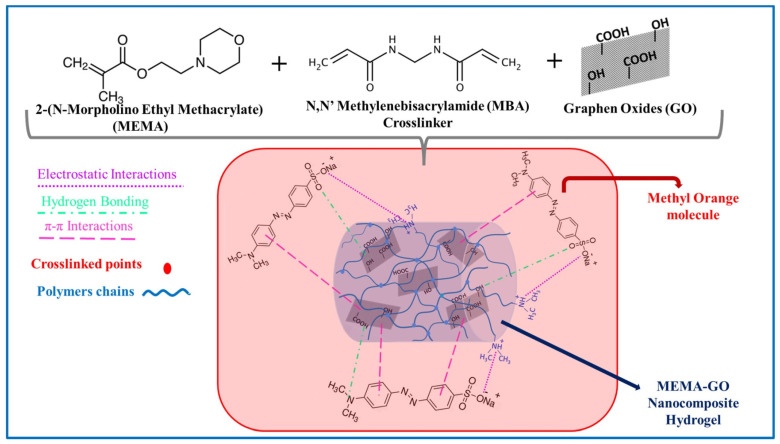
The schematic mechanism of adsorption of MeO dye molecules onto the MEMA-GO nanocomposite hydrogel (adapted from Ref. [112]).

The mechanical and thermal limitations of hydrogel-based adsorbents have led to research on their nanocomposite derivatives, which incorporate inorganic nanoparticles dispersed within a synthetic polymer network. Adding nano-sized fillers, such as graphene oxide [101], biochar [102], activated carbon [103], different metals [104,105,106], clays [45,107], and carbon nanotubes, significantly enhances the mechanical and thermal properties of these materials. Various nanocomposite hydrogel-based adsorbents have been synthesized and applied to remove dyes from aqueous solutions [46,47,108,109,110,111]. Taktak and Gokçe [112] synthesized a novel nanocomposite hydrogel based on 2-(N-morpholino ethyl) methacrylate/graphene oxide (MEMA@GO) and evaluated its ability to remove (MO) dye. GO enhanced the final hydrogel’s surface area and mechanical and thermal properties, resulting in a porous structure that remains thermally stable up to 280 °C. The maximum MO removal was achieved up to 199.67 mg/g under optimal conditions using a 10 wt% GO content at pH 2, an initial dye concentration of 50 mg/L, and a contact time 510 min at 25 °C. It was found that the adsorption efficiency was enhanced by increasing the GO content. This significant increase in the adsorption capacity is related to the strong interaction of the GO nanosheets with the MO molecule, including the -COOH and -OH groups of GO and hydrogen bonds formed between the nitrogen and oxygen groups of MO, as well as the π–π interactions between their aromatic rings (as illustrated in Figure 7). The kinetic and isotherm data indicated the removal of MO dye followed by the pseudo-second-order and Langmuir models. Moreover, MEMA@GO nanocomposite hydrogel showed good recycling, maintaining a removal efficiency of 96.8% over eight consecutive cycles. In addition, the nanocomposite hydrogel-based adsorbents showed high biodegradability under soil conditions, with a mass loss of 72% [112].

In another research, Mosaffa et al. [113] achieved a high adsorption capacity for two cationic dyes using a PAA-based composite hydrogel adsorbent using biochar with a curcumin-modifying agent via an in situ free-radical polymerization method. They found that the adsorption capacity for Malachite Green (MG) and Rhodamine B (RhB) dyes was 521 and 741 mg/g, respectively. Also, in a binary system, the adsorption test demonstrated the higher efficacy of the synthesized adsorbent in removing MG than RhB. The results were well supported by the Koble–Corrigan and Langmuir isotherm models (R^2^ > 0.997) as well as pseudo-second-order (R^2^ > 0.998) and Elovich (R^2^ = 0.983 and 0.995) kinetics models, which closely aligned with the empirical findings for both dyes. Finally, the adsorption–desorption study revealed that the adsorbent could be reused without a significant loss of the removal efficacy, maintaining about 80% efficacy over seven successive cycles.

In the ongoing development of nanocomposite hydrogels for dye removal, Yang’s group [114] developed a poly (acrylic acid-co-polyvinylpyrrolidone)/Palygorskite (PGS)-(PAPP) nanocomposite hydrogel for the adsorption of MB dye through free-radical copolymerization. Polyvinylpyrrolidone (PVP) is a low-toxicity, biocompatible, biodegradable, water-soluble, and uncharged polymer. Due to O and N atoms in its structure, it can form a complex with dyes [87]. The nanocomposite hydrogel demonstrated a removal efficiency of 97%, achieving a maximum adsorption capacity of 1815 mg/g for MB. Kinetics and isotherm studies revealed that MB dye was adsorbed onto the developed PAPP nanocomposite hydrogel via a pseudo-second-order model and aligned well with the Langmuir sorption isotherm, exhibiting regression coefficients (R^2^) greater than 0.999.

Wen et al. [103] developed an innovative gel adsorbent using a simple one-pot approach by the dropwise addition of an aqueous mixture of poly (vinyl alcohol) (PVA), activated carbon (AC), and iron ions into the ammonia solution. Iron ions reacted with the ammonia solution and formed magnetic Fe_3_O_4_ nanoparticles, also serving as a physical cross-linking agent to gelate the PVA macromolecules. Poly (vinyl alcohol) (PVA) is a biodegradable, water-soluble synthetic polymer used to remove organic dyes from wastewater [115]. The resulting magnetic PVA/AC composite gel (mPVA/AC CG) was designed to adsorb methylene blue (MB) and methyl orange (MO) dyes from water. It exhibited good adsorption capacities (174 mg/g for MB and 147 mg/g for MO) and an efficient magnetic separation capability, offering a promising approach for creating practical magnetic hydrogel adsorbents.

At the end of this section, the outstanding results from the past decade of studies on nanocomposite hydrogels as dye adsorbents are summarized in Table 2.

### 2.2. Synthetic Polymer-Based Hydrogels for Heavy Metals Retention

Heavy metals are defined as metals with an equal or greater density of 5 g/cm^3^ and represent another significant source of water pollution. These metals are toxic and harmful even at low concentrations. Water contamination by heavy metals occurs through natural processes, such as oil erosion, dissolved salts from rainfall, and industrial and urban activities [72]. Common heavy metals include platinum group metals, lead (Pb), mercury (Hg), cadmium (Cd), arsenic (As), iron (Fe), tin (Sn), silver (Ag), and radionuclides heavy metals such as uranium (U), radium (Ra), thorium (Th), and americium (Am). Many of these metals are toxic when present above acceptable levels, threatening ecosystems and human health. Therefore, there has been increasing scientific focus on developing efficient methods for their removal. This part of the review will examine significant findings on using synthetic polymer-based hydrogel adsorbents to remove heavy metals, as collected in Table 3.

Acrylic acid (AA), acrylamide (AM), itaconic acid (IA), 2-acrylamide-2-methylpro pane-sulfonic acid (AMPS), N-isopropyl acrylamide (NIPAM), N-vinyl-2-pyrrolidone (NVP), among others, are synthetic monomers commonly employed in the synthesis of hydrogels for heavy metal adsorption [116,117,118,119]. According to the literature, acrylic monomers are primarily used to adsorb heavy metals due to the presence of carboxylic groups (R-COOH), which play a significant role in the adsorption and swelling properties of hydrogels [72]. However, the carboxyl group (R-COOH) by itself does not show the highest adsorption activity towards metal ions. Consequently, Lv et al. [120] synthesized a polyacrylic acid-based (PAA) hydrogel containing COO^−^/COOH functional groups via free-radical polymerization. The hydrogel was designed for the adsorption and removal of trace-level metal ions, including Cu^2+^, Cd^2+^, Cr^6+^, Fe^3+^, Mn^2+^, Ni^2+^, Zn^2+^, Ce^3+^, and Ag^+^ from an aqueous medium. Calcium hydroxide Ca(OH)_2_ nano-spherulites (CNS) were employed as a crosslinking agent, while ammonium persulfate (APS) and N,N,N′,N′-tetramethylethylenediamine (TEMED) were employed as the initiation system for the hydrogel synthesis. The resulting PAA hydrogel exhibited an exceptional swelling ratio of up to 8310 g/g and demonstrated high removal efficiencies for trace metal ions: 46.8% for Cu^2+^ (initial concentration C_0_ = 0.16 mg/L), 57.1% for Cd^2+^ (C_0_ = 0.56 mg/L), 26.9% for Cr6^+^ (C_0_ = 0.52 mg/L), 5.3% for Fe^3+^ (C_0_ = 0.56 mg/L), 52.7% for Mn^2+^ (C_0_ = 0.55 mg/L), 52.5% for Ni^2+^ (C_0_ = 0.59 mg/L), 58.4% for Zn^2+^ (C_0_ = 0.65 mg/L), 70% for Ce^3+^ (C_0_ = 0.7 mg/L), and 45.3% for Ag^+^ (C_0_ = 1.08 mg/L). Additionally, the hydrogel demonstrated excellent reusability over ten adsorption–desorption cycles, maintaining an adsorption capacity (Q_e_) of up to 104 mg/g and retaining approximately 70% of its initial adsorption capacity.

**Figure 8:**
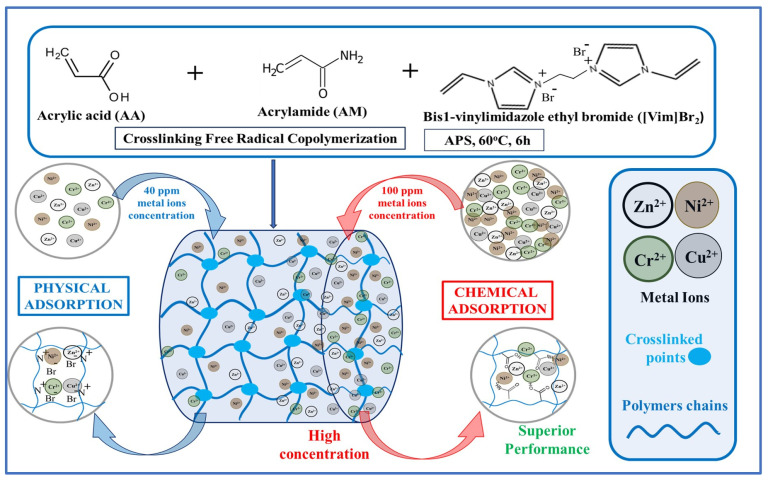
The synthesis process and the schematic illustration of the mechanism of adsorption for heavy metal ions onto the PAA-AM-VimBr_2_ hydrogel (adapted from Ref. [122]).

Acrylic-based polyacrylamide hydrogels also represent a good option for preparing hydrogel adsorbents for the removal of heavy metal ions [121]. Acrylamide (AM) is a hydrophilic polymer with an amide group that contains a nitrogen atom with a lone pair of electrons, which can readily attach to cationic metal ions. Sun et al. [122] reported on the use of acrylic-based acrylamide hydrogels combined with ionic liquids, specifically vinyl imidazole bromide [Vim]Br_2_, as crosslinking agents for the adsorption of heavy metal ions such as Ni^2+^, Cu^2+^, Zn^2+^, and Cr^3+^ from water. Based on the electrostatic interaction of the bromide ions from the crosslinking ionic liquid agent in the hydrogel structure, the removal rates (RR%) of the heavy metal ions were enhanced, with RR% values all exceeding 90% (see Figure 8).

Maksoud’s group [123] synthesized polyacrylic acid-co-polyacrylamide p (AAm-co-AAc) hydrogel-based adsorbents with different functionalities (sulfonate, carboxyl, thiol, hydroxyl, and amine groups) for the effective removal of the metal ions Cu^2+^, Ba^2+^, and Sr^2+^ from natural and low-saline water solution. They used gamma irradiation to control the crosslinking polymerization of acrylamide (AM) and acrylic acid (AA) chains, obtaining a 3D hydrogel network with a high porosity and swelling capacity. The adsorption studies reveal that all modified hydrogels exhibited a higher removal efficiency for the three metal ions than unmodified hydrogels, validating the key role of surface functionalities in tailoring hydrogel affinity for metal ions adsorption. Amongst these, the NaOH-treated hydrogel demonstrated a superior performance, achieving maximum adsorption capacities of 13.67, 36.4, and 27.31 mg/g for Cu^2+^, Ba^2+^, and Sr^2+^ ions, respectively. Moreover, the adsorption behavior closely followed the Freundlich isotherm models (R^2^ = 0.90–0.99) as well as the pseudo-first-order (R^2^ > 0.99) kinetics model, aligning well with the empirical data for the three metal ions.

2-Acrylamido-2-methyl-1-propanesulfonic acid (AMPS) is an ionic and highly hydrophilic sulfonic acid-based acrylic monomer, making it an excellent choice for preparing hydrogels designed for the removal of heavy metal ions from wastewater. AMPS-based hydrogels are classified as strong polyelectrolytes, exhibiting pH-independent swelling behavior, in contrast to their weak polyelectrolyte counterparts. The AMPS sulfonic acid group (R-SO_3_H) converts to a sulfonate group (R-SO^3−^) when the hydrogen atoms dissociate. Sulfonate (SO^3−^) functional groups within the polymer structure confer strong anionic characteristics that enhance the effective interaction and ion exchange with positively charged heavy metal ions. Studies have demonstrated that AMPS-based hydrogels achieve high removal efficiencies for various heavy metals due to the strong affinity of sulfonic acid groups for metal cations [124,125,126]. Shoueir et al. [127] synthesized core–shell nanogels through surfactant-free emulsion polymerization, creating a poly (vinyl alcohol) (PVA) core coated with a shell made of poly (2-acrylamido-2-methyl-1-propanesulfonic acid-co-N-isopropylacrylamide) (APMS). The results demonstrated that the synthesized core–shell nanogels are highly effective adsorbents for Pb^2+^ ions, achieving a maximum adsorption capacity of 510.2 mg/g. The electrostatic interaction between sulfonic acid groups and Pb^2+^ ions is responsible for a much higher adsorption trend, an excellent preconcentration factor, good precision, and rapid adsorption rates. The kinetic studies revealed that the adsorption process follows a pseudo-second-order model, while the adsorption isotherms are consistent with the Langmuir model, indicating a monolayer adsorption mechanism. Additionally, regeneration experiments showed a recovery efficiency ranging from 95.7–104.4 for Pb^2+^ ions in the environmental samples under the optimum conditions. These results were sustained over four cycles of sorption and desorption, demonstrating the nanogels’ potential for reuse and economic feasibility in practical applications.

The most essential requirements for a hydrogel in organic heavy metals retention are reusability, the reversibility adsorption process, and stability. Stability refers to the hydrogel’s ability to withstand typical environmental conditions. To achieve a stronger hydrogel structure, one commonly suggested approach in the literature is to create composites with inorganic constituents. For this purpose, inorganic materials such as graphene oxide (GO) [48,128], montmorillonite (MMT) [129,130], laponite (Lap) [131], carbon nanotubes (CNT) [132], and activated carbon (AC) [133] have been added as nanofiller reinforcements to enhance the stability, recyclability, and adsorption performance. For example, magnetic responsive composite hydrogel-based absorbents are materials composed of inorganic magnetic components embedded in an organic synthetic polymer. These composite hydrogels can effectively remove heavy metal ions, achieving impressive adsorption capacities of 289.12 mg Zn/g and 385.2 mg Cd/g [134]. Furthermore, hydrogels with encapsulated magnetic particles facilitate the magnetic separation of the adsorbate and adsorbent from the bulk reaction solution by using an external magnetic field [135].

By combining acrylic monomers and GO, a promising material was developed for the adsorption of heavy metal ions [136]. GO nanosheets consisting of oxygen-containing functional groups, such as hydroxyl and carboxyl, are linked to the surface of the acrylic hydrogel, while the gel structure is maintained. These nanocomposite hydrogels have effectively demonstrated reusability and high adsorption capacities for Cu^2+^, Pb^2+^, Zn^2+^, and Cd^2+^ ions in an aqueous solution that reached 1268 mg Cu/g_xerogel_, 2026 mg Pb/g_xerogel_, 704 mg Zn/g_xerogel_, and 632 mg Cd/g_xerogel_. After conducting three adsorption–desorption cycles, the acrylic/GO hydrogels demonstrated an efficiency of 88% or higher for all four tested metal ions. These findings suggest that acrylic/GO hydrogels are stable, efficient, low-cost, and reusable materials for removing metal ions from solutions [137].

**Figure 9:**
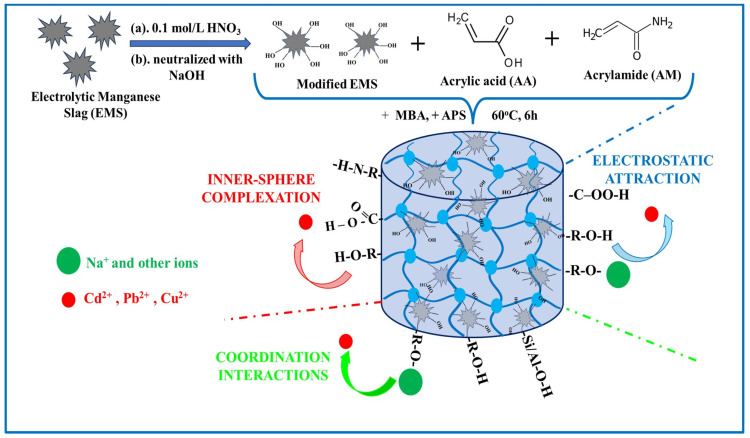
The synthesis process and schematic illustration of the adsorption mechanism for heavy metal ions onto the EMS-PAA-AM composite hydrogel (adapted from Ref. [137]).

Recently, electrolytic manganese slag (EMS), a solid waste of electrolytic manganese production, has been repurposed as a cost-effective adsorbent for heavy metal removal following appropriate modifications. Ma et al. [137] enhanced EMS via a one-pot method by activating it with NaOH and synthesizing an EMS-based double-network hydrogel (EMS/PAA hydrogel). This novel composite hydrogel exhibited good adsorption capacities for Pb^2+^ (153.85 mg/g), Cd^2+^ (113.63 mg/g), and Cu^2+^ (54.35 mg/g). The mechanisms for removing these three heavy metals using the new composite hydrogel involve several processes: an electrostatic attraction (between -COOH and -OH groups), inner-sphere complexation (with OH, -COOH, and -NH groups), and coordination interactions (involving Si-O and Al-O, as well as OH and -COOH groups) (see Figure 9). Therefore, ion exchange, hydrogen bonding, and hydrophobic and coordination interactions improve the development of innovative structures for synthetic polymer-based hydrogel adsorbents. Regeneration experiments demonstrated an excellent recyclability performance, exhibiting repeated reutilizations, excellent adsorption efficiency, and advantages such as a low cost, adjustable dimension, and easy separation of the composite hydrogels, making them ideal for practical applications.

## 3. Bio-Based Hydrogels Engineered to Capture Water Pollutants

**Figure 10:**
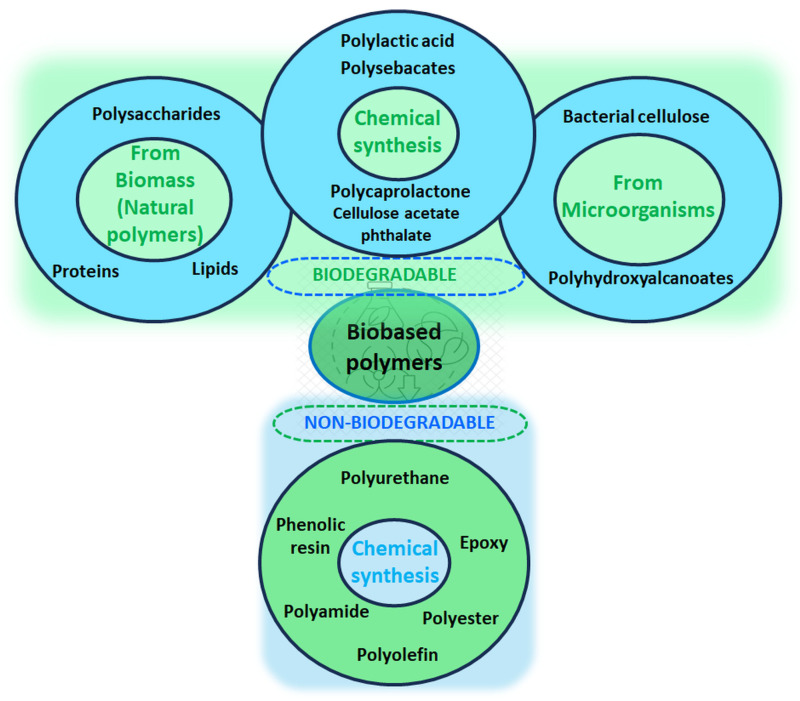
Schematic classification of bio-based polymers.

As industrialization continues to cause an unrelenting rise in water pollution, the demand for effective and sustainable wastewater treatment technologies has become increasingly critical. Nowadays, biobased polymers have attracted considerable research attention in many fields due to the tremendous increase in environmental pollution concerns and the severe worldwide depletion of crude oil reserves, and they have emerged as a powerful solution to replace conventional petroleum-based polymers. Bio-based polymers comprise at least one polymeric biomolecule generated from renewable feedstock, biological sources, or biodegradable materials (Figure 10) [140].

As it is well-known, polysaccharides are an important class of natural bio-based polymers. They originate from plants (cellulose, hemicellulose, starch, pectin, lignin, etc.), animals (chitin/chitosan, hyaluronic acid, etc.), algae (alginic acid, carrageenan, etc), and bacterial sources (pullulan, dextran, salecan, xanthan gum, etc.) [141,142]. Structurally, all polysaccharides consist of long chains comprising identical or different monosaccharide units bound together by glycosidic linkages. Despite all that, they differ in their position on the glycosidic skeleton and the types of functional groups. These dictate their interaction with surrounding molecules [142]. These are standard building blocks for the hydrogel’s preparation in various fields, especially in water treatment [143].

In addition, biopolymers possess economic and environmental advantages, such as a low price, abundance, biodegradability, biocompatibility, abundant hydrophilic functional groups in their structures, and antimicrobial properties [144], which are desired features in many applications, including water treatment applications, wherein pollutants’ contamination can cause serious problems for public health. In addition, the inherent properties of biopolymers contribute to sustainability by reducing the waste impact of oil-based polymers. The most significant benefits of biobased polymers refer to environmentally friendly perspectives, good strength, and reliability according to the demand. We have been relying on synthetic materials, which are the major contributors to water pollution. This pollution is very hazardous and toxic for the environment and, consequently, for our healthcare.

**Figure 11:**
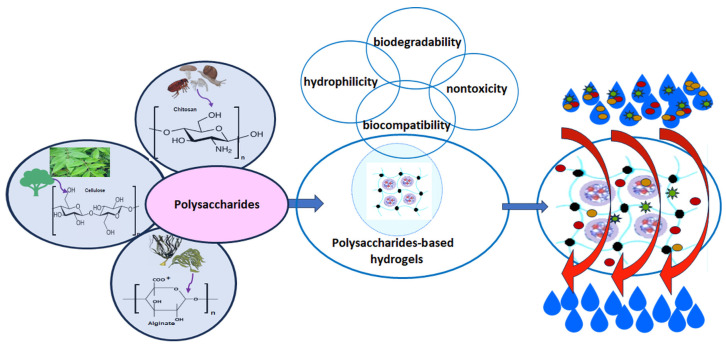
Polysaccharide-based hydrogels for application in water treatment.

Recently, natural biobased hydrogels for water purification have become increasingly attractive to researchers compared to synthetic hydrogels due to their distinctive physico-chemical properties, such as hydrophilicity, biodegradability, biocompatibility, and nontoxicity. These hydrogels not only exhibit unique physical and chemical properties conducive to pollutant capture, but also align with the principles of sustainability. Green renewable hydrogels derived from polysaccharide-based biopolymers (i.e., cellulose, chitosan, and alginate) and their derivatives are three-dimensional polymer networks made of natural, nontoxic, and renewable materials that swell in aqueous solutions due to electrostatic repulsions between their hydrophilic functional groups [145,146,147]. The hydrogel structure swelling exposes their surface functional groups, which also enhances the removal of a wide range of contaminants such as heavy metal ions [148,149], toxic dyes [150,151], pesticides [152], etc., (Figure 11).

Therefore, with the relentless increase in water pollution owing to industrialization, this chapter emphasizes the great potential of long-lasting polysaccharide-based hydrogels, specifically cellulose, chitosan, and alginate, the most widespread biopolymers, to address this challenge and to facilitate sustainable wastewater purification technologies concerning significant water pollutants, such as heavy metals’ retention and dye removal. Since the literature regarding natural and bio-based polymeric materials is significantly broad, we focus mainly on the most recent and intriguing advancements in designing natural polymer-based hydrogels for water treatment, highlighting their synthesis method and performance in removing various contaminants.

### 3.1. Polysaccharides-Based Hydrogels for Heavy Metals’ Retention

Water contamination with heavy metals is of great concern. Polysaccharide-based hydrogels were recently paid a great deal of interest for this ecological issue, as they can attain high retention rates and may be applied in various environments. Their effectiveness relies on several properties that influence how they interact with metal ions, including their molecular weight, hydrophilic–hydrophobic balance, functional groups, crosslinking degree, surface area, and pore volume. These properties influence the hydrogel’s ability to bind metal ions while preventing damage to the structural integrity and stability in water. The molecular weight and crosslinking degree impact the mechanical strength and swelling behavior, while functional groups, such as -OH, -COOH, -NH_2_, and -SO_3_H, may be involved in ion binding, and thus the removal efficiency is enhanced. Hydrophilic interactions are beneficial for the swelling process, while hydrophobic interactions assist in addressing organic contaminants. A larger surface area and higher pore volume improve the hydrogel’s adsorption and uptake capacity, especially in highly contaminated environments.

#### 3.1.1. Cellulose-Based Hydrogels for Heavy Metals’ Retention

In this context, cellulose and its derivatives are some of the most abundant natural biopolymers, and significant progress has been made towards their applications. Cellulose is a linear syndiotactic, semi-crystalline, and high-molecular-weight homopolymer of β-D-glucopyranose units linked by β-1,4-linkages, where cellobiose is the repetitive unit dimer of glucose. The hydroxyl groups generate intra- and inter-hydroxyl hydrogen bonds, resulting in a supramolecular polymer with ordered crystalline sections [153]. Cellulose’s hydroxyl groups can be modified by surface modifications to confer greater chemical activity and, thus, improve its adsorption properties. Related to this, cellulose (i.e., native/pure cellulose and bacterial cellulose), its derivatives (ether derivatives: methylcellulose, ethylcellulose, hydroxyethyl methylcellulose, hydroxypropyl cellulose, carboxymethyl cellulose, etc.; ester derivatives: acetate phthalate, acetate trimellitate, acetate succinate, hydroxypropyl methyl phthalate, hydroxypropyl methyl phthalate, etc.), and nanocellulose-based hydrogels have been significantly exploited for environmental applications [154]. The presence of various surface functional groups able to coordinate heavy metals, together with the possibility of generating interconnected porous structures, has encouraged cellulose application for hydrogels’ development in water treatment against heavy metals [155,156].

In the hydrogel form, cellulose’s hydrophilicity can be increased to make the aqueous sorption process more efficient [157]. Several works have revealed that the addition of solid particles to the hydrogel as supporting frameworks can enhance the adsorbate’s interaction with the adsorbent’s active sites [158]. For instance, Lunardi and co-workers [159] prepared hydrogel composites based on cellulose from rice husk waste and an activated zeolite to remove copper ions from water. Adsorbent preparation was performed by crosslinking the gel rice husk cellulose, dissolved in NaOH/urea, incorporating a mesoporous zeolite in a ratio of cellulose-to-zeolite of 4:2. The adsorption kinetics revealed that the zeolite addition leads to a more rapid adsorption of Cu^2+^; based on the pseudo-first-order parameter, the rate increased from 2 × 10^−3^ to 7 × 10^−3^ min^−1^, and the adsorption equilibrium was reduced from 20 h to 8 h. However, incorporating zeolite contrarily affected the adsorption capacity, which decreased from 17.05 to 10.77 mg g^−1^ for the adsorption process at 30 °C.

However, blending cellulose with another biopolymer can substantially improve the adsorption and mechanical properties thereof. This not only introduces functionality for metal adsorption, but can also increase the specific surface area and enhance the mechanical strength of the composite hydrogel, compared to a neat cellulose hydrogel. In this context, the group of Yang [160] fabricated cellulose–chitosan composite hydrogels through a co-dissolution and regeneration technique using a LiBr solution as a solvent. The effects of the pH, contact time, and the initial concentration of the metal ion solution on Cu^2+^ adsorption were investigated. Moreover, the impact of other competing metal ions (Zn^2+^ and Co^2+^) was assessed. Batch adsorption experiments showed that the composite hydrogel exhibited 10 times greater than the adsorption capacity toward Cu^2+^ of neat cellulose hydrogel, with a 94.3 mg/g value. Competitive adsorption from a solution containing mixed metals revealed that the composite hydrogel exhibited the selective adsorption of the metals in the following decreasing order: Cu^2+^ > Zn^2+^ > Co^2+^.

Furthermore, in a recent study [161], an environmentally friendly and multicomponent composite hydrogel for the removal of copper was developed through a one-step crosslinking and encapsulation method of microcrystalline cellulose, chitosan, magnetite, and alginate (CCMA). This method was employed to overcome the multiple synthesis methods of previous studies. The CCMA’s removal capacity was tested in a Cu solution with a pH variation between 3 and 6. The combination of the three biopolymers had the expected effect, revealing that the best Cu^2+^ removal by CCMA was achieved at pH 6 with a yield of 91.29%.

#### 3.1.2. Chitosan-Based Hydrogels for Heavy Metals’ Retention

Another important biopolymer of particular interest in the development of hydrogels is chitosan. Chitosan is a linear polysaccharide derived from naturally occurring chitin, sourced primarily from crustaceans, insects, and fungi, which is, after cellulose, the most common biopolymer on earth [162]. Chitosan is a partially or entirely N-deacetylated derivative of chitin, chemically composed of D-glucosamine and N-acetylglucosamine subunits joined through β(1-4)glycosidic bonds, which has primary amino groups that provide a positive charge under an acidic pH [163]. Due to their surface chemical composition, which can include functional hydroxyl and amino groups that function as binding sites, chitosan can readily adsorb various contaminants through hydrogen bonds and electrostatic interactions, and can also provide the possibility of modification.

However, the use of neat chitosan is inadequate because of its low elasticity, poor mechanical strength, and poor solubility, which is why crosslinking, grafting, fillers’ incorporation, interpenetrating, impregnating, and blending procedures have been widely investigated to improve the mechanical properties and adsorption capacities of chitosan-based hydrogel [164,165,166,167,168,169,170,171,172,173,174]. Thus, combining chitosan with other components has made way for improvements in terms of chemical resistance, mechanical and thermal stability, low costs, and reuse [175]. Table 4 represents a comparative study of the adsorption performance of chitosan-based hydrogels for removing different heavy metals. 

Remarkably, chitosan exhibits an increased number of binding sites for metal ions when crosslinked with glyoxal, epichlorohydrin, glutaraldehyde, ethylene glycol, diglycidyl ether, formaldehyde, isocyanates, carbodiimides, and polyvalent cations. Given the circumstances, a blend of crosslinked chitosan would be ideal for overcoming the drawbacks of poor mechanical stability. In this respect, studies have been conducted to develop double-network hydrogels, which are hydrogels based on two blended biopolymers (e.g., chitosan and alginate can be blended by the interaction between the amino group of chitosan and carboxyl group of alginates to form chitosan–alginate hydrogel beads) or crosslinked polymer networks, to increase the mechanical strength of hydrogels [164,165,166,168,169,170,171,172].

Physical hydrogels are also extremely attractive as they are crosslinked by non-covalent interactions without the use of additional crosslinking agents [176]. Therefore, the development of fully physically crosslinked double-network hydrogels is highly desired. In this context, Tang et al. prepared, for the first time, a chitosan–sodium alginate–calcium ion physically crosslinked double-network hydrogel (CTS/SA/Ca^2+^ PCDNH) by combining the internal gelation method with the semi-dissolution acidification sol–gel transition method, for the removal of Pb^2+^, Cu^2+^, and Cd^2+^. The two physical networks were formed simultaneously and independently [173]. The obtained hydrogel exhibited excellent mechanical characteristics, with a maximum tensile strength of 0.190 MPa. The single-network physical hydrogel is crosslinked by electrostatic interactions, which overcomes the weak hydrogel mechanical properties. In addition, Pb^2+^, Cu^2+^, and Cd^2+^ retention efficiencies were also adequate, reaching up to 176.50 mg/g, 70.83 mg/g, and 81.25 mg/g, respectively.

Magnetic separation is an efficient low-cost method of separating magnetic adsorbents from aqueous systems by applying an external magnetic field [177]. Magnetic adsorbents are usually created by associating with Fe_3_O_4_ [167,171] or magnetic bentonite [168], which can be blended to form the hydrogel beads for easier separation from the solution. From these studies, the removal efficiency for Pb^2+^ was 99.00% and for Cu^2+^ varied from 51% to 93.00%.

Most cited references from Table 4 dealt with materials in the form of beads obtained by crosslinking. However, Perumal et al. [174] performed fascinating work, developing spherical hydrogel particles (CG) using different combinations of chitosan and gelatine biopolymers by an inverse polymerization method. In the single metal ion solution, CG hydrogels showed higher adsorption for the Hg^2+^ ion (>50%), while the other metal ions showed lower removal efficiencies (<10%). In the multiple metal ion solution, CG hydrogels presented high removal efficiencies for all metal ions (54–95%). This work also suggests that oven drying is more efficient and convenient in heavy metal ion adsorption than freeze-drying, and it can be helpful for large-scale hydrogel production for the adsorption of multiple metal ions.

#### 3.1.3. Alginate-Based Hydrogels for Heavy Metals’ Retention

Alginate is a natural, biocompatible, non-toxic, biodegradable, and intensively studied linear anionic polysaccharide that can be extracted from marine algae containing β-D-mannuronate and α-L-guluronate residues linked by (1,4)-glycosidic bonds. Alginate contains different percentages of these compounds depending on the extraction source, influencing the material properties [178]. It bears many internal hydroxyl and carboxyl groups, which can be crosslinked with divalent metal ions to form a three-dimensional structure. However, the low physical strength, poor thermal stability, and degradation of sodium–calcium alginate greatly limit its application in water treatment; therefore, physical, or chemical modifications are needed to improve its further applicability in adsorption [179]. Thereby, alginate can be modified through oxidation, hydrophobic modification, esterification, and grafting copolymerization, with cellulose, chitosan, and metal–organic frameworks to improve the adsorption capacity for metal removal [180].

To overcome the above limitations, physical blending with other biopolymers and chemical modification with a crosslinker can be considered an appropriate technique [181]. Polymer blends refer to the physical mixing of two or more polymers to incorporate their best properties, enhanced mechanical characteristics, and the effective removal of heavy metals from wastewater [182]. For instance, Ren and co-workers [179] prepared sodium alginate (SA)–carboxymethyl cellulose (CMC) gel beads for Pb^2+^ ions elimination through blending and crosslinking. The physical, chemical, and electrostatic Pb^2+^ adsorptions in the SA–CMC removal mechanisms were debated based on the adsorption kinetics. The Pb^2+^ adsorption mechanisms of the hydrogel beads based on the (i) physical adsorption: SA modified by CMC crosslinking (the crosslinking degree, based on the swelling capacity, was 1.73 g/g) acquired internal pore structures and a specific surface area, providing more adsorption sites to Pb^2+^; (ii) chemical adsorption: given that O in −COO− and −OH has a lone pair electron and Pb^2+^ provides an unoccupied orbital, the functional groups in the SA–CMC gel beads could complexate Pb^2+^; (iii) electrostatic adsorption: the gel beads’ surface is negatively charged, but Pb^2+^ is a positively charged metal cation. The Pb^2+^ adsorption of SA–CMC gel beads exceeded 99%, showing that the removal efficiency was significantly higher than that of conventional adsorbents.

Further on, the work of Song et al. [183] provides some insights into Ni^2+^ removal by crosslinked SA-carboxymethyl chitosan hydrogel beads (CHB) which were synthesized using calcium chloride and epichlorohydrin as the crosslinkers. Several critical parameters influencing the adsorption of Ni^2+^ ions, such as the pH, contact time, initial Ni^2+^ concentration, and regeneration efficiency were investigated. The results revealed that the CHB performance for Ni^2+^ was 128.4 mg/g. The equilibrium adsorption data fitted the Freundlich model and the adsorption kinetic data followed the pseudo-second-order model. Moreover, the prepared CHB presented good adsorption efficiency after five regeneration cycles.

Recently, magnetic adsorbents have been used for the adsorption of metal ions in aqueous solutions [169,184]. These adsorbents exhibit higher adsorption activity along with their specific magnetic properties, which enables easier collection and separation procedures from aqueous media. An interesting study on Cu^2+^ removal is provided by Yu et al. [185], which described a magnetic composite gel bead, calcium alginate (CA)/carboxymethyl cellulose (CMC)@MnFe_2_O_4_, prepared using CA- and CMC-encapsulated MnFe_2_O_4_ through blending and crosslinking. Several adsorption conditions were tested, including the initial Cu^2+^ concentration, solution pH, and equilibrium contact time. The adsorption data follow the pseudo-second-order model and fit better to the Langmuir isotherm, suggesting a monolayer adsorption process with chemisorption as the rate-limiting step. The gels registered adsorbed Cu^2+^ with a maximum capacity of 77.22 mg g^−1^. From adsorption data fitting, Cu^2+^’s removal mechanisms of CA/CMC@MnFe_2_O_4_ follow an ion exchange as well as the formation of complexes.

**Figure 12:**
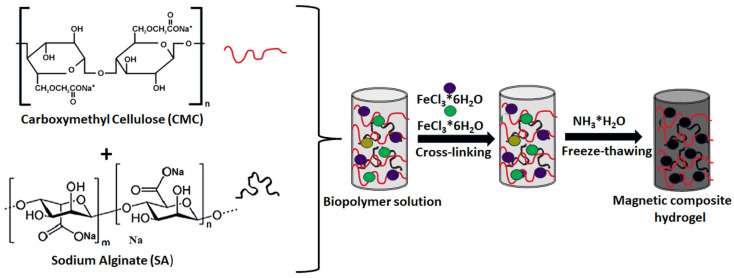
Preparation diagram of a magnetic composite hydrogel. Adapted from [186].

Magnetic polysaccharide composite hydrogels were also synthesized as schematically described in Figure 12 by another research group [186], based on SA and CMC as the backbone and filled with in situ Fe_3_O_4_ nanoparticles by a green method, implicating co-precipitation and a subsequent freeze–thawing method to treat wastewater containing Cu^2+^, Pb^2+^, and Mn^2+^. The effect of the pH, contact time, and the adsorbent dosage’s effect on the adsorption of heavy metal ions by the magnetic SA/CMC hydrogel has been studied. From the Langmuir model, the maximum adsorption capacity for the Mn^2+^, Pb^2+^, and Cu^2+^ ions were 71.83, 89.49, and 105.93 mg/g, respectively. The heavy metal adsorption was attributed to chemical adsorption and ion exchange processes.

It is noteworthy that the design of Amidoxime (AO)-based adsorbents is still in progress for improving the selectivity for U^6+^ in aqueous solutions [187]. To effectively bind the uranyl ion (UO_2_^2+^), the ligand structure should contain a functional amidoxime group consisting of an oxime and an amino functionality. In this respect, many methods have been investigated to enhance AO’s adsorption capacity. For instance, adding auxiliary groups like amino or carboxyl to hydrogels allowed AO-functionalized adsorbents to modify their uranium coordination environment and increase their affinity and selectivity for UO_2_^2+^. In a recent work, the group of Ji et al. [188] proposed the preparation of a nano-ZnO-enhanced amidoxime-functional sodium alginate composite hydrogel (SA/CMC-ZnONPS-AO1), adopting both the co-mingling and covalent crosslinking methods. The introduction of the amidoxime group effectively improved the selectivity of uranyl ions (Kd_U_ = 10,016 mL/g) and the maximum adsorption efficiency of hydrogels for UO_2_^2+^ was higher than the sodium alginate-based adsorbents currently used, with a value of 641.7 mg/g.

### 3.2. Polysaccharides-Based Hydrogels for Dyes’ Removal

Color contamination from dyes, including anionic, cationic, and non-ionic dyes, poses a significant threat to water quality [189]. There is a wide range of adsorbed dyes, among which Congo Red (CR), Methyl Orange (MO), and Methylene Blue (MB) are the most widespread. There are many methods for eliminating dyes from wastewater, which are costly to implement and can cause secondary pollution by generating harmful byproducts [190]. However, the hydrogel’s adsorption process is simple, affordable, and efficient. Polysaccharide-based hydrogels present a sustainable approach to dye removal from wastewater. Their tunable properties allow customization based on specific dye characteristics, making them a practical and eco-friendly solution for environmental remediation.

Table 5 summarizes the polysaccharide-based hydrogels used in the last years for dye adsorption applications. The most relevant systems that will be described hereafter refer to natural materials and hydrogels in which case adsorption performances are compared.

#### 3.2.1. Cellulose-Based Hydrogels for Dyes’ Removal

Cellulose-based hydrogels have been used in recent years to remove anionic dyes such as CR [191], MO [192], Acid Orange II (AO II) [193], and Trypan Blue (TB) [194], and cationic dyes such as MB [193,195] and Crystal Violet (CV) [194] from water solutions. In this regard, cellulose and chitosan can be combined to create new hydrogels with excellent mechanical properties and efficient dye adsorption, superior to the constituent biopolymers. Li et al. [191] reported obtaining cellulose–chitosan hydrogel beads by extruding and regenerating the components from ionic liquid 1-ethyl-3-methylimidazolium acetate in ethanol. The beads had a more efficient adsorption capacity than the most reported natural biosorbents, with retention efficiency for CR from aqueous solutions of 89.6%.

**Figure 13:**
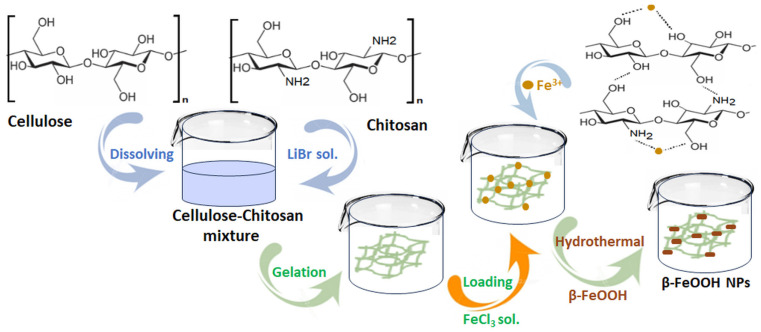
Cellulose–chitosan/β-FeOOH composite hydrogels synthesis route. Adapted from [192].

Yang and co-workers [192] prepared -FeOOH cellulose-chitosan/β-FeOOH composite hydrogels via co-dissolution and regeneration synthesis and a hydrothermal in situ process depicted in Figure 13. The addition of β-FeOOH nanoparticles was found to act as an efficient photocatalyst within the process of MO degradation. A 60% removal efficiency was recorded, which may be attributed to the orientation of the metal oxide nanoparticles, which fill the framework’s porous structure, thus hindering the anionic dye’s interaction with the hydrogel.

Cellulose contains many hydroxyl groups, but due to its participation in the hydrogen bonds’ formation, cellulose loses its water adsorption capacity. For this reason, (CMC) becomes a better option, which can be prepared by attaching hydrophilic groups (such as carboxyl groups) through an etherification process, leading to an enhanced adsorption capacity for dyes. In this direction, Wang et al. [193] created IPNs of CMC and chitosan by crosslinking with epichlorohydrin to adsorb different dyes from water. Even after five adsorption–desorption cycles, the new hydrogel’s removal efficiency for Acid Orange II was over 90%, and for MB, it was 95%. In a different study, A. Meas et al. [194] created carboxymethyl cellulose from wood sawdust. They combined it with sodium alginate microgels using a microfluidic technique to crosslink calcium chloride. After five cycles, adding CMC to the SA microgel improved the adsorption–desorption efficiencies, thus attaining 96.44% and 95.41% for TB and CV, respectively.

An interesting study [196] describes a green Sugar Beet Pulp Cellulose/Starch/Activated Carbon-ZnO hydrogel prepared by crosslinking and ultrasonic cavitation. The adsorbent was tested for MB and MO removal. The process better aligned with the pseudo-second-order kinetic model and the Langmuir isothermal adsorption model. The adsorption results showed that the hydrogels had an excellent adsorption performance of 91.22% or 90.44%, respectively, after five adsorption cycles.

#### 3.2.2. Chitosan-Based Hydrogels for Dyes’ Removal

Due to its excellent adsorption capabilities, chitosan has been widely studied as an adsorbent for dye removal from aqueous solutions. However, chitosan is prone to dissolve in acidic conditions which significantly limits its use. Therefore, the chemical crosslinking of chitosan emerges as a viable means of enhancing its chemical and mechanical strength. Additionally, the literature studies propose using IPNs to yield an interlocked structure within the crosslinked networks, with implicit performance benefits. One attempt was made by Ngwabebhoh et al. [197], which reported a study on the efficient removal of Direct Red 80 (DR80) dye from aqueous solutions using a semi-IPN chitosan-starch hydrogel. The researchers examined the kinetics, isotherms, and thermodynamics to describe the sorption process. The results strongly correlated with the Freundlich isotherm, and the pseudo-second-order model best described the sorption kinetics. The hydrogel achieved an adsorption of dye with a maximum capacity of 330,86 mg/g and a retention efficiency of 84.2%. In another study [198], IPN chitosan–gelatin porous materials were synthesized by freeze-drying and tested as adsorbents for removing the anionic dye Acid Orange II from aqueous solutions. Genipin, a natural crosslinker, was used to form the IPN structure. The experiments were conducted under different conditions, varying genipin concentrations, adsorption times, and pH values. The results indicated that the material exhibited the highest adsorption capacity of 573 mg/g when the genipin concentration was 0.25 mmol/L. Furthermore, the adsorption capacity increased as the solution pH decreased.

In addition to modifying chitosan-based adsorbents through physical or chemical crosslinking, chitosan-based hydrogels’ mechanical strength and dye adsorption capacity can be enhanced by creating chitosan-based composites. This is achieved by incorporating fillers such as halloysite nanotubes [199], nano zinc oxide [200], activated carbon [201], Fe magnetic nanoparticles [202], hematite (α-Fe_2_O_3_) nanoparticles [203], phytic acid [204], bentonite [205], calcium [206], graphene oxide [207], or rectorite [208], which are responsible for strong interactions between chitosan and (nano) particles. For example, the adsorption performance and retention capacity of chitosan hydrogel beads were improved by the incorporation of halloysite nanotubes by the dropping and pH-precipitation method for the removal of MB and Malachite Green (MG) dyes [199]. The results achieved were 270.27 mg/g and 95.2% for MB, and 303.03 mg/g and 97.5% for MG. Bentonite clay nanoparticles were impregnated into chitosan-g-gelatin nanocomposite hydrogels to develop adsorbents for the CR dye [195]. Under optimal conditions, the maximum dye removal and adsorption capacity reached 93.85% and 453.87 mg/g, respectively, showcasing improved reusability across five successive cycles. Graphene oxide (GO) was added into the crosslinked chitosan and CMC nanocomposite hydrogels for the removal of both cationic (MB) and anionic (MO) dyes [197]. Initially, GO was functionalized with vinyltriethoxysilane and used as a chemical crosslinker to synthesize the nanohydrogels with a polymeric mixture of diallyldimethylammonium chloride and 2-acrylamido-2-methyl-1-propanesulfonic acid. The nanohydrogels exhibited an impressive adsorption of MB and MO with maximum adsorption capacities of 655.98 mg/g for MB and 404.52 mg/g, respectively, along with an excellent regeneration capacity over twenty continuous adsorption–desorption cycles.

#### 3.2.3. Alginate-Based Hydrogels for Dyes’ Removal

Alginate contains negatively charged carboxylate functional groups, which endow it with a high affinity and binding capacity for dyes. Some studies have used a sodium alginate hydrogel as a substrate, combined with other biopolymers [209] or adsorption materials, such as bentonite [210] or graphene oxide [211], to enhance the adsorption effect of the hydrogel on different dyes, which exhibited a relatively high adsorption capacity compared to some adsorbents.

For instance, Wang et al. [209] developed an innovative green hydrogel by combining aminated lignin and sodium alginate. This was achieved through the chemical crosslinking of aminated lignin with epichlorohydrin and ionic crosslinking of alginate with Ca^2+^. The hydrogel’s performance in adsorbing MB was evaluated and key factors such as the adsorbent dosage, initial dye concentration, pH, and temperature were examined for their impact on adsorption. The hydrogel exhibited a maximum adsorption capacity of 388.81 mg/g, as determined by the Freundlich isotherm, significantly higher than other biomass-based adsorbents reported in the literature. Additionally, the hydrogel demonstrated good recyclability, with a retention efficiency of 87.64% after five cycles.

Encapsulating clay or carbon-based nanomaterial particles within a polysaccharide polymer matrix presents a promising and cost-effective approach to developing materials with high sorption capacities for organic compounds and cationic species. For instance, Belhouchat et al. [210] fabricated crosslinked activated organo-bentonite/sodium alginate composite beads by the crosslinking of sodium alginate with hydrochloric acid and incorporating activated organo-bentonite into the sodium alginate hydrogel. These composite beads removed MB and MO from aqueous solutions. The effects of the solution pH, adsorption kinetics, and isotherms on the adsorbent’s capacity were determined by approaching batch adsorption experiments. The adsorption kinetics for both MB and MO followed a pseudo-second-order model, while the Langmuir model best fitted the isotherms. The adsorption capacities ranged from 183 to 1309 mg/g for MB and 7.13 to 141.27 mg/g for MO. Another study by Gan et al. [211] explored a composite hydrogel made from graphene oxide (GO) and sodium alginate for the removal of MB, RhB, Vat Green 1 (VG1), and MO. The incorporation of GO significantly improved the adsorption capacity of the SA/GO beads for a range of organic dyes, with all adsorption capacities exceeding 200 mg/g.

## 4. Hybrid Hydrogels for Water Treatment

**Figure 14:**
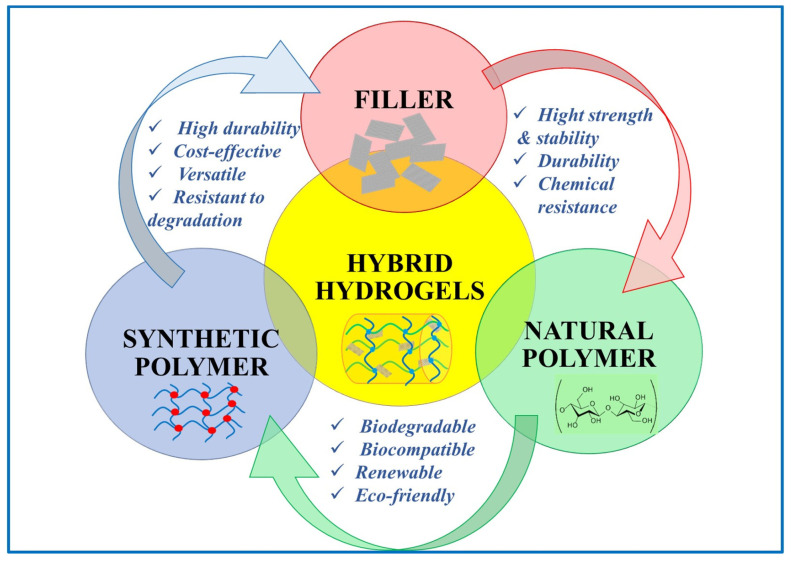
Individual benefits of the main constituents involved in obtaining hybrid hydrogels.

The third main category of hydrogels used for water treatment consists of hybrid hydrogels, which contain a synthetic part and a natural part in their structure. These hydrogels are used for the removal of organic pollutants, such as dyes, and inorganic pollutants, such as heavy metal ions. Dyes, especially industrial ones, and heavy metals represent two significant classes of pollutants for water bodies. This section deals with absorbent hydrogel materials based on natural polymer–synthetic polymer mixtures, with or without a filler material, used to remove dyes and heavy metals. The greater preference for using natural polymers (biopolymers) as hydrogel precursors over their synthetic counterparts shows certain benefits regarding biocompatibility and biodegradability, but also some drawbacks. Biopolymers in the natural state were proven to lead to poor mechanical features of the hydrogels and, therefore, challenges related to their repeated use in adsorption. However, modifying biopolymers with different chemical compounds may surpass this inconvenience. There are various approaches to modifying biopolymers. One viable means is to mix them with synthetic polymers, which may be carried out by different methods, including graft copolymerization [212,213,214,215]. As shown in Figure 14, each of the components involved in the development of hybrid hydrogels brings specific benefits.

### 4.1. Hybrid Hydrogels for Dye Removal

The presentation of information in the literature begins with studies that deal with dyes. Some examples of industrial dyes reported to be removed by hydrogel-based systems are Methylene Blue (MB) [212,213,216,217,218,219,220,221,222,223,224,225,226,227,228,229,230,231,232]; Methyl Violet (MV) [212]; Brilliant Green (BG) [233]; Rhodamine B (RB) [218,219,220,233,234,235]; Methyl Orange (MO) [215,219,220,236]; Congo Red (CR) [213,226,233,237]; Malachite Green (MG) [219,235,238,239,240]; Indigo Carmine (IC) [217,241]; Rhodamine 6G [241]; Sunset Yellow-SY [219,241]; Crystal Violet (CV) [219,230,242,243,244,245]; Mordant Blue 9 [218]; Fluorescein sodium Salt (FS) [219]; Orange II sodium Salt (O II) [219]; basic yellow [246]; reactive blue 2 [223]; Safranin T (ST) [247]; Brilliant Cresyl Blue (BCB) [247]; and Bromophenol Blue (BPB) [230]. The systems used to develop hydrogels for removing these dyes are analyzed, including the precursors used (natural polymer–synthetic polymer–filler) and the results regarding the removal process. The information is presented with the natural polymers used as the criterion. Three main classes of natural polymers were considered, so three subsections appear: polysaccharides, gum polymers, and other polymers.

#### 4.1.1. Hybrid Hydrogels Based on Polysaccharides for Dye Removal

Polysaccharides exhibit certain advantages related to their renewability and biocompatibility, which makes them suitable for water treatment. The most relevant results related to hybrid hydrogels based on polysaccharides and their performances in dye removal are discussed for the following polysaccharides: cellulose [218,220,228,230,231,246,247,248], alginate [213,226,232,239,244,249], chitosan [219,241,243,250,251], salecan [227], pullulan [223,244], salep [236], and starch [222,225,229].

**Figure 15:**
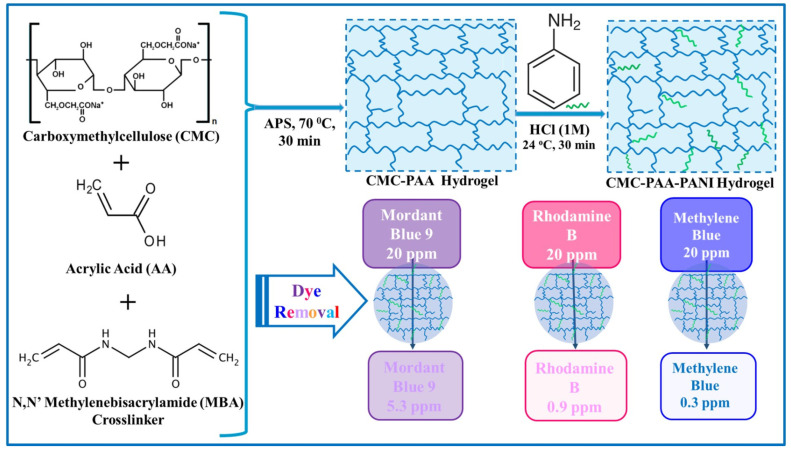
The synthesis process and schematic illustration of dye removal of macro-porous CMC-PAA-PANI network hydrogel (adapted from Ref. [218]).

Several studies [218,220,228,230,231] used cellulose or cellulose derivatives as the natural polymer component to develop systems that remove the MB dye. Bagheri et al. developed a hybrid system using a derivative of the cellulose natural polymer, i.e., carboxymethyl cellulose (CMC), in conjunction with a synthetic polymer, polyacrylic acid (PAA), and an electroconductive polymer polyaniline (PANI). The gel structure was provided by crosslinking means using N,N’-methylenebisacrylamide. The resulting CMC-PAA-PANI hydrogel showed dye removal features (three dyes were tested: mordant blue 9, RB, and MB). Moreover, the developed material was shown to possess antibacterial activity. Figure 15 summarizes the stages of developing the targeted hybrid hydrogel (CMC-PAA-PANI) and its performance in dye removal. Even though 20 ppm were submitted to removal using the elaborated hydrogel for each of the three dyes, the efficiency was different for each. The dye removal performance varied in the following order: MB (5.3 ppm) > RB (0.9 ppm) > mordant blue 9 (0.3 ppm) [218].

Huang and co-workers [220] elaborated injectable hydrogels using hydrazine-modified poly(N-isopropylacrylamide) (PNIPAm) and aldehyde-modified CMC as precursors, whereas graphene oxide was employed as a nanofiller. The developed system successfully removed MB dye.

Another study [228] also dealt with hydrogels based on CMC for MB removal. This time, hydrogels with IPNs were elaborated using CMC and crosslinked PAA (cPAA). Four CMC: cPAA mass ratios were investigated: 100:0, 25:75, 50:50, 75:25, and 0:100, respectively. The hydrogel prepared at a ratio of 50:50 was found to be particularly efficient for removing dyes (MB) and heavy metals (Cu). The hydrogel prepared at a ratio of 50:50 showed the most promising results in terms of MB removal.

Getya et al. [230] used natural cellulose along with poly(ethylene glycol) (PEG) to develop hydrogels with dye-removal features. PEG was crosslinked with cellulose macromonomers, and the hydrogels thus produced were used to remove MB, CV, and BMB.

Liu et al. used [231] cellulose recovered from tea residue and GO to remove MB. The as-synthesized hydrogels were denoted TCH-GO (tea cellulose hydrogel–graphene oxide) followed by the amount of GO (%). Therefore, five hybrid samples were prepared (TCH-GO0.5; TCH-GO1; TCH-GO2; TCH-GO5; and TCH-GO10) and a reference sample without GO was denoted TCH. According to the pseudo-second-order model, good removal efficiency was found and chemisorption occurred. The best MB removal performance, 92.7%, was recorded for the hydrogel TCH-GO10.

Aside from MB, cellulose-based hybrid hydrogels proved efficient in promoting the removal of other dyes, as follows: basic yellow 28 [246], ST, and BCB [247], as well as other anionic dyes (AR13, AB92, and AR112). Ullah et al. copolymerized natural and synthetic polymers, i.e., hydroxyl ethyl cellulose (HEC) and PAA, to develop a grafted copolymer efficient for removing basic yellow 28 [246]. Table 6 summarizes the most recent breakthroughs related to dye removal using hybrid composite hydrogels.

In the work of Mandal [247], a comparative study was carried out using different acrylic polymers as synthetic components for modifying the natural precursor, i.e., CMC. Three acrylic systems were investigated as follows: (1) AA/MBA, (2) sodium acrylate (SPA)/MBA, and (3) comonomers AA/hydroxyethyl methacrylate (HEMA)/MBA. Although all three systems showed high removal efficiencies towards ST and BCB, SPAMC was superior. The dye adsorption was endothermic, and ST was less adsorbed than BCB.

By approaching crosslinking, Kono and co-workers [250] elaborated on cationic hydrogels composed of cellulose and PEG moieties. For this purpose, quaternized cellulose with different degrees of substitution was subjected to crosslinking with poly(ethylene glycol) diglycidyl ether (PEGDE). The quaternization agent in this study was 2,3-epoxypropyltrimethyl ammonium chloride. Therefore, three cationic cellulose hydrogels (CCGS), denoted (CCG1–CCG3), with different degrees of substitution (0.56, 0.84, and 1.33, respectively) were prepared. Comparative studies on removing several dyes were also retrieved. These hybrid hydrogels showed adsorptive features towards anionic dyes (AR13, AB92, and AR112). A pseudo-second-order kinetic was identified for adsorption, whereas the Langmuir model fitted the equilibrium isotherm.

Alginate and its derivatives were also helpful in developing hybrid hydrogels to remove heavy metals [213,226,232].

Zhang [213] and Berg [226] previously reported that the systems used for MB, the SA-maleic anhydride–acrylamide copolymer, and calcium alginate-PEI can also degrade CR.

Zhang et al. [213] used sodium alginate (SA) with a maleic anhydride–acrylamide copolymer. Thus, the copolymer was first synthesized and integrated within the SA biopolymer, followed by crosslinking in two stages, with calcium chloride and glutaraldehyde. This system can be applied to remove of both dyes and heavy metals, with a better performance for the latter.

Berg et al. [226] elaborated on composite bead hydrogels for MB and CR removal, using calcium alginate as a natural component and polyethyleneimine (PEI) as a synthetic component. PEI played a modifier role. For a complete study, a reference adsorbent hydrogel was prepared using only alginate without being modified with PEI. A somewhat different adsorptive behavior was found for the two investigated dyes since alginate hydrogels absorbed MB at a rate of 2.3 × 10^–4^ mol/g. In contrast, CR was absorbed by the composite hydrogels at a rate of 6.69 × 10^–5^ mol/g.

Tao et al. [232] elaborated an innovative three-component system in the form of aerogel beads that can remove MB. In the first stage, a composite of Graphene Oxide (GO) and montmorillonite (MMT) was prepared, followed by aerogel preparation after adding SA to the system. MB was the best absorbed of the three tested dyes (MB, RB, and MO). The adsorption mechanism was governed by chemical and multilayer adsorption.

In addition, Aljeboree et al. reported a more complex composition containing SA-g-poly(itaconic acid-co sodium 4-vinyl benzene sulfonate)/Ricinus communis. Ricinus communis extract was used to yield activated carbon. This system was tested for its dye removal efficiency using MG as a cationic dye model. On this occasion, it was found that the developed system demonstrated a strong adsorption capability regardless of the initial dye concentration, pH of the solution, and process temperature. Moreover, four adsorption–desorption cycles may be carried out. The maximum removal efficiency of 96.33% was attained at pH 7. The highest adsorption efficiency of 1339.83 mg/g corresponded to a temperature of 25 °C [239].

Ying et al. [249] modified SA by grafting to enhance its removal efficiency. Therefore, sodium alginate-g- (polyacrylic acid-co-polymethacryloxyethyltrimethyl ammonium chloride) (SA-g-(PAA-co-PDMC)) was used to develop a pH-responsive polyampholyte superabsorbent polymer (SAP) able to adsorb anionic dyes. The behavior of Food Yellow 3 (FY3), the model dye in this study, was investigated, and pseudo-second-order kinetics were identified. The maximum adsorption capacity attained in this study was 654.87 mg/g, corresponding to a 98.23% removal efficiency.

However, Raza’s work described another exciting formulation for RB retention [234]. This formulation used a biomass polymer based on trans-anethole (ANE), maleic anhydride (MA), and SA as the main constituents for the hydrogel synthesis. Core–shell particles were first prepared using ANE and MA, and subsequently, its composite with SA was prepared under crosslinking with CaCl_2_ and glutaraldehyde. This hydrogel can also promote lead and cadmium removal and be regenerated and recycled. Langmuir and pseudo-second-order models were best fitted to describe the adsorption process, which indicated a somewhat similar removal performance for the three targeted pollutants, as follows: 745 mg/g for RB, 734.9 mg/g for Pb, and 722 mg/g for Cd.

Chitosan [219,241,243,248,251] is another polysaccharide that is a viable natural constituent for various synthetic counterparts, thanks to its adsorptive features rendered by amino and hydroxyl groups. Thus, hydrogels have been developed to promote the removal of multiple dyes. Li and co-workers [219] elaborated on a versatile hydrogel platform using a system consisting of CS (a natural polymer component), polyvinyl alcohol (PVA, a biocompatible polymer, used as the synthetic component), and carbon black (filler). This platform may be used for oil–water separation, dye removal, and water purification. The dyes of concern in this study were MG, FS, RB, CV, O II, SY, MB, and MO. Salzano de Luna et al. [236] elaborated on composite hydrogels using CS and hyper-crosslinked polymer (HCP) particles of the styrene–divinylbenzene copolymer. HCP particles were produced following a sequence of radical bulk polymerization and hyper-crosslinking involving a Friedel–Crafts reaction. Finally, CS/HCP composites were yielded by a phase inversion process. The use of HCP improved the mechanical properties of materials, which successfully removed dyes (IC; rhodamine 6G; SY) in repeated purification cycles. Liu et al. [238] performed the graft copolymerization of CS with poly(acrylamide-itaconic acid)(P(AM-co-IA)). This system was shown to remove CV efficiently. For a complete study, not only the polymer system of interest chitosan-graft-g-poly(acrylamide-itaconic acid)(CS-g-P(AM-IA)) was characterized, but also the reference counterparts (CS and CS-polyacrylamide (CS-PAM)). The CV removal efficiencies were 81.6% for CS-g-P(AM-IA), 51.7% for CS-g-PAM, and 36.5%, indicating that the addition of either AM or AM-IA moieties is beneficial for the adsorption process. Skoric and co. [246] elaborated on microparticles by approaching inverse suspension polymerization capable of removing textile dyes by photodegradative means. Microparticles were prepared using CS as a natural component and poly(methacrylic acid) as a synthetic one. Thus, the prepared microparticles were modified by immobilizing TiO_2_ on their surface. In this study, three types of dyes were considered: acid dyes (Acid Orange 7—AO 7, Acid Red 18—AR 18, Acid Blue 113—AB 113), reactive dyes (Reactive Yellow 17—RY 17, Reactive Black 5—RB 5), and direct dyes (Direct Blue 78—DB 78). Under illumination conditions, blank particles could only remove AB 113, while the microparticles with TiO_2_ removed all dyes in high yield; RY 17 being the only one removed to a lesser extent (75%).

Bacterial polysaccharides, such as salecan and pullulan, were also helpful in developing hydrogel structures for water purification. In this direction, Qi et al. [222] elaborated an innovative adsorbent system based on salecan. The authors reported that salecan underwent graft copolymerization with a synthetic copolymer, i.e., poly(acrylamide-co- itaconic acid) (P(AM-IA)). The adsorbent system was shown to degrade the MB dye, following the Freundlich model and pseudo-second-order kinetics. Saber-Samandari et al. [218] conducted the graft copolymerization of a natural polysaccharide, pullulan, with a synthetic component, polyacrylamide (PAAm). This hydrogel was tested on cationic (MB) and anionic dyes (reactive blue 2-RB). Adsorption experiments were carried out to yield adsorption capacities of 2 g RB/g and 2.5 g MB/g. Wu et al. [244] produced semi-IPN hydrogels using natural pullulan in conjunction with polydopamine (PDA). 1,6-hexanediol diglycidyl ether (HDE) carried out the crosslinking. The hydrogels were used to remove CV at a maximum adsorption of 107 mg/g; adsorption did not drop below 100 mg/g after four cycles. This study also showcased that PDA and HDE improved the mechanical properties of pullulan.

In a study by Bardajee et al. [231], a synthetic terpolymer, poly(3-sulfopropyl acrylate-co-acrylic acid-co-acrylamide), was grafted onto salep. Thus, hydrogels with adsorptive features toward (MO) were developed. The adsorption capacity of the synthesized hydrogel was 400 mg/g.

Starch is another natural polymer used for this purpose [217,220,224]. Zamani-Babgohari et al. [217] also obtained composites of AA and starch, but in a more complex formulation. Thus, in the first stage, the starch was modified with carboxymethyl moieties, forming carboxymethyl starch (CMS), which exhibited improved solubility, swelling, and adsorption properties. The as-synthesized CMS was co-polymerized with AA and acrylamide, using ammonium persulfate as an initiator and N,N’-methylene bis acrylamide as a crosslinking agent. Thus, a hybrid hydrogel with adsorbent features towards MB was prepared, with the maximum adsorption capacity in this study being 1700 mg/g. The idea of using CMS for MB removal was further supported by the work of Gong et al. [220], who elaborated on a magnetic composite hydrogel using a natural polymer, CMS sodium, and a biocompatible polymer, PVA. Moreover, magnetite was in situ prepared to endow the composite with magnetic features. MB was chosen for cleanup tests, and high removal efficiency was recorded over a wide range of pH values (4–9).

Rahman et al. [224] achieved the gamma radiation-initiated copolymerization of acrylic acid (AA) and potato starch to develop superabsorbent hydrogels for water treatment. Moreover, the effect of hydrogel treatment with KOH was also assayed. Therefore, two adsorbents were created, i.e., potato starch/AA/gamma radiation/KOH, and its reference (without KOH) counterpart. The system was suitable for removing heavy metals (Cr) and dyes (MB). KOH treatment was found to be beneficial for MB, but to affect Cr removal negatively.

#### 4.1.2. Hybrid Hydrogels Based on Gum Polymers for Dye’ Removal

In this subsection, three gum polymers are considered, i.e., gum ghatti (Gg) [212,233,237], Karaya gum (KG) [217,235], tragacanth gum (GumT) [240], and xanthan gum (XG) [245].

Gum polysaccharides, such as KG and Gg, may successfully be applied for the removal of three dyes (MG, CR, and RB). Moreover, their mechanical properties are improved when copolymerized with other synthetic polymers.

Other studies use natural polymers in the form of gum as precursors. In this context, it is worth mentioning the work of Mittal and co-workers [212], who developed a hybrid hydrogel system crosslinking an anionic polysaccharide, i.e., gum ghatti (Gg), with a co-polymer mixture of acrylamide and methacrylic acid, P(AAm-co-MAA), by approaching microwave assisted graft copolymerization. The system above promoted an advanced removal of two dyes, i.e., MB and MV, and may be involved in repeated purification cycles. Excellent adsorption efficiency occurred towards both dyes, following the Langmuir model.

CR is another dye that hybrids based on gum polymers may remove. Graft copolymers of Gg and acrylamide [233,237] were shown to achieve an advanced removal of CR. Mittal et al. [227] copolymerized Gg and PAAm, with the removal of four dyes (BG, RB, CR, and MO) in clay solutions being assayed under different conditions (pH, temperature, and polymer mass). It was found that the best removal performance occurred at a neutral pH. For all four dyes of interest, the adsorption process followed the Langmuir isotherm, with the dye’s removal performance decreasing as follows: BG (523.62 mg/g) > RB (421.60 mg/g) > CR (179.09 mg/g) > MO (173.69 mg/g).

Goddetti et al. [237] also copolymerized Gg with acrylamide, but this time, the copolymer was coated with zero-valent iron (ZVI), and a material that proved efficient in CR removal was developed. The elaborated system can act as an adsorbent for CR, with the adsorption following a pseudo-second-order model. The Langmuir adsorption capacity depends on the pH, with 153.8, 200, and 250 mg/g values at 25, 35, and 45 °C, respectively.

The PAAm-crosslinked Gg system can also remove other dyes (BG, RB, and CR) by flocculation and adsorption [233]. In this latter study, the adsorption process was best described by Langmuir isotherms, with qm values of 523.62 mg/g, 421.60 mg/g, 179.09 mg/g, and 173.69 mg/g for BG, RB, CR, and MO, respectively. In addition to the previously described systems, terpolymer formulations based on salep were also efficient.

Karaya gum (KG) is another complex polysaccharide comprising D-galacturonic acid, D-galactose, L-rhamnose, and small portions of D-glucuronic acid. The graft copolymerization of this polysaccharide with poly(2-(dimethylamino)ethyl methacrylate) to develop gels capable of removing dyes (MB and IC) from water has been described in the literature [217]. In this latter study, advanced adsorption was recorded for IC (a maximum adsorption capacity of 101.42 mg/g) compared to MB (a maximum adsorption capacity of 89.28 mg/g).

MG is an example of a dye that may be removed using hydrogels prepared using gum polymers as precursors. Mittal et al. [235] elaborated hydrogel nanocomposites by modifying a KG natural polymer through polyacrylic acid grafting and silicon carbide incorporation. The adsorbent features of this hydrogel were pointed out using two model dyes, i.e., MG and RB. For both dyes, the adsorption isotherm was best fitted with the Langmuir model, with the recorded maximum adsorption capacity being 757.57 mg/g for MG and 497.51 mg/g for RB. Yet, a better removal efficiency for MG was achieved (91%) than for RB (86%).

Copolymerization and simple mixing can lead to multiple composite configurations between natural and synthetic polymers. For instance, Mohamed et al. [245] prepared a composite using XG along with poly (N-vinyl imidazole), which was able to remove CV excellently, with a maximum adsorption capacity (Qm) of 453 mg/g. The adsorbed CV was desorbed with HCl to regenerate the adsorbent.

**Figure 16:**
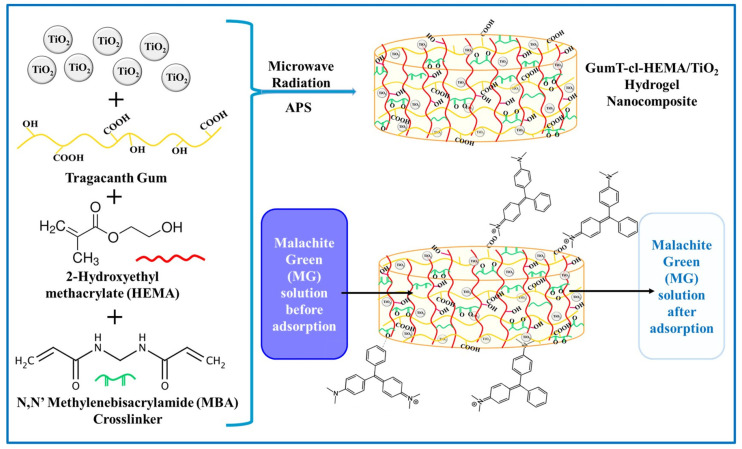
The synthesis process and schematic illustration of possible interactions between MG and GumT-cl-HEMA/TiO_2_ hydrogel composite (adapted from Ref. [240]).

Sharma et al. [240] developed a hydrogel nanocomposite using GumT as a polysaccharide precursor. Hydroxyethyl methacrylate (HEMA) was used as a crosslinking agent for GumT, whereas titania (TiO_2_) was used as a filler. This hydrogel nanocomposite shows adsorbent features towards MG. Figure 16 depicts the preparation process, showing the components of the system, their functional groups, and the preparation conditions. Moreover, the distribution of the functional groups of the components in the final structure of the hybrid is also highlighted. This distribution might be of certain help to understand the adsorption process, with MG probably being captured by MBA moieties. Moreover, it was proven that using TiO_2_ is beneficial for adsorption. Therefore, for the reference hydrogel, GumT-cl-HEMA, efficiencies of 91.3% and 84.2% were achieved after the 1st and 3rd adsorption cycles. In contrast, for the composite hydrogel, GumT-cl-HEMA/TiO_2_, higher adsorption efficiencies of 99.3% and 94.8% were recorded after the 1st and 3rd adsorption cycles, respectively.

Another dye removed using polysaccharides or other natural compounds is CV. Polysaccharides exhibit certain advantages related to their renewability and biocompatibility, which makes them suitable for water treatment. The polysaccharides used for this purpose are CS [243], pullulan [244], and xanthan gum (XG) [245].

#### 4.1.3. Hybrid Hydrogels Based on Other Kinds of Polymers for Dye’ Removal

Lignin, a critical component of plant tissue, and its derivative counterpart, lignosulfonate, were also shown to form hydrogels suitable for MB removal [216,224]. In addition, grafting poly(acrylic acid-r-acrylamide) also seems beneficial for adsorption. Yu et al. [216] further argue that it is helpful for the dye removal process to graft acid moieties to a lignosulfonate (LS)-based hydrogel, with an equilibrium of 2013 mg/g being attained.

Qian and co-workers [224] developed a hydrogel system combining three different constituents, i.e., lignin, poly(N-methyl aniline), and GO, which was used in its reduced form as a filler. The composite hydrogel registered a good adsorption capacity for heavy metals (Pb) and dyes (MB), with values of 753.5 mg/g and 201.7 mg/g, respectively.

Zhang et al. [221] raised the level of complexity by adding two natural polymers, i.e., carboxylated lactose, sodium lignosulfonate, a synthetic component, PAA, and a silver-based filler, to prepare the composite hydrogels for dye removal. Thus, a hybrid hydrogel with multiple anchoring points was developed, which allowed Ag^+^ to be transformed into silver nanoparticles. These nanoparticles degraded dyes (RB, MO, and MB) and p-nitrophenol.

Other studies elaborated formulations based on natural and synthetic polymers, eventually with fillers, and used them to develop hydrogels that may be involved in the adsorption of MG. For this purpose, Tang et al. [238] prepared LS-g-poly(acrylic acid-r-acrylamide) mesoporous materials with adsorption features towards MG. These properties may be attributed to the inherent functional groups of LS and the grafted counterparts.

Peighambardoust et al. [242] proposed an original approach to mitigate the problem of water pollution with cationic dyes. In this respect, a three-component composite hydrogel, containing polyacrylamide (PAAm) copolymerized with a natural polymer (gelatin) and a filler phase, was envisaged. The filler phase consisted of activated carbon derived from Luffa cylindrica biomass (ACL) used by itself or combined with Mg-Fe-layered double hydroxide (LDH). This study employed CV as a model dye, for which pseudo-second-order kinetics were identified. It was also found that the maximum monolayer adsorption capacity (qm) for PAM-g-gelatin, PAM-g-gelatin/ACL, and PAM-g-gelatin/ACL/Mg-Fe LDH were 35.45, 39.865, and 44.952 mg/g, respectively. Therefore, it may be assumed that the best adsorption capacity was attained when using Mg-Fe LDH as the filler.

### 4.2. Hybrid Hydrogels for Heavy Metals’ Retention

The second part of this section is dedicated to removing heavy metals using this kind of system: a natural polymer, synthetic polymer, and, eventually, a filler. For a more effortless follow-up, Table 7 summarizes some studies of interest regarding heavy metals’ removal by hybrid or composite hydrogels. The heavy metals removed using these kinds of hybrids are chromium—Cr [252,253,254,255,256]; copper—Cu [254,255,257,258,259,260,261,262]; nickel—Ni [257,261,263,264]; lead—Pb [254,257,259,263,265,266,267]; cadmium—Cd [257,261,263,264,265,267,268,269,270]; zinc—Zn [259,261,262,266]; tin—Sn [271]; and platinum—Pt [271]. The literature reveals that hydrogels, modified with magnetic particles, also show a good decontamination performance, and the improved and easier recycling of adsorbents [256]. Similarly to dyes, the information is listed considering the natural polymer and the same three subsections: polysaccharides, gum polymers, and other polymers.

#### 4.2.1. Hybrid Hydrogels Based on Polysaccharides for Heavy Metals’ Removal

Hybrid hydrogels prepared using cellulose and a derivative thereof may achieve the removal of five heavy metals: Cd, Pb, Ni [263], Pb, and Zn [266].

For instance, Zhou et al. [263] prepared hybrid hydrogels comprising natural and synthetic components by conducting the graft copolymerization of cellulose with acrylic acid. A study was carried out to determine the effect of the pH of the initial concentration of heavy metal ions and of the competitive ions (Cd, Pb, and Ni). The best results were recorded at pH = 5. Competitive adsorption tests revealed that Pb is the best adsorbed. The adsorption process followed pseudo-second-order kinetics, while the adsorption isotherms best fit the Langmuir model. Competitive adsorption tests revealed that affinity follows the order Pb > Ni > Cd.

Astrini et al. [266] elaborated a composite hydrogel with pH-dependent adsorption features consisting of a natural polymer (CMC), a synthetic polymer (PAA), and MMT as the filler. The copolymer of CMC and PAA was prepared by free-radical and solution polymerization, and MMT was added to the system. Heavy metal removal features were assessed towards Pb and Zn. The effect of competitive ion binding was also determined, on which occasion a higher binding efficiency was found towards Zn^2+^ vs. Pb^2+^. The best adsorption performance was recorded for pH = 5, at which Zn^2+^ adsorption was 286.67 mg/g, while for Pb^2+^, it was 146.19 mg/g. Moreover, at this same pH, the adsorbent was regenerated with mineral acid.

Alginate is another natural polysaccharide that may be used as a precursor for hybrid hydrogels that can accomplish an advanced removal of three heavy metals: Cu [254,272], Pb, and Cr [254].

Al- aidy El- saied et al. [272] elaborated on a superabsorbent material by grafting alginate on poly(2-acrylamido-2-methyl-1-propane sulfonic acid), followed by modification with nickel ferrite (NiFe_2_O_4_). The as-developed superabsorbent was suitable for removing heavy metals (copper being tested) and dyes (MB being tested). For Cu^2+^, the best adsorbent dose was 0.75 g/30 mL, and the best adsorption performance was recorded at pH = 4, whereas for MB, the best performances corresponded to an adsorbent dose of 0.65 g/30 mL and pH = 5.75. However, MB was the best removed (98.32% instead of 83.00% for Cu^2+^). The Freundlich model best represented Cu^2+^ isothermal adsorption, whereas, for MB, both Freundlich and Langmuir seem appropriate. The highest adsorption for MB was 275.6 mg/g, whereas for Cu^2+^, it was 22.81 mg/g.

An exciting formulation was proposed by Zhang et al. [254], who used synthetic PVA in a mixture with one (Alg) or two natural polymers (Alg and CS), and prepared two types of composites, i.e., PVA/Alg and PVA/Alg/CS. On both occasions, bacterial alginate (P. aeruginosa-derived Alg) was used. The authors state that this type of Alg performed better than its commercial counterpart in the performance of removing heavy metals (Pb, Cr, and Cu).

CS-based hydrogels show adsorptive features towards Cr [252,253], Cu [257,259,265,271], Zn [259,265,271], Pb [257,259,265,267,271], Cd [257,265,269], Sn [271], Co [257], Ni [257], and Hg [267].

Becalli Vilela et al. [252] elaborated on a hybrid hydrogel using CS and PAA as precursors, crosslinked with MBA. Four models were considered for investigating the adsorption of Cr (VI), namely Langmuir, Freundlich, Redlich–Peterson, and Sips, with Redlich–Peterson being found to describe the process better. Langmuir and Sips revealed a maximum adsorption capacity of Cr (VI) of 73.14 and 93.03 mg/g of the dried hydrogel. At pH = 4.5 and a 100 mg/L initial concentration, Cr (VI) was 94.72% removed.

Nowruzi and co-workers [253] elaborated on a three-component biocomposite comprising CS, PVA, and activated carbon, capable of removing Cr^2+^ from aqueous solutions. At pH = 4.9, a point-zero charge (PZC) is attained, but the best Cr^6+^ adsorption occurs at pH = 2. The experimental results fitted the pseudo-second-order kinetics and Langmuir isotherm well. According to the latter, the maximum amount of adsorbed Cr^2+^ was 1089 mg/g.

In this regard, Liu et al. [257] originally combined dextran and CS within a PAA matrix. This resin showed adsorbent features towards five heavy metal ions: Pb (395 mg/g), Cu (342 mg/g), Cd (269 mg/g), Co (232 mg/g), and Ni (184 mg/g). Moreover, the hybrid adsorbent retained at least 75% of the initial adsorption capacity after five adsorption–desorption cycles.

Zhao et al. [259] also elaborated on a system based on CS and PAAm. However, their study modified CS with α-ketoglutaric acid before using PAAm. Thus, a semi-IPN hydrogel was elaborated and used to remove heavy metal ions (Cu, Pb, and Zn). At 30 °C and pH = 5, the maximum adsorption capacities for Cu^2+^, Pb^2+^, and Zn^2+^ were 72.39, 61.41, and 51.89 mg/g, respectively. After five adsorption–desorption cycles, the adsorbent retained 90% of its initial adsorption capacity for all three metals.

Ma et al. [265] elaborated a double-networked hydrogel. In this study, one polymer chain was CS, whereas the other was PAAm. The crosslinking agents for CS and polyacrylamide were ethylenediaminetetra–acetic acid (EDTA) and N,N’-methylenebis(acrylamide)(MBA), respectively. The hydrogel proved viable and cost-effective for removing heavy metals (Cd, Cu, and Pb) with sorption capacities of 86.00, 99.44, and 138.41 mg/g, respectively.

In this respect, Zhu and co-workers [267] copolymerized CS with PAA. The graft copolymer was endowed with magnetic features by developing a Pickering emulsion system using Fe_3_O_4_ as a magnetic component. Magnetite was modified with an organosilane, i.e., 3-aminopropyltrimethoxysilane (APTES) or γ-methacryloxypropyltrimethoxysilane (γ-MPS). This system proved able to remove Cd and Pb. However, this system was more prone to adsorb Pb vs. Cd (695.22 mg/g adsorption capacity vs. 308.84 mg/g, respectively). Moreover, it is worth mentioning that the magnetic component favored the preservation of the adsorption capacity during five adsorption–desorption cycles.

**Figure 17:**
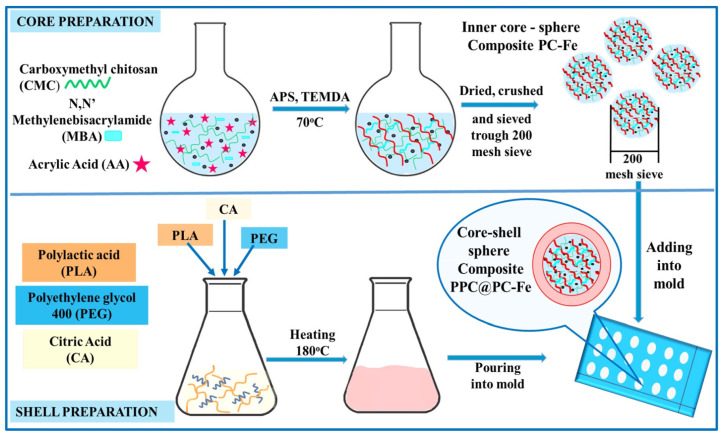
Schematic diagram of preparation procedure of PCC@PC-Fe core–shell hydrogel (adapted from Ref. [269]).

Hu et al. [269] elaborated on self-separating spheres of the core–shell type and used them to remove Cd from the soil (see Figure 17). The core consisted of magnetite (Fe_3_O_4_) embedded in a hydrogel prepared using carboxymethyl CS and acrylic acid. The shell consisted of an IPN of polyethylene glycol/polylactic acid (PEG/PLA), subsequently used to encapsulate citric acid. Figure 17 illustrates the preparation of the core–shell and targeted hybrid hydrogel, highlighting the steps involved in preparing the core and shell components. Thus, in the case of the core, the hydrogel structure was formed after heating at 70 °C. In the case of the shell, it was necessary to work at a higher temperature (180 °C) to bring the mixture into a liquid state, the melt, so it could be poured into the mold. These core–shell hybrid spheres promoted advanced cadmium removal through a two-stage process, as follows with Cd activation by the shell and Cd adsorption by the magnetite component of the core. Rather promising results were obtained as an amount of a 2% core–shell system significantly reduced the Cd content (from 6009 mg/kg to 4834 mg/g) of the soil in ten days.

Khademi et al. [271] copolymerized a natural polymer, CS, with acrylic acid and 2-acrylamido-2-metylpropanesulfonic acid (AMPS) using PEI and MBA as crosslinking agents. Therefore, a copolymer [CS-AMPS-AA/PEI-MBA] nanocomposite hydrogel was elaborated. This study employed three more raw materials, i.e., GO and silica as filler materials and ammonium persulfate as an initiator. The nanocomposite hydrogel was shown to promote the removal of Pb^2+^ and Sn^2+^ with maximum adsorption capacities of 263.16 and 188.188 mg/g, respectively. For both heavy metals, three successive adsorption–desorption cycles were assayed, with a slight decrease in absorbency being recorded from one cycle to another, as follows: 87.14, 80.63, and 71.71 for Pt; 86, 82, and 72 for Sn.

Starch can be used with synthetic polymers not only by copolymerization, but also by developing semi-IPNs of cryogels. Starch-based hybrid hydrogels were able to remove Cu [260,261], Cd [261], Ni [261], and Zn [261,270]. In another study, copolymers of starch and PAA were also synthesized and used for water treatment. However, polymerization was radically initiated and sodium humate (SH) was used for modification. This study targeted Cu removal, and a high adsorption capacity was recorded over a wide pH range (2.7–5.0). Moreover, repeated purification cycles were also carried out successfully. The use of SH does not lead to a significant change in the adsorption capacity, with the values varying in a narrow range (2.6–2.8). However, the only case where an increase in the qm value is recorded following the use of SH is at a content of 5%, suggesting that this percentage is the optimum [260]. In this context, the work of Apopei and co-workers [261], who elaborated on semi-IPNs of cryogels by copolymerizing anionically modified potato starch with PAAm, is worth mentioning. The cryogels were tested for removing four heavy metal ions. Langmuir, Freundlich, Temkin, and Sips models for isothermal adsorption were proposed, with the latter being the best-fitted. The maximum sorption capacity decreased as follows: Cu^2+^ (40.72 mg/g composite) > Cd^2+^ (19.27 mg/g composite) > Ni^2+^ (9.31 mg/g composite) > Zn^2+^ (7.48 mg/g composite). G. Zhou et al. [270] developed a double-network adsorbing system using amino-functionalized starch (NH_2_-starch) as a natural component and PAA as a synthetic component. This system proved to be a good option regarding adsorbing and regeneration features. Remarkable results were obtained regarding Cd removal, with an adsorption capacity of 256.4 mg/g. This sorbent may be used for column-based treatment. Other natural polymers that may be used to develop hydrogels for Cr removal are cotton-derived ones. Haque et al. [255] conducted the copolymerization of cotton with acrylic acid and acrylamide. Crosslinking was carried out using N, N’-methylene bis acrylamide (MBA), whereas potassium persulfate was the initiator.

#### 4.2.2. Hybrid Hydrogels Based on Gum Polymers for Heavy Metals’ Removal

Jafarigol et al. [264] elaborated on a dual-network hydrogel by approaching free-radical polymerization. This dual network consisted of poly(acrylamide-co-acrylic acid) and XG. Moreover, GO was also used for modification. Heavy metal removal tests for Ni and Cd were conducted, with good results regarding the removal yield and repeated use. Cd^2+^ was best adsorbed at pH = 7 for 0.01 g of adsorbent. The maximum adsorption capacity for Cd^2+^ was 312.15 mg/g, whereas for Ni^2+^, it was 185.00 mg/g. After four adsorption–desorption cycles for either of the investigated metals, the adsorbent kept about 80% of its initial activity. The adsorption and kinetics followed the Freundlich isotherm and pseudo-second-order kinetics, respectively.

#### 4.2.3. Hybrid Hydrogels Based on Other Kinds of Polymers for Heavy Metals’ Removal

Jamaluddin developed hydrogels using NR and acrylic acid, proving the system was suitable for Cu removal. For this aim, liquid NR was reacted with maleic anhydride and utilized in the malleated form. The highest removal yield of 72.19% was attained at 10 h contact time, a 47.66 mg/L initial Cu concentration, and 91 rpm stirring intensity [258].

Dolgormaa et al. [262] used a co-precipitation method to elaborate hybrids comprising a natural polymer (gelatin), a synthetic biocompatible polymer (PVA), and filler (super-paramagnetic iron particles-SPIONs). To properly assess the iron effect, reference adsorbents (prepared using only polymers, gelatin, and PVA) and hybrid counterparts of interest (gelatin, PVA, and SPIONs) were tested, and a comparison was made. The equilibrium adsorption capacities towards Cu^2+^ and Zn^2+^ for reference gels, were 18.067 mg/g and 14.143 mg/g, respectively. In contrast, for SPIONs-modified counterparts (gelatin, PVA, and SPIONs), the equilibrium adsorption capacities towards Cu^2+^ and Zn^2+^ were 12.904 mg/g and 9.303 mg/g, respectively. Even though the adsorption capacities decreased upon adding SPIONs, the stability of adsorbents was improved. Three adsorption–desorption cycles may be accomplished for both SPIONs-gelatin and SPIONs-gelatin-PVA, but the adsorption efficiency decreased with every step. After the third step, the Cu adsorption decreased by 5.05 and 7.28 for SPIONs-gelatin and SPIONs-gelatin-PVA, respectively, whereas for Zn, it decreased by 7.78 and 12.57%, respectively. Therefore, it may be assumed that the effect was more intense for zinc.

In an original attempt, Huang and co-workers immobilized CadRP on a PNIPAm gel network. This modification allowed the system to bind Cd reversibly, at the nanomolar level, from environmental water [268].

## 5. Conclusions and Outlook

Polymer-based hydrogels have emerged as versatile and efficient materials for the adsorption of organic compounds from industrial wastewater, including those from pharmaceutical, chemical, and food industries, as well as for agricultural activities and oil–water separation. For instance, hydrogels offer a promising solution for removing organic pollutants and chemical industrial residues from wastewater [273,274]. Although significant attention in recent years has also been directed toward removing antibiotics [275,276], fertilizers [277,278], and hydrophobic organic compounds [279,280], along with the effective separation of oil and water [281], this review has primarily dealt with the adsorption of organic and inorganic dyes and heavy metals, as highlighted in previous sections. According to the recent studies debated in this review, polymer-based hydrogels hold considerable potential as adsorbents for water and wastewater treatment, particularly in complex systems containing multiple components where the recovery of each element is required. However, water decontamination is a broad field, as many contaminants occur, requiring specific and efficient removal strategies. Consequently, substantial research gaps persist within hydrogels, particularly in addressing the diverse contaminants and tailoring hydrogel properties to meet these challenges effectively.

Another issue concerning the application of the newly developed adsorbents for industrial treatment processes is related to techno-economic factors. According to the adsorbents market, the segment has experienced continuous growth in various industries [282]. Yet, the main types of adsorbents have varied very little, being limited to activated carbon, zeolites, alumina, clays, manganese oxide, and cellulose. Thereby, although new generations of adsorbents have proven effective for retaining the proposed contaminants and the raw materials used for their design are cost-efficient, biofriendly, and/or biosourced, much work is still required for upscaling. Hence, apart from the limitations of hydrogel adsorbents in terms of the adsorption capacity, selectivity, stability, recovery, and reusability, the economic viability of such materials is also a hot topic for debate [283].

For starters, the cost of adsorbent materials is generally related to the synthesis method, the source of raw materials, and their properties, including recovery and regeneration. In addition to the adsorbent cost, the purification method and pollutant diversity and concentrations also influence the viability of introducing a “new type of adsorbent” on the market for wastewater treatment [283], not to mention the low flexibility of stakeholders for large investments in new technologies, especially when the old ones are still working. Therefore, from the perspective of industrial application in water treatment, hydrogel materials proposed for removing the various dyes and heavy metals lack viability for now. In fact, the hydrogel market is not even extended to the water treatment segment, since the main applications of hydrogels are found in the biomedical, agricultural, and environmental fields [284]. However, due to more stringent discharge limits set out for pollutants through the EU Urban Waste Water Treatment Directive [285], hydrogel materials with advanced adsorption features for dyes and heavy metal ions, along with new or upgraded procedures for water purification, may become a solution to comply with these new limits in the long term.

In the near future, several research advancements and strategies must be undertaken to advance hydrogel-based water treatment technologies and to improve their industrial implementation. Research should focus on creating hydrogels with enhanced stability under a wide range of environmental conditions, such as a fluctuating pH, temperature, and ionic strength. This can be achieved through crosslinking methods, incorporating stable polymer networks, or using composite hydrogels that combine natural and synthetic polymers for better durability. Investigating efficient, cost-effective, and energy-efficient regeneration methods is essential. Approaches like photocatalysis, electrochemical regeneration, or non-toxic solvents could be explored to restore hydrogel functionality. Research should also focus on developing hydrogels that can adsorb multiple contaminants, reducing the need for frequent regeneration. Focusing on less expensive raw materials and more scalable production processes, such as using waste materials (e.g., agricultural by-products) or biopolymers, could reduce costs. Additionally, advancing techniques for mass production, like 3D printing or solution-casting methods, may allow for more cost-effective hydrogel fabrication. Research should focus on designing hydrogels with selective adsorption capabilities for various contaminants, including heavy metals, or organic pollutants, and emerging contaminants like pharmaceuticals. This could be achieved by modifying the hydrogel surface with functional groups or using advanced material composites, as well as by developing ion-imprinted hydrogels or molecularly imprinted hydrogels.

Also, several strategies may be considered for industrial implementation. Before full-scale deployment, testing hydrogel-based systems in pilot-scale projects is crucial. This would help assess their performance, efficiency, and scalability in real-world water treatment scenarios, such as municipal water supplies, industrial effluents, or wastewater treatment plants. Hydrogel-based systems should be designed to integrate seamlessly into existing water treatment plants for industrial use. This includes designing modular, easy-to-install systems that can be retrofitted to current setups without significant infrastructure changes, thereby reducing implementation costs. By focusing on these research advancements and industrial strategies, hydrogel-based water treatment can be optimized and implemented globally, addressing water quality challenges efficiently and sustainably.

By advancing research in bio-based hydrogels, nanocomposite materials, and the integration of AI for design optimization, hydrogel-based water treatment technologies can become more sustainable, effective, and scalable. Renewable materials, enhanced adsorption and selectivity through nanocomposites, and the application of machine learning could drive the development of next-generation hydrogels, making them more viable for real-world, large-scale water treatment applications. Addressing scalability and industrial feasibility will ensure that these technologies can be adopted globally, solving water contamination issues efficiently and sustainably.

## Figures and Tables

**Table 1:** Preparation methods, performance, and reusability of various developed synthetic polymer-based hydrogel materials for organic dye retention.

Dyes	Hydrogel-Based Materials	Preparation Method	Performance	**Regeneration and Reusability**	**Ref.**
MB cationic dye RBBR anionic dye	- Monomers: PCL and HEMA - Initiator: AIBN	- by radical copolymerization	- maximum swelling equilibrium (%)—929% - removal efficiency (%)—95% cationic dyes (MB) and 40% anionic dyes (RBBR) - adsorption capacity (Q_max_, mg/g)—521.39 mg/g for MB (24 h, c_i_ = 100 mg/L, pH = 12) and 197.84 mg/g for RBBR (24 h, c_0_ = 100 mg/L, pH = 2) - best-fit kinetic—pseudo-second-order kinetic model (R^2^ = 0.992) - best fit isotherm—Langmuir isotherm (R^2^ = 0.999) - negative ΔG^0^ values; positive ΔH^0^ and ΔS^0^ values indicate that MB and RBBR adsorption spontaneous and thermodynamically favorable	- desorption eluent: HCl and NaOH solutions - 10th regeneration run - 10th reusable run with 80% removal efficiency	[44]
MV cationic dye	- Monomers: AM, IA - Polymer: PEG1500 - Crosslinker: NMBA - Initiator: KPS	- by free-radical crosslinking copolymerization	- maximum swelling equilibrium (%)—4500% - removal efficiency (%):— - adsorption capacity (Q_max_, mg/g)—28 mg/L*gm - best fit kinetic:— - best fit isotherm:— - thermodynamic:—	-	[81]
EB, ARS anionic dyes CV cationic dye	- Monomers: AA and HEMA - Crosslinker: ILA: 1VIM and 4VBC; - ILB: 1VIM and 4VBC and DMAEMA our NMBA - Initiator: APS andTEMED	- by redox polymerization technique using a redox initiator	- maximum swelling equilibrium (%)—929% - removal efficiency (%)—99.8% for anionic dyes (EB) and 72.4% for cationic dyes (CV) - adsorption capacity (Qmax, mg/g)—119 mg/g (48 h, c_0_ = 250 mg/L, pH = 7) - best-fit kinetic—pseudo-second-order kinetic model (R^2^ ≥ 0.997) - best-fit isotherm—Langmuir isotherm (R^2^ = 0.999) - thermodynamic equilibrium constants:—	- desorption eluent: HCl and NaOH solutions - 3rd regeneration run - 3rd reusable run with >80% removal efficiency	[82]
MB, AO and CG cationic dyes	- Monomers: AA, NIPAAM, N-allylisatin - Crosslinker: EGDMA - Initiator: AIBN	- by inverse microemulsion polymerization, free-radical polymerization	- maximum swelling equilibrium (%)—36,790% - removal efficiency (%)—98.24% (MB), 87.42% (AO), and 70.12% (CG) - adsorption capacity (Q_max_, mg/g)—584.78 mg/g (MB), 347.17 mg/g (AO), and 212.69 mg/g (CG) (48 h, c_0_ = 250 mg/L, natural pH value of dye solutions) - best-fit kinetic:— - best-fit isotherm—Langmuir and Freundlich isotherm - (R^2^ > 1), the kinetic and equilibrium adsorption isotherms failed to explain the adsorption process due to the extremely small size - thermodynamic:—	- desorption eluent HCl our NaOH, washed with bidistilled water -2nd regeneration cycles with percent desorption efficiency 100 (MB), 92 (AO), and 86 (CG) (%); -2nd reusable cycles	[83]
CCA anionic dye	- Monomers: AM, APTMACl - Crosslinker: PEGDMA - Initiator system: APS, TEMED	- by free-radical crosslinking copolymerization	- maximum swelling equilibrium (%)—1198% - removal efficiency (%)—99% - adsorption capacity (Q_max_, mg/g)—79.42 mg/g (48 h, c_0_ = 200 mg/L, pH = 7) - best-fit kinetic:— - best-fit isotherm—Langmuir (R^2^ = 0.9952)- thermodynamic: —	-	[84]
MB cationic dye MO anionic dye	- Monomers: AA - Crosslinker: NMBA - Initiator: APS	- by free-radical polymerization	- maximum swelling equilibrium:— - removal efficiency (%)—99.99% for MB and 17.5% for MO - adsorption capacity (Q_max_, mg/g)—454.45 mg/g for MB (58 h, c_0_ = 1500 mg/L, pH = 8) - best-fit kinetic—pseudo-first-order model (R^2^ = 0.9946) for MB - best-fit isotherm—Redlich–Peterson and Sips isotherm models (R^2^ = 0.998) for MB - thermodynamic equilibrium constants—ΔG^0^ = −3.7 kJ/mol; ΔH^0^ = 65.6 kJ/mol, ΔS^0^ = 221.4 J/K*mol for MB, thermodynamically spontaneous, favorable, and endothermic	- desorption eluent: inorganic acid solutions - 5th regeneration run - 5th reusable run with >87% removal efficiency	[85]
MB cationic dye	- Monomers: AA and AM - Crosslinker: NMBA - Initiator:APS	- by free-radical polymerization	- removal efficiency (%)—90% - adsorption capacity (Q_max_, mg/g)—1315 mg/g (12 h, c_0_ = 50 mg/L, pH = 8) - best-fit kinetic—pseudo-second-order model (R^2^ = 0.99) - best-fit isotherm—Dubinin–Radushkevich model (R^2^ = 0.99) - thermodynamic equilibrium constants: ΔG^0^ = −40 kJ/mol; ΔH^0^ = −51 kJ/mol, ΔS^0^ = −51 kJ/K*mol; adsorption with an endothermic chemical integration	- desorption eluent: NaNO_3_, washed with bidistilled water and dried - 20th regeneration run - 21st reusable run	[86]
MB cationic dye	- Monomers: IA, EG, AA - Crosslinker: NMBA - Initiator: APS	- by free-radical polymerization	- maximum swelling equilibrium (%)—600% - removal efficiency (%)—83% - adsorption capacity (Q_max_, mg/g)—1270 mg/g - best-fit kinetic:— - best-fit isotherm:— - thermodynamic equilibrium constants:—	-	[87]
RhB cationic dye	- Monomers: IA, AA, and ANi - Crosslinker: NMBA - Initiator: KPS	- by free-radical crosslinking copolymerization	- maximum swelling equilibrium (%)—1755.3% - removal efficiency (%)—87.9% - adsorption capacity (Q_max_, mg/g)—925.92 mg/g for MB (60 min, c_0_ = 50 mg/L, pH = 7) - best-fit kinetic—pseudo-first-order model (R^2^ = 0.92) for MB - best-fit isotherm—Freundlich isotherms (R^2^ = 0.996) - thermodynamic equilibrium constants:—	- desorption eluent: Cl and NaOH solutions - 4th regeneration run - 4th reusable run with >85% removal efficiency	[88]
MB cationic dye MO anionic dye	- Monomers: AA and ACN - Homopolymer: PDMAEMA - Crosslinker: NMBA - Initiator: AIBN	- by combining distillation precipitation polymerization and RAFT polymerization	- maximum swelling equilibrium:— - removal efficiency (%)—76.6% for MO and 48.3% for MB - adsorption capacity (Q_max_, mg/g)—54.4 mg/g for MO (c_0_ = 12 mg/L, pH = 2) and 35.9 mg/g for MB (c_i_ = 12 mg/L, pH = 10) - best-fit kinetic—pseudo-second-order kinetic model (R^2^ =0.99) - best-fit isotherm—Freundlich model isotherm (R^2^ = 0.999)	-	[89]
MB cationic dye	- Monomers: AM, AA, and AHPS - Crosslinker: PTE - Initiator: BzO_2_	- by free-radical polymerization	- maximum swelling equilibrium (%)—7071%- removal efficiency (%):— - adsorption capacity (Q_max_, mg/g)—79.42 mg/g (8 h, c_0_ = 100 mg/L, pH = 7) - best-fit kinetic— pseudo-second-order kinetic model (R^2^ = 0.999). - best-fit isotherm— Freundlich isotherm (R^2^ =0.957) - thermodynamic:—	-	[90]
CV cationic dye CR anionic dye	- Monomers: APTMACl and AMPSA - Crosslinker: PEGDMA - Initiator system: APS and TEMED	- by free-radical crosslinking copolymerization	- maximum swelling equilibrium (%)—3715% - removal efficiency (%)—98% for CV and 86% for CR - adsorption capacity (Q_max_, mg/g)—13.6 mg/g for CV (14 h, c_0_ = 50 mg/L, pH = 9) and 9.07 mg/g for CR (14 h, c_0_ = 50 mg/L, pH = 5) - best-fit kinetic—pseudo-second-order kinetics model (R^2^ = 0.9930) - best-fit isotherm—Freundlich isotherms (R^2^ = 0.98) - thermodynamic equilibrium constants— ΔG^0^ = −5.869 and −0.54 kJ/mol; ΔH^0^ = 63.93 and 66.09 kJ/mol, ΔS^0^ = 0.23 and 0.22 J/K*mol for CV and CR, respectively	- with acetone via the solvent extraction method - 5th regeneration run - 5th reusable run with >75% removal efficiency	[91]
MB cationic dye RBBR anionic dye	- Monomers: PCL and HEMA - Initiator: AIBN	- by radical copolymerization	- maximum swelling equilibrium (%)—929% - removal efficiency (%)—95% cationic dyes (MB) and 40% anionic dyes (RBBR) - adsorption capacity (Q_max_, mg/g)—521.39 mg/g for MB (100 min, c_0_ = 50 mg/L, pH = 6) and 197.84 mg/g for RBBR (100 min, c_0_ = 50 mg/L, pH = 6) - best-fit kinetic— pseudo-second-order kinetic model (R^2^ = 0.992) - best-fit isotherm—Langmuir isotherm (R^2^ = 0.999) - negative ΔG^0^ values; positive ΔH^0^ and ΔS^0^ values indicate that MB and RBBR adsorption is spontaneous and thermodynamically favorable	- desorption eluent: HCl and NaOH solutions - 10th regeneration run - 10th reusable run with 80% removal efficiency	[92]
MO CR CCB anionic dye	- Monomers: PQ-10 - Crosslinker: PEGDMA	- by electron beam irradiation crosslinking technique	- maximum swelling equilibrium (%)—704%- removal efficiency (%):— - adsorption capacity (Q_max_, mg/g)—628.93% for MO; 427.35% for CR; and 628.93% for CBR (24 h, c_0_ = 25 mg/L, pH = 8) - best-fit kinetic—kinetic pseudo-second-order model (R^2^ = 0.992) - best-fit isotherm—Langmuir isotherm (R^2^ = 0.999)	- desorption eluent: HCl solutions - 5th regeneration run - 5th reusable run with 86% removal efficiency	[93]
MB cationic dye MO, CR, EBT anionic dye	- Monomers: AA and DADMAC - Crosslinker: CNS - Initiator: APS, TEMED	- by free-radical crosslinking copolymerization	- maximum swelling equilibrium (%)—704% - removal efficiency (%):— - adsorption capacity (Q_max_, mg/g)—409% for MO, 862% for CR, 1139% for EBT, and 722 mg/g for MB (135 min, c_0_ = 500 mg/L, pH = 7) - best-fit kinetic—kinetic pseudo-first-order model (R^2^ = 0.965) - best-fit isotherm—Sips isotherm (R^2^ = 0.992)	-	[94]
MO, AR anionic dye	- Monomers: AETAC, NVP - Crosslinker: NMBA - Initiator: APS, TEMED	- by free-radical crosslinking copolymerization	- maximum swelling equilibrium (%)—28.12 g/g - removal efficiency (%)—74% for CA, 77% for CV, and 62% for MV - adsorption capacity (Q_max_, mg/g)—905.60 mg/g for MO; 843.58 mg/g for AR (24 h, c_0_ = 2000 mg/L, pH ≤ 5) - best-fit kinetic—kinetic intraparticle diffusion model (R^2^ = 0.99) - best-fit isotherm—Freundlich isotherm (R^2^ = 0.99) -negative ΔG^0^ values; positive ΔH^0^ and ΔS^0^ values indicate the adsorption is spontaneous and thermodynamically favorable and is physical		[95]
MB cationic dye	- Monomers: AA, MAC - Crosslinker: CNS - Initiator: APS, TEMED	- by free-radical crosslinking copolymerization	- maximum swelling equilibrium—3800 g/g - removal efficiency (%)—98% - adsorption capacity (Q_max_, mg/g)—2249 mg/g (1 h, c_0_ = 400 mg/L, pH = 7) - best-fit kinetic—kinetic pseudo-second-order model (R^2^ = 0.99) - best-fit isotherm—Langmuir isotherm (R^2^ = 0.998)	-	[96]
CV, MV, CA anionic dye	- Monomers: AM, AA, AETAC - Crosslinker: EDMA - Initiator: APS, TEMED	- by free-radical crosslinking copolymerization	- maximum swelling equilibrium—28.12 g/g - removal efficiency (%)—97% for MO and 98.6% for AR - adsorption capacity (Q_max_, mg/g)—2.445 mg/g for CA; 0.156 mg/g for CV; and 0.115 mg/g for MV (24 h, c_0_ = 10 mg/L, natural pH of the dye solution) - best-fit kinetic:— - best-fit isotherm— Langmuir model for CA, Saraydın model isotherm for CV and MV	-	[97]
AR1, AY36, DR23, AO7, and DB15 anionic dyes	- Monomers: EDA, EPC - Crosslinker: NMBA - Initiator: APS	- by one-step cross-linked copolymerization	- maximum swelling equilibrium:— - removal efficiency (%)—99.1%, 99.8%, 98.0%, 98.8%, and 100% for DR23, AR1, AO7, AY36, and DB15, respectively - adsorption capacity (Q_max_, mg/g)—1541.64 mg/g (DR23) (24 h, c_0_ = 10 mg/L, pH = 2–12) - best-fit kinetic: pseudo-second-order model—(R^2^ = 0.9991) - best-fit isotherm: Langmuir isotherm—(R^2^ =0.9995) - thermodynamic:—	- desorption experiments were washed twice with distilled water	[98]
MO anionic dye	- Monomers: DMC, AM - Crosslinker: NMBA - Initiator: APS	- by precipitated droplets’ in situ crosslinking polymerization	- maximum swelling equilibrium:— - removal efficiency (%)—94% - adsorption capacity (Q_max_, mg/g)—992.63 mg/g for OG, 1388.55 mg/g for AR, and 744.17 mg/g for MO (24 h, c_0_ = 10 µmol/L, pH = 3–9) - best-fit kinetic: pseudo-second-order model—(R^2^ = 0.99) - best-fit isotherm: Langmuir isotherm—(R^2^ =0.99) - thermodynamic:—	- desorption eluent: HCl solutions - 5th regeneration run - 5th reusable run with 80% removal efficiency	[99]

‘—’ indicates that no value is available for this field.

**Table 2:** Preparation methods, performance, and reusability of different developed synthetic polymer-based composite hydrogel materials for organic dyes’ retention.

Dyes	Composite Hydrogel-Based Materials	Preparation Method	Performance	Regeneration and Reusability	Ref.
MB, cationic dye	- Homopolymer: PVA - Filler/Crosslinker: Bentonite Initiator:—	- by an innocuous physical crosslinking method	- maximum swelling equilibrium:— - removal efficiency (%)—93% - adsorption capacity (Q_max,_ mg/g)—27 mg/g (15 h, c_0_ = 670 mg/L) - best-fit kinetic— pseudo-second-order model -(R^2^ = 0.999) - best-fit isotherm— Freundlich isotherm model (R^2^ = 0.993)	- desorption eluent: HCl solutions - 5th regeneration run - 5th reusable run with 80% removal efficiency for MB	[45]
MB, cationic dye MO anionic dye	- Monomer: AM - Filler: ZnO - Crosslinker: NMBA - Initiator: ZnO photocatalytic initiator	- self-initiated photopolymerization	- maximum swelling equilibrium—1.46 g/g - adsorption capacity (Q_max,_ mg/g)—0.9 mg/g for MB (11 h, c_0_ = 5 mg/L, pH = 6), 0.025 mg/g for MO (11 h, c_i_ = 5 mg/L, pH = 2) - best-fit kinetic—pseudo-second-order model (R^2^ = 0.99) - best-fit isotherm—Langmuir isotherm model (R^2^ > 0.998)	- desorption eluent: NaOH solutions - 15th regeneration run - 15th reusable run with 81% and 75% removal efficiency for MB and MO, respectively	[46]
MB, cationic dye	- Homopolymer: PVA, PHPAm - Filler: GO - Crosslinker: Chromium III acetate	- a one-stage approach via the internal ionic gelation	- removal efficiency (%)—99% - adsorption capacity (Q_max,_ mg/g)—714.8 mg/g for MB (3 h, c_0_ = 200 mg/L, pH = 7) - best-fit kinetic— pseudo-second-order model (R^2^ = 0.997) - best-fit isotherm—Langmuir isotherm model (R^2^ = 0.994) - negative ΔG^0^ values; positive ΔH^0^ and ΔS^0^ values; indicate the adsorption was a spontaneous, endothermic, and physical adsorption process.	- desorption eluent: HCl solutions - 5th regeneration run - 5th reusable run with 70% removal efficiency	[47]
MG anionic dye CR cationic dye	- Monomers: AA, - Homopolymer: PVA - Filler: GO - Crosslinker: NMBA - Initiator: APS	- by free-radical crosslinking copolymerization	- maximum swelling equilibrium:— - removal efficiency (%)—93% for MG and 55% for CR - adsorption capacity (Q_max,_ mg/g)—1458 mg/g for MG and 826 mg/g for CR (30 min, c_0_ = 100 mg/L, pH = 3–5) - best-fit kinetic—pseudo-second-order model (R^2^ = 0.999) - best-fit isotherm—Freundlich isotherm (R^2^ = 0.984)	- desorption eluent: HCl and NaOH solutions - 5th regeneration run - 5th reusable run with 74% and 43% removal efficiency for MG and CR, respectively	[101]
MB, CV and SO cationic dye	- Monomer: AA - Filler: Melamine-coated BIO-Ox - Crosslinker: NMBA - Initiator: AIBN	- by in situ free-radical polymerization	- maximum swelling equilibrium(%)—60% - removal efficiency (%)—97% for CV, 99% for SO, 95% for MB - adsorption capacity (Q_max,_ mg/g)—463 mg/g for CV, 711 mg/g for SO, and 638 mg/g for MB (2 h, c_0_ = 200 mg/L, pH = 10 for MB, pH = 9 for CV, and pH = 7 for SO) - best-fit kinetic—pseudo-second-order model (R^2^ = 0.999) - best-fit isotherm—Langmuir isotherm model (R^2^ > 0.998)	- desorption eluent: NaOH solutions - 7th regeneration run - 7th reusable run with 71 removal efficiency for MG	[102]
MB, cationic dye MO anionic dye	- Homopolymer: PVA - Filler/Crosslinker: AC, FeCl_3_, FeSO_4_*7H_2_O	- a one-pot approach	- maximum swelling equilibrium:— - removal efficiency (%)—99% - adsorption capacity (Q_max,_mg/g)—174 mg/g for MB, 147 mg/g for MO (5 h, c_0_ = 5 m, pH = 11) - best-fit kinetic—pseudo-second-order model (R^2^ = 0.999) - best-fit isotherm—Langmuir isotherm model (R^2^ > 0.992)	- desorption eluent: ethanol - 5th regeneration run - 5th reusables run with 72% removal efficiency for MB.	[103]
MB, cationic dye	- Homopolymer: PVA - Fillers: TiO_2_ and Na_2_CO_3_ - Crosslinker: NMBA - Initiator: KPS	-by freeze–thaw cycle method	- maximum swelling equilibrium:— - removal efficiency (%)—99.85% - adsorption capacity (Q_max,_ mg/g)—318.47 mg/g 303.83 mg/g (12 h, c_0_ = 100 mg/L) - best-fit kinetic—pseudo-second-order model (R^2^ = 0.999) - best-fit isotherm—Langmuir isotherm model (R^2^ = 0.999)	-	[104]
MB, cationic dye	- Monomer: MAAn - Fillers: TiO_2_, ZnO - Crosslinker: EGDMA - Initiator: Darocur1173	- by water-in-oil-in-water double emulsion	- maximum swelling equilibrium:— - removal efficiency (%)—100% (24 h, c_0_ = 5 mg/L, pH = 3–5) - adsorption capacity (Q_max,_ mg/g):— - best-fit kinetic:— - best-fit isotherm:—	-	[105]
MB, cationic dye CR anionic dye	- Monomer: AM, AA, IA - Filler: TiO_2_ - Crosslinker: NMBA - Initiator: TiO_2_	- by photopolymerization	- maximum swelling equilibrium(%)—473 g/g - removal efficiency (%)—90% for MB; 91% for CR - adsorption capacity (Q_max,_ mg/g)—4.86 for MB, 2.22 mg/g for CR (24 h, c_0_ = 50 mg/L) - best-fit kinetic—pseudo-second-order model (R^2^ = 0.999) - best-fit isotherm—Freundlich isotherm model (R^2^ = 0.999)	-	[106]
MB, cationic dye	- Monomer: AA - Filler: Lap - Crosslinker: NMBA - Initiator: KPS	- by in situ free-radical polymerization	- maximum swelling equilibrium(%)—1360 g/g - removal efficiency (%)—98% - adsorption capacity (Q_max,_ mg/g)—3846 mg/g (12 h, c_0_ = 1000 mg/L, pH = 7) - best-fit kinetic—pseudo-second-order model (R^2^ = 0.999) - best-fit isotherm—Langmuir isotherm model (R^2^ = 0.999) - negative ΔG^0^ values; positive ΔH^0^ and ΔS^0^ values; indicate the adsorption was a spontaneous, endothermic, and physical adsorption process	- desorption eluent: HCl and NaOH solutions - 6th regeneration run - 6th reusable run with 93% removal efficiency for MB	[107]
MB, cationic dye	- Monomer: MAA, AM - Filler: Cloisite 30B - Crosslinker: NMBA - Initiator: KPS	- by in situ free-radical polymerization	- maximum swelling equilibrium(%)—2800% - removal efficiency (%)—98.6% - adsorption capacity (Q_max,_ mg/g)—32.83 mg/gMB (1 h, c_0_ = 1500 mg/L, pH = 8) - best-fit kinetic—pseudo-first-order model (R^2^ = 0.98) - best-fit isotherm—Freundlich isotherm model (R^2^ = 0.97) - negative ΔG^0^, ΔH^0^,and ΔS^0^ values; indicate the adsorption was a spontaneous and exothermic process	-	[108]
MB, cationic dye	- Monomer: AA, MAC - Filler/Crosslinker: CNS - Initiator: APS, TEMED	- by in situ free-radical copolymerization	- maximum swelling equilibrium(%)—3800 g/g - removal efficiency (%)—98% - adsorption capacity (Q_max,_ mg/g)—2249 mg/g (1 h, c_0_ = 1500 mg/L, pH = 8) - best-fit kinetic—pseudo-second-order model (R^2^ = 0.99) - best-fit isotherm—Langmuir isotherm model (R^2^ = 0.99)	-	[109]
MB, cationic dye	- Monomer: NIPAM, IA - Filler: Pumice (mineral) -Crosslinker:— - Initiator:—	- by γ-radiation copolymerization	- maximum swelling equilibrium(%)—5000% - removal efficiency (%)—30% - adsorption capacity (Q_max,_ mg/g)—22.52 mg/24 h, c_0_ = 150 mg/L, pH = 7 - best-fit kinetic: - - best-fit isotherm—Langmuir isotherm model (R^2^ = 0.993)	-	[110]
BY28 cationic dye	- Monomer: MAA - Filler: ZSM-5 - Crosslinker: NMBA - Initiator: AIPH	- by free-radical polymerization	- maximum swelling equilibrium:— - removal efficiency (%):— - adsorption capacity (Q_max,_ mg/g)—180 mg/g48 h, c_0_ = 50 mg/L, pH = 6.8 - best-fit kinetic—pseudo-first-order model (R^2^ = 0.99) - best-fit isotherm—Freundlich isotherm model (R^2^ = 0.98)	-	[111]
MO anionic dye	- Monomers: MEMA - Filler: GO - Crosslinker: NMBA - Initiator: APS	- by in situ free-radical polymerization	- maximum swelling equilibrium:— - removal efficiency (%)—94% - adsorption capacity (Q_max,_ mg/g)—243.63 mg/g (8.5 h, c_0_ = 2500 mg/L, pH = 2) - best-fit kinetic—pseudo-second-order model—(R^2^ = 0.984) - best-fit isotherm—Langmuir isotherm—(R^2^ = 0.99) - negative ΔG^0^ values; positive ΔH^0^ and ΔS^0^ values indicate the adsorption is spontaneous and endothermic, driven by entropy	- desorption eluent: HCl and NaOH solutions - 8th regeneration run - 8th reusable run with 95% removal efficiency	[112]
MG, RhB cationic dye	- Monomer: AA - Filler: Curcumin-modified BIO - Crosslinker: NMBA - Initiator: AIBN	- by in situ free-radical polymerization	- maximum swelling equilibrium(%)—60% - removal efficiency (%)—98.75% for MG and 98.50% for RhB - adsorption capacity (Q_max,_ mg/g)—521 mg/g for MG (8.5 h, c_0_ = 1200 mg/L, pH = 7) and 742 mg/g for RhB (8.5 h, c_i_ = 1200 mg/L, pH = 6) - best-fit kinetic—pseudo-second-order model (R^2^ = 0.998) and Elovich (R^2^ = 0.995) - best-fit isotherm—Koble–Corrigan and Langmuir isotherm models (R^2^ > 0.997)	- desorption eluent: HCl and NaOH solutions - 7th regeneration run - 7th reusables run with 80% and 72% removal efficiency for MG and RhB, respectively	[113]
MB, cationic dye	- Monomer: AA, PVP - Filler: RPGS - Crosslinker: NMBA - Initiator: KPS	- by radical polymerization	- maximum swelling equilibrium:— - removal efficiency (%):— - adsorption capacity (Q_max,_ mg/g)—1815 mg/g (2 h, c_0_ = 200 mg/L, pH = 7) - best-fit kinetic—pseudo-second-order model (R^2^ = 0.999) - best-fit isotherm—Langmuir isotherm model (R^2^ = 0.995) - negative ΔG^0^_,_ ΔH^0^, and ΔS^0^ values; indicate the adsorption was a spontaneous and exothermic process	-	[114]
MB, CV cationic dye CR anionic dye	- Monomer: AM - Homopolymer: PVA - Filler: SiO_2_-coated ZnO or TiO_2_ particles - Crosslinker: NMBA - Initiator: CAN	- by free-radical crosslinking copolymerization	- maximum swelling equilibrium(%)—6400% - removal efficiency (%)—90% (MB, CV, and CR) - adsorption capacity (Q_max,_ mg/g)—703 mg/g for MB, 863 mg/g for CV, and 174 mg/g for CR (24 h, c_0_ = 4000 mg/L, pH = 4–10) - best-fit kinetic—pseudo-second-order model (R^2^ = 0.999) - best-fit isotherm—Freundlich isotherm model (R^2^ = 0.992)	- desorption eluent: HCl solutions - 5th regeneration run - 5th reusable run with 23% for MB, 30% for CV, and 13% for CR; removal efficiency	[115]

‘—’ indicates that no value is available for this field.

**Table 3:** Preparation methods, performance, and reusability of various developed synthetic polymer-based hydrogel materials for heavy metals’ retention.

Heavy Metals	Hydrogel/Composite Hydrogel-Based Materials	Preparation Method	Performance	Regeneration and Reusability	Ref.
Cu^2+^	- Monomer: AM, IA - Crosslinker: NMBA - Initiator: KPS, TEMEDA	- by radical polymerization	- maximum swelling equilibrium(%)—64% - removal efficiency (%)—98% - adsorption capacity (Q_max,_ mg/g)—336 mg Cu/g (tc = 48 h, c_0_ = 1750 mg/L)	- desorption eluent: NaOH solution - 6th regeneration run - 6th reusable run with 83% removal efficiency	[116]
Hg^2+^	- Monomer: NVP, IA - Homopolymer: PVA - Crosslinker: NMBA - Initiator: Pn-In	- by photopolymerization	- maximum swelling equilibrium: 57%- removal efficiency (%):— - adsorption capacity (Q_max,_ mg/g)—56 mg Hg/g (tc = 48 h, c_0_ = 1000 mg/L)	-	[117]
Pb^2+^	- Monomer: AA, AN, amidoxime group - Crosslinker: NMBA - Initiator: HHMP	- by cryo-polymerization	- maximum swelling equilibrium(%)—40% - removal efficiency (%)—99.8% - adsorption capacity (Q_max,_ mg/g)—450 mg Pb/g and 650 mg Pb/g after regeneration (tc = 24 h, c_0_ = 100 mg/L) - best-fit kinetic—pseudo-second-order kinetic (R^2^ = 0.999)	- desorption eluent: alkali solution - 7th regeneration run - 7th reusable run with 90% removal efficiency	[118]
Cu^2+^	- Monomer: AA, NIPAM, - Crosslinker: NMBA - Initiator: AIBN	- by RAFT polymerization	- removal efficiency (%)—69.8% - adsorption capacity (Q_max,_ mg/g)—435 mg Cu/g (c_0_ = 84 ppm)	-	[119]
Cu^2+^, Cd^2+^, Cr^6+^, Fe^3+^, Mn^2+^, Ni^2+^, Zn^2+^, Ce^3+^ and Ag^+^	- Monomer: AA, - Crosslinker: CNS - Initiator: APS, TEMEDA	- by free-radical polymerization	- maximum swelling equilibrium(%)—8130% - removal efficiency (%)—values for Cu^2+^, Cd^2+^, Mn^2+^, Ni^2+^, Zn^2+^, and Ce^3+^ exceeding 50% - adsorption capacity (Q_max,_ mg/g)—132.9 mg Cu/g (c_0_ = 0.16 mg/L), 132.9 mg Cd/g (c_0_ = 0.56 mg/L), 58.1 mg Cr/g (c_0_ = 0.52 mg/L), 12.4 mg Fe/g (c_0_ = 0.56 mg/L), 120.4 mg Mn/g (c_0_ = 0.55 mg/L), 128.8 mg Ni/g (c_0_ = 0.59 mg/L), 157.8 mg Zn/g (c_0_ = 0.65 mg/L), 203.5 mg Ce/g (c_0_ = 0.7 mg/L), and 203.5 mg Ag/g (c_0_ = 1.08 mg/L, tc = 4 day) - best-fit kinetic—pseudo-second-order kinetic (R^2^ = 0.98) - best-fit isotherm—Freundlich isotherm (R^2^ = 0.95)	- desorption eluent: alkali solution - 10th regeneration run - 10th reusable run with 70% removal efficiency	[120]
Co^2+^	- Monomer: AA, AM - Homopolymer: PVA - Crosslinker: NMBA - Initiator: KPS	- by free-radical polymerization	- maximum swelling equilibrium(%)—1243% - adsorption capacity (Q_max,_ mg/g)—184 mg Co/g (tc = 10 h, c_0_ = 250 mg/L) - best-fit kinetic—pseudo-second-order kinetic (R^2^ = 0.99) - best-fit isotherm—Freundlich isotherm (R^2^ = 0.99) - the negative values of ΔG^o^ and positive values of ΔH^o^ and ΔS^o^ indicating the spontaneous and endothermic nature of the adsorption	-	[121]
Ni^2+^, Cu^2+^, Zn^2+^, and Cr^3+^	- Monomer: AM, AA - Crosslinker: [Vim]Br_2_ - Initiator: APS	- by crosslinking free-radical polymerization	- maximum swelling equilibrium(%)—40% - removal efficiency (%)—values all exceeding 90% -adsorption capacity (Q_max,_ mg/g)—38.5 mg Ni/g_xerogel_ (tc = 3 h, c_0_ = 40 mg/L) and 89 mg Ni/g (tc = 10 h, c_0_ = 100 mg/L) - best-fit kinetic—pseudo-second-order kinetic (R^2^ = 0.998) - best-fit isotherm—Langmuir model (R^2^ = 0.995) at higher concentrations (100 ppm), and Freundlich isotherm at low concentrations (40 ppm)	-	[122]
Cu^2+^ Ba^2+^ and Sr^2+^	- Monomer: AM, AA - Crosslinker: NMBA - Initiator: Pn-In	- by crosslinking free-radical polymerization using the gamma irradiation technique	- maximum swelling equilibrium(%)—400% - adsorption capacity (Q_max,,_ mg/g)—36.4 mg Ba/g_,_(tc = 24 h, c_0_ = 100 mg/L), 27.31 mg Sr/g (tc = 24 h, c_0_ = 100 mg/L), 13.7 mg Cu/g, (tc = 24 h, c_0_ = 50 mg/L) - best-fit kinetic—pseudo-first-order kinetic - best-fit isotherm—Freundlich model (R^2^ = 0.90–0.992) -the thermodynamic parameters (ΔG^o^_,_ΔH^o^ andΔS^o^) indicated that the adsorption of ions exhibit both exothermic and endothermic characteristics	-	[123]
Cu^2+^, Ni^2+^ and Zn^2+^	- Monomer: AMPS - Crosslinker: NMBA - Initiator: APS, TEMEDA	- by free-radical polymerization	- removal efficiency (%)—all values exceeding 95% - adsorption capacity (Q_max,_ mg/g)—36 mg Zn/g, 33.1 mg Ni/g, 32.9 mg Cu/g (tc = 3 h, c_0_ = 100 mg/L) - best-fit kinetic—pseudo-second-order kinetic (R^2^ = 0.999)	-	[124]
Fe^3+^, Cu^2+^, Zn^2+^, and Ni^2+^	- Monomer: AMPSA - Crosslinker: EGDMA - Initiator: BPO, TMEDA	- by suspension polymerization	- removal efficiency (%) –97.7% Fe, 46.3% Ni, 42.6% Cu, 29.5% Zn - adsorption capacity (Q_max,_ mg/g)—60.9 mg Fe/g (tc = 2 h, c_0_ = 616 mg/L), 3.07 mg Cu/g (tc = 2 h, c_0_ = 72 mg/L), 1.62 mg Zn/g (tc = 2 h, c_0_ = 55 mg/L), 0.74 mg Ni/g (tc = 2 h, c_0_ = 16 mg/L) - best-fit kinetic—pseudo-second-order kinetic (R^2^ = 0.99)	-	[125]
Pb^2+^	- Monomer: AMPS, AA - Homopolymer: PVA - Crosslinker: EGDMA - Initiator: KPS, TMED	- by free-radical polymerization	- maximum swelling equilibrium(%)—8130% - removal efficiency (%)—90.2% - adsorption capacity (Q_max,_ mg/g)—200.7 mg Pb/g (tc = 8 h, c_0_ = 300 mg/L) - best-fit kinetic—pseudo-second-order kinetic (R^2^ = 0.996) - best-fit isotherm—Langmuir isotherm (R^2^ = 0.99) -the negative values of ΔG^o^ and positive values of ΔH^o^ and ΔS^o^ indicating the spontaneous and endothermic nature of the adsorption	-	[126]
Pb^2+^	- Monomer: NIPAM, AMPSA - Homopolymer: PVA - Crosslinker: NMBA - Initiator: APS	- by a surfactant-free emulsion polymerization	- maximum swelling equilibrium(%)—8130% - removal efficiency (%)—99% - adsorption capacity (Q_max,_ mg/g)—510.2 mgPb/g (tc = 4 h, c_0_ = 800 mg/L) - best-fit kinetic—pseudo-second-order kinetic (R^2^ = 0.99) - best-fit isotherm—Langmuir isotherm (R^2^ = 0.99) -the negative values of ΔG^o^ and_,_ ΔH^o^ indicate the spontaneous and exothermic nature of the adsorption	- desorption eluent: alkali solution - 4th regeneration run - 4th reusable run with recovery efficiency ranging from 95.7–104.4 for Pb^2+^ ions	[127]
La^3+^	- Monomer: NIPAM, MA - Filler: GO - Crosslinker: NMBA - Initiator: KPS, TEMED	- by free-radical cryopolymerization	- maximum swelling equilibrium(%)—28.76 g/g - removal efficiency (%)—99% - adsorption capacity (Q_max,_ mg/g)—33.1 mg La/g (tc = 12 h, c_0_ = 208.37 mg/L) - best-fit kinetic—pseudo-second-order kinetic (R^2^ = 0.98) - best-fit isotherm—Langmuir isotherm (R^2^ = 0.99)	- desorption eluent: HCl solution - 4th regeneration run - 4th reusable run with 94% recovery efficiency	[128]
Pb^2+^ and Cd^2+^	- Monomer: AA, MMA - Filler: GO - Crosslinker: NMBA - Initiator: APS, TEMED	- by free-radical crosslinking copolymerization	- removal efficiency (%)—98.5% - adsorption capacity (Q_max,_ mg/g)—2.225 mgPb/g and 4.175 mg Cd/g (tc = 2 h, c_0_ = 40 mg/L) - best-fit kinetic—pseudo-second-order kinetic (R^2^ = 0.99) - best-fit isotherm—Langmuir isotherm (R^2^ = 0.999) -the negative values of ΔG^o^ and positive values of ΔH^o^ and ΔS^o^ indicating the spontaneous and endothermic nature of the adsorption	- desorption eluent: 0.1 M HCl solution - 5th regeneration run - 5th reusable run with 91% and 87% recovery efficiency for Pb^2+^ and Cd^2+^, respectively	[48]
Cd^2+^	- Monomer: AM, AMPSA - Filler: MMT - Crosslinker: NMBA - Initiator: KPS, TEMED	- by free-radical polymerization	- maximum swelling equilibrium(%)—513.3% - removal efficiency (%)—99% - adsorption capacity (Q_max,_ mg/g)—301.5 mg Cd/g (tc = ~2 h, c_0_ = 500 mg/L) - best-fit kinetic—pseudo-second-order kinetic (R^2^ = 0.98) - best-fit isotherm—Langmuir isotherm (R^2^ = 0.99)	- desorption eluent: 1.0 M HNO_3_ - 5th regeneration run - 5th reusable run with 99% recovery efficiency	[129]
Pb^2+^	- Monomer: AA, AM - Filler: Lap-KCuHCF - Crosslinker: NMBA - Initiator: KPS	- by free-radical crosslinking copolymerization	- removal efficiency (%)—99% - adsorption capacity (Q_max,_ mg/g)—125 mg Pb/g (tc = 1 h, c_0_ = 500 mg/L) - best-fit kinetic—pseudo-second-order kinetic (R^2^ = 0.99) - best-fit isotherm—Langmuir isotherm (R^2^ = 0.99)	- desorption eluent: 1.0 M HNO_3_ - 5th regeneration run - 5th reusable run with 70% recovery efficiency	[130]
Cs^+^	- Monomer: AA, - Filler: Lap-KCuHCF - Crosslinker: NMBA - Initiator: V50	- by free-radical crosslinking copolymerization	- removal efficiency (%)—90% - adsorption capacity (Q_max,_ mg/g)—146.22 mg Cs/g (tc = ~2 h, c_0_ = 625 mg/L) - best-fit kinetic—pseudo-second-order kinetic (R^2^ = 0.99) - best-fit isotherm—Langmuir isotherm (R^2^ = 0.99)	- desorption eluent: 1.0 M HNO_3_ - 5th regeneration run - 5th reusable run with 80% recovery efficiency	[131]
Pb^2+^	- Monomer: AM, IA - Filler: MWCNTs - Crosslinker: NMBA - Initiator: APS	- by graft copolymerization	- maximum swelling equilibrium(%)—355% - removal efficiency (%)—99% - adsorption capacity (Q_max,,_ mg/g)—101.01 mg Pb/g (tc = 1.5 h, c_0_ = 175 mg/L) - best-fit kinetic—pseudo-second-order kinetic (R^2^ = 0.99) - best-fit isotherm—Langmuir isotherm (R^2^ = 0.98)	-	[132]
Cd^2+^	- Monomer: AA - Filler: AC - Crosslinker: NMBA - Initiator: KPS	- by free-radical crosslinking polymerization	- removal efficiency (%)—98.5% - adsorption capacity (Q_max,_ mg/g)—473.2 mg Cd/g (tc = 15 min, c_0_ = 1124 mg/L) - best-fit kinetic—pseudo-second-order kinetic (R^2^ = 0.999) - best-fit isotherm—Langmuir isotherm (R^2^ = 0.999) - the negative values of ΔG^o^ and positive values of ΔH^o^ and ΔS^o^ indicating the spontaneous and endothermic nature of the adsorption	-	[133]
Zn^2+^ and Cd^2+^	- Monomer: AMPSA, MA - Filler: Fe_3_O_4_ - Crosslinker: NMBA - Initiator: APS, TEMED	- by free-radical crosslinking copolymerization	- maximum swelling equilibrium(%)—5000% - adsorption capacity (Q_max,_ mg/g)—289.12 mg Zn/g and 385.2 mg Cd/g (tc = 2 h, c_0_ = 1000 mg/L) - best-fit kinetic—pseudo-second-order kinetic (R^2^ = 0.999) - best-fit isotherm—Freundlich isotherm (R^2^ = 0.99) - thermodynamic data showed the adsorption to be spontaneous and exothermic	-	[134]
Cr^3+^	- Monomer: AA, NIPAM - Homopolymer: PVP - Filler: FeCl_3_·6H_2_O, FeCl_2_·4H_2_O, TEOS - Crosslinker: NMBA - Initiator: APS	- by free-radical crosslinking copolymerization	- adsorption capacity (Q_max,_ mg/g)—243.90 mg Cr/g (tc = 24 h, c_0_ = 100 mg/L) - best-fit kinetic—pseudo-second-order kinetic (R^2^ = 0.995) - best-fit isotherm—Freundlich isotherm (R^2^ = 0.954)	-	[135]
Cu^2+^, Pb^2+^, Zn^2+^, and Cd^2+^	- Monomer: AA, AM - Filler: GO - Crosslinker: NMBA - Initiator: KPS	- by free-radical polymerization	- adsorption capacity (Q_max,_mg/g)—1268 mg Cu/g, 2026 mg Pb/g, 704 mg Zn/g, and 632 mg Cd/g (tc = 1 h, c_0_ = 2000 mg/L) - best-fit kinetic—pseudo-second-order kinetic (R^2^ = 0.99) - best-fit isotherm—Langmuir isotherm (R^2^ = 0.85)	- desorption eluent: HCl solution - 3rd regeneration run - 3rd reusable run with 88% recovery efficiency	[136]
Pb^2+^, Cd^2+^ and Cu^2+^	- Monomer: AA, AM - Filler: EMS - Crosslinker: NMBA - Initiator: APS	- by free-radical polymerization	- removal efficiency (%)—62% Pb, 75% Cd, and 78% Cu - adsorption capacity (Q_max,_ mg/g)—135.5 mg Pb/g (tc = 1 h, c_0_ = 207.2 mg/L), 134 mg Cd/g (tc = 1 h, c_0_ = 112.41 mg/L), and 54 mg Cu/g (tc = 1 h, c_0_ = 63.55 mg/L) - best-fit kinetic—pseudo-second-order kinetic (R^2^ = 0.99) - best-fit isotherm—Langmuir isotherm (R^2^ = 0.85) - the negative values of ΔG^o^ and positive values of ΔH^o^ and ΔS^o^ indicating spontaneous and endothermic nature of the adsorption	- desorption eluent: EDTA-2Na liquors and NaOH - 3rd regeneration run - 3rd reusable run with 70% recovery efficiency	[137]
Cu^2+^	- Homopolymer: PVA - Crosslinker: GA	- by chemical crosslinking reaction	- maximum swelling equilibrium(%)—250% - removal efficiency (%)—90% - adsorption capacity (Q_max,_ mg/g)—184 mg Co/g (tc = 2 h, c_0_ = 50 mg/L) - best-fit kinetic—pseudo-second-order kinetic (R^2^ = 0.999)	-	[138]
Pb^2+^ and Cd^2+^	- Monomer: AA, MADA - Filler: Ti_3_C_2_ - Crosslinker: NMBA - Initiator: APS	- by copolymerization	- adsorption capacity (Q_max,_ mg/g)—609 mg Pb/g and 250 mg Cd/g (tc = 2 h, c_0_ = 50 mg/L) - best-fit isotherm—Langmuir isotherm (R^2^ = 0.99)	- desorption eluent: HCl solution - 5th regeneration run - 5th reusable run with 95% recovery efficiency	[49]
Pb^2+^	- Monomer: AA - Filler: WT - Initiator: CAN	- by radical polymerization and microwave and/or UV irradiation	- maximum swelling equilibrium(%)—28.76 g/g - removal efficiency (%)—95% - adsorption capacity (Q_max,_ mg/g)—35.7 mg Pb/g (tc = 2 h, c_0_ = 200 mg/L) - best-fit kinetic—pseudo-first-order kinetic (R^2^ = 0.998) - best-fit isotherm—Langmuir isotherm (R^2^ = 0.992)	- desorption eluent: dilute nitric acid solution - 3rd regeneration run - 3rd reusable run with 91% recovery efficiency	[139]

‘—’ indicates that no value is available for this field.

**Table 4:** Chitosan-based hydrogels for various heavy metals’ removal from water or wastewater.

Heavy Metals	Chitosan-Based Hydrogel	Preparation Method	Performance	Regeneration and Reusability	Ref.
Cu^2+^, Ni^2+^	Glutamic-chitosan hydrogels	Crosslinking	- maximum swelling equilibrium:— - removal efficiency (%)—95.17% for Cu^2+^ and 48–95% for Ni^2+^ - adsorption capacity (Q_max,_ mg/g)—83.33 mg Cu/g and 103.4 mg Ni/g - best-fit kinetic—pseudo-second-order model (R^2^ = 0.988) - best-fit isotherm—Langmuir isotherm—(R^2^ = 0.999)	-	[164]
Cu^2+^	N-Aminorhodanine-modified chitosan hydrogel beads	Crosslinking	- maximum swelling equilibrium(%)—362%- removal efficiency (%):— - adsorption capacity (Q_max,_ mg/g)—62.5 mg Cu/g - best-fit kinetic—pseudo-second-order model (R^2^ = 0.9976) - best-fit isotherm—Freundlich isotherm (R^2^ = 0.967)	- desorption effluent: mixture of hydrochloric acid (HCl) and Ethylene diamine tetra-acetic acid sodium salt (EDTA) - 6th regeneration run - 6th reusable run with 23% removal efficiency	[165]
Pb^2+^, Hg^2+^, Cd^2+^, Cr^3+^	Gelatine–chitosan hydrogel particles	Crosslinking	- maximum swelling equilibrium(%)—7000–8000% - removal efficiency (%)—12% for Pb^2+^, 97% for Hg^2+^, 2% for Cd^2+^, and 24% for Cr^3+^ - adsorption capacity (Q_max,_ mg/g):— - best-fit kinetic:— - best-fit isotherm:—	-	[166]
Pb^2+^	Chitosan/alginate/Fe_3_O_4_@SiO_2_ hydrogel composites	By adjusting the ionic strength of chitosan solutions	- maximum swelling equilibrium:— - removal efficiency (%)—99% - adsorption capacity (Q_max,_ mg/g)—234.77 mg Pb/g - best-fit kinetic—Elovich model (R^2^ = 0.99) - best-fit isotherm—Langmuir isotherm	- desorption effluent: nitric acid (HNO_3_) solution - 3rd regeneration run - 3rd reusable run with 18% removal efficiency	[167]
Cu^2+^	Magnetic bentonite/carboxymethyl chitosan/sodium alginate hydrogel beads	Crosslinking	- maximum swelling equilibrium:— - removal efficiency (%)—93% - adsorption capacity (Q_max,_ mg/g)—56.79 mg Cu/g - best-fit kinetic—pseudo-second-order model (R^2^ = 0.999) - best-fit isotherm—Langmuir isotherm (R^2^ = 0.989)	- desorption effluent: HCl solution - 4th regeneration run - 4th reusable run with 80% removal efficiency	[168]
Pb^2+^, Cd^2+^, Cu^2+^	Gallic acid-modified carboxymethyl chitosan/iron ions hydrogels	Multi-crosslinking	- maximum swelling equilibrium—135% - removal efficiency (%):— - adsorption capacity (Q_max,_ mg/g)—97.15 mg Pb/g, 99.75 mg Cd/g, and 98.50 mg Cu/g - best-fit kinetic—pseudo-second-order model (R^2^ = 1) - best-fit isotherm—Langmuir isotherm (R^2^ = 0.977)	- desorption effluent: HCl solution - 10th regeneration run - 10th reusable run with 80% removal efficiency	[169]
Cu^2+^	Magnetic chitosan–alginate–magnetite hydrogel beads	Crosslinking	- maximum swelling equilibrium:— - removal efficiency (%)—56.51% - adsorption capacity (Q_max,_ mg/g):— - best-fit kinetic:— - best-fit isotherm:—	-	[170]
Cu^2+^	Chitosan-Based Beads Incorporating Inorganic–Organic Composites	Crosslinking	- maximum swelling equilibrium:— - removal efficiency (%)—29–52% - adsorption capacity (Q_max,_ mg/g)—17 mg Cu/g - best-fit kinetic—pseudo-second-order model (R^2^ = 0.898–0.989) - best-fit isotherm:—	-	[171]
Cd^2+^, Cr^3+^	*O*-Carboxymethyl chitosan/sodium alginate/zeolite hydrogel beads (ACZ) and *O*-Carboxymethyl chitosan/sodium alginate/Zn–Fe-layered double-hydroxides hydrogel beads (ACL)	Crosslinking	- maximum swelling equilibrium(%)—200%, 263%, and 272% for AC, ACZ, and ACL - removal efficiency (%)—86.3% (ACZ) and 88.9% (ACL) for Cd^2+^, 85.8% (ACZ) and 40.6% (ACL) for Cr^3+^ - adsorption capacity (Q_max,_ mg/g):— - best-fit kinetic—Avrami model (R^2^ = 0.99) - best-fit isotherm—Langmuir isotherm (R^2^ = 0.99)	- desorption effluent: HCl solution - 5th regeneration run - 5th reusable run with an adsorption capacity of 45 mg/g	[172]
Pb^2+^, Cu^2+^, Cd^2+^	Chitosan/sodium alginate/calcium ion hydrogel	Physically crosslinking	- maximum swelling equilibrium—11,000% - removal efficiency (%):— - adsorption capacity (Q_max,_ mg/g)—176.50 mg Pb/g, 70.83 mg Cu/g, and 81.25 mg Cd/g - best-fit kinetic—pseudo-first-order model (R^2^ = 0.994) and pseudo-first-order model (R^2^ = 0.994) - best-fit isotherm—Langmuir–Freundlich isotherm (R^2^ =0.99)	-	[173]
Pb^2+^, Cd^2+^, Cr^3+^, Hg^2+^	Chitosan–gelatin hydrogel particles	Inverse suspension	- maximum swelling equilibrium(%)—850% for oven-dried hydrogels and 6000% for freeze-dried hydrogels - removal efficiency (%)—73–94%- adsorption capacity (Q_max,_ mg/g):— - best-fit kinetic:— - best-fit isotherm:—	-	[174]

‘—’ indicates that no value is available for this field.

**Table 5:** Adsorption of dyes by different polysaccharide-based hydrogels.

Dyes	Polysaccharides Hydrogel Adsorbents	Preparation Method	Performance	Regeneration and Reusability	Ref.
CR	Cellulose–chitosan hydrogel beads	Extruding and blending regeneration	- maximum swelling equilibrium (%):— - removal efficiency (%)—89.6% - adsorption capacity (Q_max,_ mg/g)—40 mg CR/g - best-fit kinetic—pseudo-second-order model (R^2^ = 0.99) - best-fit isotherm—Langmuir isotherm (R^2^ = 0.97)	-	[191]
MO	Cellulose–chitosan/β-FeOOH composite hydrogels	Crosslinking	- maximum swelling equilibrium:— - removal efficiency (%)—60% - adsorption capacity (Q_max,_ mg/g):— - best-fit kinetic—pseudo-second-order model (R^2^ = 0.99) - best-fit isotherm—Langmuir isotherm (R^2^ = 0.998)	- desorption eluent: deionized water and ethanol - 5th regeneration run - 5th reusable run with 80.81% removal efficiency	[192]
AOII MB	Carboxymethyl cellulose–chitosan hydrogels	Crosslinking	- maximum swelling equilibrium (%)—7000% at pH 2 and 2000–3000% at pH 7 - removal efficiency (%)—90% for AOII and 95% for MB - adsorption capacity (Q_max,_ mg/g)—100 mg AOII/g and 110 mg MB/g - best-fit kinetic:— - best-fit isotherm:—	- desorption eluent:— - 3rd regeneration run - 3rd reusable run with 18% removal efficiency	[193]
CV TB	Carboxylmethyl cellulose/sodium alginate microgel spheres	Crosslinking	- maximum swelling equilibrium:— - removal efficiency (%)—96.84% for CV and 95.41% for TB - adsorption capacity (Q_max,_ mg/g)—160.87 mg CV/g and 141.48 mg TB/g - best-fit kinetic—pseudo-second-order model (R^2^ = 0.99) - best-fit isotherm—Sips isotherm (R^2^ = 0.977 for CV and R^2^ = 0.951 for TB)	- desorption eluent:— - 5th regeneration run - 5th reusable run with 96.84% for CV and 95.41% for TB removal efficiency	[194]
MB	Cellulose nanocrystal–alginate hydrogel beads	Crosslinking	- maximum swelling equilibrium (%)— - removal efficiency (%)—97% - adsorption capacity (Q_max,_ mg/g)—256.41 mg MB/g - best-fit kinetic: pseudo-second-order model—(R^2^ = 0.999) - best-fit isotherm: Langmuir isotherm—(R^2^ = 0.998)	- desorption eluent: -HCl and ethanol (EtOH) mixture - 5th regeneration run - 5th reusable run with 97% removal efficiency	[195]
MB MO	Sugar Beet Pulp Cellulose/Starch/Activated Carbon-ZnO hydrogels	Crosslinking and ultrasonic cavitation	- maximum swelling equilibrium (%):— - removal efficiency (%)—91.22% for MB and 90.44% for MO - adsorption capacity (Q_max,_ mg/g)—142.70 mg MB/g and 72.63 mg MO/g - best-fit kinetic—pseudo-second-order model (R^2^ = 0.99) - best-fit isotherm—Langmuir isotherm (R^2^ = 0.99 for MB and R^2^ = 0.997 for MO)	- desorption eluent: -EtOH and distilled water mixture - 5th regeneration run - 5th reusable run with 91.22% for MB and 90.44% for MO removal efficiency	[196]
DR80	Chitosan–starch hydrogels	Crosslinking	- maximum swelling equilibrium (%)—1500% - removal efficiency (%)—84.2% - adsorption capacity (Q_max,_ mg/g)—330.86 mg DR80/g - best-fit kinetic: pseudo-second-order model—(R^2^ = 0.99) - best-fit isotherm: Freundlich isotherm—(R^2^ = 0.99)	- desorption eluent: NaOH and HCl solution - 4th regeneration run - 4th reusable run with 75% removal efficiency	[197]
AOII	Chitosan–gelatin hydrogels	Crosslinking	- maximum swelling equilibrium (%):— - removal efficiency (%) - - adsorption capacity (Q_max,_ mg/g)—573 mg AOII/g - best-fit kinetic—pseudo-second-order model (R^2^ = 0.986) - best-fit isotherm:—	- desorption eluent: solutions with a fixed ionic strength - 7th regeneration run - 7th reusable run with 534 mg AOII/G adsorption capacity	[198]
MB MG	Chitosan–halloysite nanotube composite hydrogel beads	Dropping and pH precipitation	- maximum swelling equilibrium (%):— - removal efficiency (%)—95.2% for MB and 97.5% for MG - adsorption capacity (Q_max,_ mg/g)—270.27 mg MB/g and 303.03 mg MG/g - best-fit kinetic—pseudo-second-order model (R^2^ = 0.999) - best-fit isotherm—Langmuir (R^2^ = 0.998) and Freundlich isotherm (R^2^ = 0.994)	- desorption eluent: NaOH aqueous solution and acetone - 2nd regeneration run - 2nd reusable run with 93% removal efficiency for MB	[199]
RB-5	Nano-ZnO–Chitosan composite beads	Crosslinking (by ionic gelation)	- maximum swelling equilibrium (%):— - removal efficiency (%)—76% - adsorption capacity (Q_max,_ mg/g)—189.44 mg RB-5/g - best-fit kinetic:— - best-fit isotherm—Langmuir isotherm (R^2^ = 0.99)	-	[200]
FBL2 FR17	Activated carbon-based chitosan hydrogels	Crosslinking	- maximum swelling equilibrium (%):— - removal efficiency (%)—70% for FBL2 and 60% for FR17 - adsorption capacity (Q_max,_ mg/g)—155.1 mg FBL2/g and 133.9 mg FR17/g - best-fit kinetic—Avrami (R^2^ = 0.99) - best-fit isotherm:—	- desorption eluent: sodium chloride (NaCl) and NaOH solution - 5th regeneration run - 5th reusable run with 70% removal efficiency for FBL2 and 60% for FR17	[201]
Direct Red 83:1	Chitosan-Fe magnetic gels	Crosslinking	- maximum swelling equilibrium (%):— - removal efficiency (%):— - adsorption capacity (Q_max,_ mg/g)—17.46 mg Direct Red 83:1/g - best-fit kinetic—pseudo-second-order model (R^2^ = 0.999) - best-fit isotherm—Temkin isotherm (R^2^ = 0.946)	-	[202]
CR	Chitosan–hematite nanocomposite hydrogel capsules	Crosslinking (by anionic surfactant gelation)	- maximum swelling equilibrium (%):— - removal efficiency (%):— - adsorption capacity (Q_max,_ mg/g)—4705.6 mg CR/g - best-fit kinetic—pseudo-second-order model (R^2^ = 0.962) - best-fit isotherm—Langmuir isotherm (R^2^ = 0.90), Freundlich (R^2^ = 0.973) and Redlich–Peterson (R^2^ = 0.973)	-	[203]
MO CR	Carboxymethyl chitosan–phytic acid composite hydrogels	In situ polymerization	- maximum swelling equilibrium (%)—1277% - removal efficiency (%)—88.1% for MO and 89.7% for CR - adsorption capacity (Q_max,_ mg/g)—13.62 mg MO/g and 8.49 mg CR/g - best-fit kinetic—pseudo-first-order model (R^2^ = 0.998) - best-fit isotherm:—	- desorption eluent:— - 5th regeneration run - 5th reusable run with 88.1% removal efficiency for MO and 89.7% for CR	[204]
CR	Bentonite–chitosan-graft–gelatin nanocomposite hydrogels	Crosslinking	- maximum swelling equilibrium (%)—490.18% - removal efficiency (%)—93.85% - adsorption capacity (Q_max,_ mg/g)—453.87 mg CR/g - best-fit kinetic—pseudo-first-order model (R^2^ = 0.965) - best-fit isotherm—Langmuir isotherm (R^2^ = 0.974)	- desorption eluent: -NaOH and HCl solutions - 5th regeneration run - 5th reusable run with 88% removal efficiency	[205]
MB CR	Carboxyethyl chitosan/oxidized sodium alginate/Ca^2+^	Crosslinking (freeze–thaw technique)	- maximum swelling equilibrium (%)—11–21% - removal efficiency (%):— - adsorption capacity (Q_max,_ mg/g)—254.41 mg MB/g and 185.43 mg CR/g - best-fit kinetic—pseudo-second-order model (R^2^ = 0.999) - best-fit isotherm—Langmuir isotherm (R^2^ = 0.934) for MB and (R^2^ = 0.968) for CR	-	[206]
MB MO	Chitosan–carboxymethyl cellulose–Graphene oxide nanocomposites hydrogels	Crosslinking	- maximum swelling equilibrium (%):— - removal efficiency (%)—99% for MB and 82% for MO - adsorption capacity (Q_max,_ mg/g)—655.98 mg MB/g and 404.52 mg MO/g - best-fit kinetic—pseudo-second-order model (R^2^ = 0.999) - best-fit isotherm—Langmuir isotherm (R^2^ = 0.999)	- desorption eluent: HCl and NaOH solutions - 20th regeneration run - 20th reusable run with 93% removal efficiency for MB and 90% for MO	[207]
CR	Chitosan–rectorite–cellulose composite hydrogels	Crosslinking	- maximum swelling equilibrium (%)—72% - removal efficiency (%):— - adsorption capacity (Q_max,_ mg/g)—57.44 mg CR/g - best-fit kinetic—seudo-second-order model (R^2^ = 0.999) - best-fit isotherm—Freundlich isotherm (R^2^ = 0.994)	-	[208]
MO MB	Lignin–sodium alginate hydrogels	Crosslinking (ion exchange gelling process)	- maximum swelling equilibrium (%)—500% - removal efficiency (%)—91.15% - adsorption capacity (Q_max,_ mg/g)—388.81 mg MB/g - best-fit kinetic—pseudo-second-order model (R^2^ = 0.999) - best-fit isotherm—Freundlich isotherm (R^2^ = 0.984)	- desorption eluent: -HCl solution and EtOH - 5th regeneration run - 5th reusable run with 87.64% removal efficiency	[209]
MB MO	Activated organo-bentonite–sodium alginate beads	Crosslinking (ion exchange gelling process)	- maximum swelling equilibrium (%):— - removal efficiency (%):— - adsorption capacity (Q_max,_ mg/g)—1309 mg MB/g and 141 mg MO/g - best-fit kinetic—pseudo-second-order model (R^2^ = 0.99) - best-fit isotherm—Langmuir isotherm (R^2^ = 0.99)	-	[210]
MB RhB VG1 MO	Graphene oxide-incorporated alginate hydrogel beads	Crosslinking (ion exchange gelling process)	- maximum swelling equilibrium (%)—35% - removal efficiency (%):— - adsorption capacity (Q_max,_ mg/g)—240 mg MB/g, 250 mg RhB/g, 120 mg VG1/g, and 110 mg MO/g - best-fit kinetic—pseudo-second-order model-(R^2^ = —) - best-fit isotherm—Langmuir isotherm (R^2^ = 0.99)	-	[211]

‘—’ indicates that no value is available for this field.

**Table 6:** Dyes and composite hydrogels for their removal.

Dyes	Hybrid Hydrogel Precursors	Preparation Method	Performance	Regeneration and Reusability	Ref.
MB, Mordant blue 9, RB	Natural component: CMC Synthetic component: PAA, PANI Filler:—	Radical polymerization and crosslinking	- Removal efficiency (%): 97.5 (MB); 96 (RB), 74 (Mordant blue 9) - Adsorption capacity (Q_max_, mg/g): f or MB: 12.2 (Langmuir) and 1.65 (Freundlich) for Mordant Blue 9: 12.20 (Langmuir) and 1.65 (Freundlich for RB: 8.79 (Langmuir) and 2.80 (Freundlich) - Best-fit isotherm: Langmuir for MB and RB and Freundlich for Mordant blue 9	The hydrogel is 91.7% biodegraded in soil	[218]
MB	Natural component: Aldehyde-modified CMC Synthetic component: Hydrazine-modified PNIPAm Filler: GO	Freeze-drying and crosslinking	- Removal efficiency (%): 90.4 for highly concentrated MB solution (500 mg/L) and 60.4 for 200 mg/L MB solution - Adsorption capacity (Q_max_, mg/g): 601.7 for highly concentrated MB solution (500 mg/L) and 1622.1 for 200 mg/L MB solution - Best-fit kinetic: pseudo-second-order - Best-fit isotherm: Langmuir	76.3% reusability after 5 cycles	[220]
MB	Natural component: CMC Synthetic component: cPAA Filler:—	IPN	- Adsorption capacity (Q_max_, mg/g): 613 for MB. This hydrogel can also act on Cu, but with a lower adsorption capacity of 250 mg/g. - Best-fit kinetic: pseudo-second-order - Best-fit isotherm: Langmuir	After 5 adsorption–desorption cycles, the hydrogel retained 90% of its original removal capacity	[228]
MB, CV, BPB	Natural component: Cellulose macromonomer Synthetic component: PEG Filler:—	Williamson etherification with 4-vinyl benzylchloride and preparation of cellu-mers	- Adsorption capacity (Q_max_, mg/g): 5.3 for BPB; 8.5 for MB and 67.0 for CV Best-fit kinetic:— Best-fit isotherm:—		[230]
MB	Natural component: Cellulose Synthetic component:— Filler: GO	Heating–cooling–washing process	Maximum swelling equilibrium:— - Removal efficiency (%): 92.7 for TCH-GO10, 72.3 for TCH-GO5, 40.8 for TCH-GO2, 24.3 for TCH-GO1, 14.2 for TCH-GO0.5, and 9.1 for TCH - Adsorption capacity (Q_max_, mg/g): 46.4 for TCH-GO10, 36.2 for TCH-GO5, 20.4 for TCH-GO2, 12.1 for TCH-GO1, 7.1 for TCH-GO0.5, and 4.5 for TCH - Best-fit kinetic: pseudo-second-ordeBest-fit isotherm:—		[231]
Basic yellow 28	Natural component: HEC Synthetic component: PAA Filler:—	In situ free-radical graft copolymerization	- Maximum swelling equilibrium: >400% - Removal efficiency (%): 98% - Adsorption capacity (Q_max_, mg/g): 176.3 - Best-fit kinetic: pseudo-second-order - Best-fit isotherm: Langmuir		[246]
ST, BCB	Natural component: CMC Synthetic component: (1) AA/MBA, (2) sodium acrylate/MBA, and (3) comonomers AA/HEMA/MBA Filler:—	Free-radical polymerization	- Maximum swelling equilibrium:— - Removal efficiency (%): the best results were recorded for SPACMC system (94.5% for St and 98.9% for BCB) - Adsorption capacity (Q_max_, mg/g): 206.55 (PAACMC/ST); 209.33 (PAACMC/BCB); 186.65 (SPACMC/ST); 192.09 (SPACMC/BCB); 207.49 (CPCMC/ST); 196.24 (CPCMC/BCBest-fit kinetic:— Best-fit isotherm:—	After 5 consecutive cycles, the hydrogels maintained good mechanical properties under pH changes and thus a good recyclability	[247]
AR13, AB92, and AR112	Natural component: Quaternized cellulose Synthetic component: PEGDE Filler:—	PEGDE played a crosslinker role for the quaternized cellulose	- Maximum swelling equilibrium: 5.43 ± 0.21 (CCG1); 5.74 ± 0.22 (CCG2); 12.5 ± 0.22 - Removal efficiency (%): -Adsorption capacity (Q_max_, mg/g): 249 (AR13/CCG1); 286 (AR13/CCG2); 430 (AR13/CCG3); 269 (AB92/CCG1); 314 (AB92/CCG2); 447 (AB92/CCG3); 170 (AR112/CCG1); 209 (AR112/CCG2); 322 (AR112/CCG3) - Best-fit kinetic: pseudo-second-order - Best-fit isotherm: Langmuir	Recycling is possible as these materials can withstand changes in pH and temperature	[250]
MB, CR	Natural component: SA Synthetic component: maleic anhydride–acrylamide copolymer Filler:—	Radical polymerization and crosslinking	- Adsorption capacity (Q_max_, mg/g): 653 (MB), 685 (CR), 738 (Cr), and 754 (Cu) Best-fit kinetic:— Best-fit isotherm:— - This system is also suitable for heavy metals’ (Cr, Cu) removal	The material may be pretty well recycled, using for this purpose three cleaning agents: hydrochloric acid, ethanol, and deionized water	[213]
MB, CR	Natural component: Alg Synthetic component:— Filler:—	Calcium alginate spheres were prepared by approaching a microfluidic method	- Better removal performance recorded for MB (99%) instead of only 94 % for CR Best-fit kinetic:— Best-fit isotherm:—		[226]
MB, RB, MO	Natural component: SA Synthetic component:— Filler: GO- MMT	Crosslinking and freeze-drying	- Maximum swelling equilibrium:— - Removal efficiency (%): depending on MMT amount, the MB removal efficiency ranges from 79.02% to 96.77% - Adsorption capacity (Q_max_, mg/g): 150.66 - Best-fit kinetic: pseudo-second-order - Best-fit isotherm: Freundlich	A decrease in MB removal rate from 98.2 to 85.4% was found when increasing the reuse time, which proves repeated uses are enabled	[232]
RB	Natural component: SA Synthetic component: MA Filler: Trans-anethole	Interpenetrating network process	- Adsorption capacity (Q_max_, mg/g): 745 (RB), 734.9 (Pb), and 722 (Cd) - Best-fit kinetic: pseudo-second-order - Best-fit isotherm: Langmuir - This system is also suitable for heavy metals’ (Pb, Cd) removal	The adsorbed pollutant may be desorbed by soaking in water and ethanol	[234]
MG	Natural component: SA Synthetic component:—Poly(itaconic acid-co sodium 4—vinyl benzene sulfonate) Filler: Ricinus communis	Free-radical graft copolymerization	- Removal efficiency (%): 96.33% (pH = 7) - Adsorption capacity (Q_max_, mg/g): 1339.83 (pseudo-second-order) and 1247.36 (pseudo-first-order) - Best-fit kinetic: pseudo-second-order - Best-fit isotherm: Langmuir	Regeneration and reuse studies revealed that four continuous adsorption–desorption cycles can be performed	[239]
FY3	Natural component: SA Synthetic component: (PAA-co-PDMC) Filler:—	Free-radical graft copolymerization	- Maximum swelling equilibrium: 267.84 g/g - Removal efficiency (%): 98.23% - Adsorption capacity (Q_max_, mg/g): 654.87 - Best-fit kinetic: pseudo-second-order - Best-fit isotherm: Langmuir and Dubinin–Radushkevich	The removal efficiency after the 5th cycle was 90.23%, which is a good result	[249]
MG, MB, FS, RB, CV, O II, SY, MO	Natural component: CS Synthetic component: PVA Filler: carbon black	Freeze–thawing	- Removal efficiency (%): 98% Adsorption capacity (Q_max_, mg/g):— Best-fit kinetic:— Best-fit isotherm:—	-	[219]
CV	Natural component: CS Synthetic component: P(AM-IA) Filler:—	UV-initiated grafted copolymerization	-Removal efficiency (%):— Adsorption capacity (Q_max_, mg/g): 167.3 Best-fit kinetic:— Best-fit isotherm:—	-	[244]
IC; rhodamine 6G; SY	Natural component: CS Synthetic component: Poly(vinylbenzyl chloride-co-p-divinyl benzene)(poly(VBC-DVB)) Filler:-	Hypercrosslinked polymer (HCP) based on poly(VBC-DVB) was prepared by bulk polymerization, followed by hyper-crosslinking, followed by modification with CS by approaching phase inversion	Removal efficiency (%):— Adsorption capacity (Q_max_, mg/g):— Best-fit kinetic:— Best-fit isotherm:—	Regeneration possibility was determined by TGA. No significant weight loss was found after exposure to high temperatures, with it being considered that the material can be regenerated	[241]
AO 7, AR 18, AB 113 RY 17, RB 5, DB 78	Natural component: CS Synthetic component: Poly(methacrylic acid) Filler: TiO_2_	Inverse suspension polymerization	-	After three cycles of illumination removal, the material maintained more than 95% efficiency	[251]
MB	Natural component: salecan Synthetic component: P(AM-IA) Filler:—	Graft copolymerization	- Adsorption capacity (Q_max_, mg/g): 107.1 - Best-fit kinetic: pseudo-second-order - Best-fit isotherm: Freundlich	-	[227]
MB, RB	Natural component: pullulan Synthetic component: polyacrylamide Filler:—	Graft radical co-polymerization	- Maximum swelling equilibrium: 2063% after 24 h - Adsorption capacity (Q_max_, mg/g): 2 (RB) and 2.5 (MB) - Best-fit kinetic: pseudo-second-order - Best-fit isotherm: Langmuir	The adsorbent can be regenerated	[223]
CV	Natural component: pullulan Synthetic component: PDA Filler:—	Semi-IPN polymerization	- Adsorption capacity (Q_max_, mg/g): 107 - Best-fit kinetic: pseudo-second-order - Best-fit isotherm: Langmuir	HCl was used for desorption, whereas Na OH was used for regeneration. A high adsorption capacity (100 mg/g) was maintained after four cycles of adsorption experiments	[244]
MO	Natural component: salep Synthetic component: Poly(3-sulfopropyl acrylate-co- AA-co- AM) Filler:—	Grafted copolymerization	- Maximum swelling equilibrium - Removal efficiency (%) - Adsorption capacity (Q_max_, mg/g): 400 - Best-fit kinetic: pseudo-second-order - Best-fit isotherm: intra-particle diffusion	Five cycles of adsorption and reconditioning by drying at 40 °C	[236]
MB	Natural component: Carboxymethyl starch Synthetic component: Acrylic acid- acrylamide Filler:—	Graft polymerization and crosslinking	- Removal efficiency (%): 95 - Adsorption capacity (Q_max_, mg/g): 1700 - Best-fit kinetic: pseudo-second-order - Best-fit isotherm: Langmuir	Four adsorption–desorption cycles may be carried out, even though the efficiency decreased from 90% (in the 1st cycle) to 50% (in the last cycle)	[222]
MB	Natural component: carboxymethyl starch sodium Synthetic component: PVA Filler: magnetite	The polymers were dissolved in water and magnetite was in situ prepared	- Adsorption capacity (Q_max_, mg/g): 23.53 - Best-fit kinetic: pseudo-second-order - Best-fit isotherm: Freundlich	Good removal efficiency even after 8 cycles	[225]
MB	Natural component: Potato starch Synthetic component: PAA Filler:—	Gamma radiation-initiated copolymerization	- Adsorption capacity (Q_max_, mg/g): for MB, 5.93 for potato starch/AA/gamma radiation/KOH and 10.13 for potato starch/AA/gamma radiation; for Cr, 38.77 for potato starch/AA/gamma radiation/KOH and 33.31 for potato starch/AA/gamma radiation	-	[229]
MB, MV	Natural component: Gg Synthetic component: P(AAm-co-MAA) Filler:—	Microwave-assisted free-radical graft copolymerization	- Removal efficiency (%): 98 (MB) and 95 (MV) - Adsorption capacity (Q_max_, mg/g): 694.4 (MB, 25 °C), 724.63 (MB, 35 °C), and 746.26 (MB, 45 °C); 543.478 (MV, 25 °C), 609.756 (MV, 35 °C), and 645.161 (MV, 45 °C) - Best-fit kinetic: pseudo-second-order - Best-fit isotherm: Langmuir	The adsorbent was fully degraded in 50 days in soil compost	[212]
CR, BG, RB, MO	Natural component: Gg Synthetic component: polyacrylamide Filler:—	Microwave-assisted grafting	- Maximum swelling equilibrium(%): 2117% Removal efficiency (%):— - Adsorption capacity (Q_max_, mg/g): 179.09 (CR), 523.62 (BG), 421.60 (RB), and 173.69 (M)Best-fit kinetic:— - Best-fit isotherm: Langmuir	The soil-burial composting method showed that the adsorbent was degraded by 93% within 60 days	[233]
CR	Natural component: Gg Synthetic component: PAAm Filler: ZVI	Radical copolymerization	- Adsorption capacity (Q_max_, mg/g): 153.8 (25 °C), 200 (35 °C), and 250 (45 °C) - Best-fit kinetic: pseudo-second-order - Best-fit isotherm: Langmuir	The adsorbent may be involved in up to three cycles	[237]
MB, IC	Natural component: KG Synthetic component: poly(2-(dimethylamino)ethyl methacrylate) Filler:—	Microwave-assisted graft copolymerization	- Adsorption capacity (Q_max_, mg/g): 89.28 (MB) and 101.42 (IC) - Best-fit kinetic: pseudo-second-order - Best-fit isotherm: Freundlich	-	[217]
MG, RB	Natural component: KG Synthetic component: PAA Filler: SiC	In situ graft copolymerization	- Removal efficiency (%): 91% (MG) and 86% (RB) - Adsorption capacity (Q_max_, mg/g): 757.57 (MG) and 497.51 (RB) - Best-fit kinetic: pseudo-second-order - Best-fit isotherm: Langmuir	The adsorbent may be successfully involved within three adsorption–desorption cycles	[235]
CV	Natural component: XG Synthetic component: Poly(N-vinyl imidazole) Filler:—		- Adsorption capacity (Q_max_, mg/g): 453 - Best-fit kinetic: pseudo-first-order and intraparticle diffusion - Best-fit isotherm: Langmuir	Four adsorption–desorption cycles	[245]
MG	Natural component: GumT Synthetic component: HEMA Filler: TiO_2_	The copolymer was synthesized in its crosslinked form by microwave-assisted green polymerization technique	- Maximum swelling equilibrium(%): 396.9% - Removal efficiency (%): 99.3 - Adsorption capacity (Q_max_, mg/g): 78.24 (GumT-cl-HEMA, 25 °C), 88.57 (GumT-cl-HEMA, 35 °C), 85.25 (GumT-cl-HEMA, 45 °C), 83.82 (GumT-cl-HEMA/TiO_2_, 25 °C), 103.09 (GumT-cl-HEMA/TiO_2_, 35 °C), 92.08 (GumT-cl-HEMA/TiO_2_, 45 °C) -Best-fit kinetic: pseudo-second-order - Best-fit isotherm: Langmuir	For GumT-cl-HEMA/TiO_2_, the adsorption dropped from 99.3% (1^st^ cycle) to 94.8 (3rd cycle). For GumT-cl-HEMA/TiO_2_, the adsorption dropped from 91.3% (1^st^ cycle) to 84.2 (3rd cycle)	[240]
MB	Natural component: Lignosulfonate (LS) Synthetic component: Poly(acrylic acid-r-acrylamide) Filler:—	Radical polymerization	- Adsorption capacity (Q_max_, mg/g): 2013 - Best-fit kinetic: pseudo-second-order - Best-fit isotherm: Freundlich	-	[216]
MB	Natural component: lignin Synthetic component: Poly(N-methyl aniline) Filler: GO	Polymerization of N-methylaniline in the presence of lignin in an aqueous solution	Maximum swelling equilibrium:—Removal efficiency (%):— - Adsorption capacity (Q_max_, mg/g): 201.7 (MB) and 753.5 (Pb) - Best-fit kinetic: pseudo-first-order Best-fit isotherm:— - The adsorbent can also be used for heavy metals (Pb)	Five adsorption–desorption cycles, using ethanol as desorbent	[224]
MO, MB, RB, p-nitrophenol	Natural component: carboxylated lactose/sodium lignosulfonate Synthetic component: PAA Filler: AgNO_3_	Self-assembly and in situ reduction	- Removal efficiency (%): conversion rate was above 80% after 540 min Adsorption capacity (Q_max_, mg/g):— - Best-fit kinetic: quasi-first-order Best-fit isotherm:—	Even after 8 cycles, an excellent performance was maintained (99.7%)	[221]
MG	Natural component: LS Synthetic component: Poly(acrylic acid-r-acrylamide) Filler:—	Radical grafting copolymerization	Maximum swelling equilibrium:— Removal efficiency (%):— - Adsorption capacity (Q_max_, mg/g): 107.066 (15 °C); 120.919 -(25 °C); and 150.376 (35 °C) - Best-fit kinetic: pseudo-second-order - Best-fit isotherm: Langmuir	-	[238]
CV	Natural component: gelatin Synthetic component: PAAm Filler: ACL/Mg-Fe LDH	Grafted copolymerization	Maximum swelling equilibrium:— - Removal efficiency (%): 92.81 (PAM-g-gelatin), 95.71 (PAM-g-gelatin/ACL), and 98.25 (PAM-g-gelatin/ACL/Mg-Fe LDH) - Adsorption capacity (Q_max_, mg/g): 35.45 (PAM-g-gelatin), 39.865 (PAM-g-gelatin/ACL), and 44.952 (PAM-g-gelatin/ACL/Mg-Fe LDH) - Best-fit kinetic: pseudo-second-order - Best-fit isotherm: Langmuir	-	[242]

‘—’ indicates that no value is available for this field.

**Table 7:** Heavy metals and composite hydrogels for their removal.

Heavy Metals	Hybrid Hydrogel Precursors	Preparation Method	Performance	Regeneration and Reusability	Ref.
Ni, Cd, Pb	Natural component: cellulose Synthetic component: AA Filler:—	Graft copolymerization	Maximum swelling equilibrium:— -Removal efficiency (%) - Adsorption capacity (Q_max_, mg/g): 825.7 (Pb^2+^), 562.7 (Cd^2+^), and 380.1 (Ni^2+^) - Best-fit kinetic: pseudo-second-order - Best-fit isotherm: Langmuir	Desorption was achieved using HNO_3_ 0.1 M. After 3 cycles, a 15% efficiency loss was recorded	[263]
Pb, Zn	Natural component: CMC Synthetic component: PAA Filler: MMT	Free-radical and solution polymerization	Maximum swelling equilibrium:— Removal efficiency (%) - Adsorption capacity (Q_max_, mg/g): 286.67 (Zn^2+^), and 146.19 (Pb^2+^) Best-fit kinetic:— Best-fit isotherm:—	Two cycles can be approached upon regeneration in HNO_3_ (0.1 M)	[266]
Cr, Pb, Cd, Cu	Natural component: Alg or Alg-CS Synthetic component: PVA Filler:—	-	Maximum swelling equilibrium:— Removal efficiency (%):— - Adsorption capacity (Q_max_, mg/g): 139.37 (PVA-Alg, Pb), 86.14 (PVA-Alg-CS, Cr) - Best-fit kinetic: pseudo-second-order - Best-fit isotherm: Langmuir	5 adsorption cycles which retained their efficiency (over 85%)	[264]
Cu	Natural component: Alg Synthetic component: Poly(2-acrylamido-2-methyl-1-propane sulfonic acid) Filler: NiFe_2_O_4_	-	Maximum swelling equilibrium:— - Removal efficiency (%): 83 (Cu) and 98.32 (MB) - Adsorption capacity (Q_max_, mg/g): 22.81 (Cu) and 275.6 (MB) - Best-fit kinetic: pseudo-second-order - Best-fit isotherm: Freundlich - MB can be also adsorbed using this system, in which case, both Langmuir and Freundlich fit	-	[272]
Cr	Natural component: CS Synthetic component: PAA Filler:—	Radical polymerization	Maximum swelling equilibrium:— - Removal efficiency (%): 94.72 - Adsorption capacity (Q_max_, mg/g): 73.14 (Langmuir) and 93.03 (Sips) - Best-fit kinetic: pseudo-*n*th-order - Best-fit isotherm: Redlich Peterson	-	[252]
Cr	Natural component: CS Synthetic component: PVA Filler: activated carbon		Maximum swelling equilibrium:— Removal efficiency (%):— - Adsorption capacity (Q_max_, mg/g): 109.89 - Best-fit kinetic: pseudo-second-order - Best-fit isotherm: Langmuir	-	[253]
Cu, Pb, Cd, Ni	Natural component: dextran- CS Synthetic component: PAA Filler:—	Ultrasonic heating	Maximum swelling equilibrium:— Removal efficiency (%):— - Adsorption capacity (Q_max_, mg/g): 395 (Pb), 342 (Cu), 269 (Cd), 232 (Co), and 184 (Ni) - Best-fit kinetic: pseudo-second-order - Best-fit isotherm: Langmuir	NaOH was used for desorption	[257]
Cu, Pb, Zn	Natural component: ketoglutaric acid grafting CS Synthetic component: Polyacrylamide crosslinked with MBA Filler:—	Semi-IPN	Maximum swelling equilibrium:— - Removal efficiency (%) was determined for a mixture consisting of five heavy metals, the following values being determined: 56 (Cu), 53 (Pb), and 38 (Zn) - Adsorption capacity (Q_max_, mg/g): 72.39 (Cu^2+^), 61.41 (Pb^2+^), and 51.89 (Zn^2+^) - Best-fit kinetic: pseudo-second-order - Best-fit isotherm: Langmuir	Even after 5 cycles, the adsorption capacity was above 90%	[259]
Cu, Pb, Cd	Natural component: CS Synthetic component: polyacrylamide Filler:—	AAm was radically polymerized within a CS solution	Maximum swelling equilibrium:— Removal efficiency (%) - Adsorption capacity (Q_max_, mg/g): 86.00 (Cd), 99.44 (Cu), and 138.41 (Pb) - Best-fit kinetic: pseudo-second-order - Best-fit isotherm: Langmuir	5 cycles	[265]
Pb, Cd	Natural component: CS Synthetic component: PAA Filler: Fe_3_O_4_	Pickering emulsion	Maximum swelling equilibrium:— Removal efficiency (%):— - Adsorption capacity (Q_max_, mg/g): 695.22 (Pb) and 308.84 (Cd) - Best-fit kinetic: pseudo-second-order - Best-fit isotherm: Langmuir	A high adsorption capacity was maintained after 5 cycles	[267]
Cd	Natural component: Carboxymethyl CS Synthetic component: PAA Filler: Fe_3_O_4_	Core–shell	Maximum swelling equilibrium:— Removal efficiency (%) - Adsorption capacity (Q_max_, mg/g): 155.10 Best-fit kinetic:— - Best-fit isotherm: Langmuir	-	[269]
Sn, Pt	Natural component: CS Synthetic component: AMPS, AA, and PEI Filler: GO; silica	Free-radical copolymerization	Maximum swelling equilibrium:— Removal efficiency (%):— - Adsorption capacity (Q_max_, mg/g): 263.16 (Pt) and 118.88 mg/g (Sn) - Best-fit kinetic: pseudo-first-order - Best-fit isotherm: Langmuir	After three adsorption–desorption cycles, only a slight decrease (less than 10%) in activity occurred	[271]
Cu	Natural component: starch Synthetic component: PAA Filler: Sodium humate (SH)	Radical graft copolymerization	Maximum swelling equilibrium:— Removal efficiency (%):— - Adsorption capacity (Q_max_, mg/g): 2.80 (0%SH); 2.83 (5%); 2.75 (10%); 2.73 (15%); 2.63 (10%) - Best-fit kinetic: pseudo-second-order - Best-fit isotherm: Langmuir	Regeneration can be carried out using NaOH and 4 cycles may be carried out	[260]
Cu, Cd, Ni, Zn	Natural component: potato starch Synthetic component: PAAm Filler:—	Redox-initiated graft copolymerization	Maximum swelling equilibrium:— Removal efficiency (%):— - Adsorption capacity (Q_max_, mg/g): 40.72 (Cu^2+^), 19.27 (Cd^2+^), 9.31 (Ni^2+^), 7.48 (Zn^2+^) Best-fit kinetic:— - Best-fit isotherm: Sips	Desorption with 0.1 M HCl	[261]
Cd	Natural component: amino-functionalized starch Synthetic component: PAA Filler:—	A double-network hydrogel with adsorbent features was elaborated	Maximum swelling equilibrium:— - Removal efficiency (%):— - Adsorption capacity (Q_max_, mg/g): 256.4 Best-fit kinetic:— - Best-fit isotherm: Langmuir	Desorption with 0.1 M NaOH	[270]
Cr	Natural component: cotton Synthetic component: Poly(acrylamide–acrylic acid) Filler:—	Free-radical graft copolymerization	- Maximum swelling equilibrium: 50 mg/g corresponded to the optimum cotton concentration of 120 wt% Removal efficiency (%):— - Adsorption capacity (Q_max_, mg/g): 353 (Cr) and 255 (Cu)	-	[255]
Ni, Cd	Natural component: XG Synthetic component: Poly(acrylamide-co-acrylic acid) Filler: GO	Free-radical polymerization	Maximum swelling equilibrium:— Removal efficiency (%):— - Adsorption capacity (Q_max_, mg/g): 312.15 (Cd^2+^, single-ion affinity), 185.0 (Ni^2+^, single-ion affinity), 259.25 (Cd^2+^, dual-ion affinity), 80.75 (Ni^2+^, dual-ion affinity) - Best-fit kinetic: pseudo-second-order - Best-fit isotherm: Langmuir	4 adsorption–desorption cycles with adsorption capacity decreased by less than 20%	[264]
Cu	Natural component: natural rubber Synthetic component: AA Filler:—	Radical copolymerization	Maximum swelling equilibrium:— - Removal efficiency (%): 72.19 Adsorption capacity (Q_max_, mg/g):— Best-fit kinetic:— Best-fit isotherm:—	-	[258]
Cu, Zn	Natural component: gelatin Synthetic component: PVA Filler: SPIONs		Maximum swelling equilibrium:— Removal efficiency (%):— - Adsorption capacity (Q_max_, mg/g): 47.594 (Cu, SPIONs-gelatin) and 40.559 (Zn, SPIONs-gelatin); 56.051 (Cu, SPIONs-gelatin-PVA) and 40.865 (Zn, SPIONs-gelatin-PVA)	-	[262]
Cd	Natural component: CadRP Synthetic component: PNIPAm Filler: SPIONs		Maximum swelling equilibrium:— Removal efficiency (%):— Adsorption capacity (Q_max_, mg/g):— Best-fit kinetic:— Best-fit isotherm:—	The adsorbent could capture and release Cd reversibly, which allows for at least 5 cycles	[268]

‘—’ indicates that no value is available for this field.

## Data Availability

No new data were created or analyzed in this study.

## Abbreviations

AA	acrylic acid	KG	Karaya gum
AB 113	Acid Blue 113	KPS	Potassium persulphate
AC	activated carbon	Lap	Laponite
AETAC	2-(acryloyloxy)ethyl trimethylammonium chloride	LS	Lignosulfonate
AHPS	3-Allyloxy-2-hydroxy-1-propane sulfonic acid sodium salt	MA	Maleic acid
AIBN	azobisisobuteronitrile	MAA	Methacrylic acid
AIPH	2,2′-azobis [2-(2-imidazolin-2-yl)propane] dihydrochloride	MAAn	Methacrylic anhydride
AM	acrylamide	MAC	[2-(Methacryloxy) ethyl] trimethyl ammonium chloride
AMPSA	2-acrylamide-2-methylpropane sulfonic acid	MADA	Methacrylamide dopamine
ANi	aniline	MB	Methylene Blue
AO	Auramine O	MBA	N,N-methylenebisacrylamide
AO7	Acid Orange 7	MEMA	2-(N-morpholino ethyl methacrylate)
APS	Ammonium persulfate	MEMA@GO	2-(N-morpholino ethyl) methacrylate/graphene oxide
APTMACl	(3-acrylamidopropyl) trimethyl ammonium chloride	MG	Malachite Green
APTES	aminopropyltrimethoxysilane	MMA	Methyl methacrylate
AR 18	Acid Red 18	MMT	Montmorillonite
AR	Alizarin Red	MO	Methyl orange
ARS	Alizarin red S	MOFs	Metal–organic frameworks
AY36	Acid Yellow 36	MPS	2-Acrylamido-2-methyl-1-propanesulfonic acid
BCB	Brilliant Cresyl Blue	MV	Methyl Violet
BIO	Biochar	MWCNTs	Multi-walled carbon nanotubes
BIO-Ox	Oxidized biochar	NA	N-allylisatin
BG	Brilliant Green	NIPAM	N-isopropyl acrylamide
BMB	Bromophenol Blue	NMBA	N,N-methylenebisacrylamide
BPO	Benzoyl peroxide	NVP	1-vinyl-2-pyrrolidinone
BzO_2_	Benzoyl peroxide	OG	Orange G
BY28	Basic yellow 28	O II	Orange II sodium Salt
CA	Carminic acid	PAA	Polyacrylic acid
CAN	Ceric Ammonium Nitrate	PCL	ε-caprolactone
CCA	Calconcarboxylic Acid	PDMAEMA	2-(dimethylamino) ethyl methacrylate
CCB	Coomassie brilliant blue	P(DMC-co-AM)	Poly (methacrylatoethyl trimethyl ammonium chloride-co-acrylamide)
CG	Chrysoidine G	P(DMAEMA)	Poly(2-(dimethylamino) ethyl methacrylate)
CMC	Carboxymethyl cellulose	PEG1500	Poly(ethylene glycol)1500
CMS	Carboxymethyl starch	PEGDA	Polyethylene glycol diacrylate
CNS	Calcium hydroxide nano-spherulites	PEGDMA	Poly (ethylene glycol) dimetacrylate
CNT	Carbon nanotubes	PEI	Polyethyleneimine
cPAA	Crosslinked PAA	Ph-In	2-hydroxy-4′-(2-hydroxyethoxy)-2-methylpropiophenone
CR	Congo Red	PHPAm	Partially hydrolyzed polyacrylamide
Cur	Curcuma longa rhizome	PLA	Polylactic acid
CV	Crystal Violet	PQ-10	Polyquaternium-10
DADMAC	Diallyl dimethylammonium chloride	PTE	Pentaerythritol Tetraallyl Ether
Darocur117	3 2-hydroxyl-2-methylpropiophenone	PVA	Polyvinyl alcohol
DB 15	Direct Blue 15	PVP	Polyvinylpyrrolidone
DB 78	Direct Blue 78	RBBR	Remazol Brilliant Blue R
DMAEMA	2-(Dimethylamino)ethyl methacrylate	RBP-5	Reactive black 5
DMC	methacrylatoethyl trimethyl ammonium chloride	RhB	Rhodamine B
2DMMT	Two-dimensional montmorillonite nanosheets	RPGS	Palygorskite clay
DR23	Direct Red 23	SA	Sodium alginate
DY	Direct yellow	SH	Sodium humate
EB	Eosin B	SO	Safranin O
EBT	Eriochrome Black T	ST	Safranin T
EDA	Ethylenediamine	SY	Sunset Yellow
EG	Ethylene glycol	TB	Trypan Blue
EGDMA	N,N- ethylene glycol dimethacrylate	TEMED	N,N,N′,N′-tetramethylethylenediamine
EMS	Electrolytic manganese slag	TEOS	Tetraethyl orthosilicate
EPC	Epichlorohydrin	TMPTA	1,1,1-trimethylolpropane trimethacrylate
FBL2	Food Blue 2	TiO_2_	Titanium dioxide
FeCl_2_·4H_2_O	Ferrous chloride tetrahydrate	VBC	4 vinylbenzyl chloride
FeCl_3_·6H_2_O	Ferric chloride hexahydrate	KG	Karaya gum
FR17	Food Red 17	KPS	Potassium persulphate
FS	Fluorescein sodium salt	Lap	Laponite
GA	Glutaraldehyde	LS	Lignosulfonate
Gg	Gum ghatti	MA	Maleic acid
GO	Graphene oxide	MAA	Methacrylic acid
GOX	lyoxal	MAAn	Methacrylic anhydride
GumT	Tragacanth gum	MAC	[2-(Methacryloxy) ethyl] trimethyl ammonium chloride
HHMP	2-hydroxy-4′-(2 hydroxyethoxy)-2-methyl-propiophenone	MADA	Methacrylamide dopamine
HEC	Hydroxyl ethyl cellulose	WT	Waste textiles
HEMA 2	Hydroxyethyl methacrylate	XG	Xantam gum
IA	Itaconic acid	γ-MPS	γ-methacryloxypropyltrimethoxysilane
IA-g-poly (AA-co-Ani)	Poly (acrylic acid-co-aniline)	ZnO	Zinc oxide
ILA-1VIM	Ionic liquid vinylimidazole	ZSM-5	Zeolite
IC	Indigo Carmine	VG1	Vat Green 1
KCuHCF	Potassium copper hexacyanoferrate	VOCs	Volatile organic compounds
		V50	2′-azobis(2-amidinopropane dihydrochloride)
		[Vim]Br_2_	Bis1-vinylimidazole ethyl bromide
